# Supplementary material for: Split RNA switch orchestrates pre- and post-translational control to enable cell type-specific gene expression
Source: Nat Commun. 2025 Jul 1;16:5362. doi: 10.1038/s41467-025-60392-2 (PMC12219281; doi:10.1038/s41467-025-60392-2)
Supplement: Supplementary file 1 — Supplementary Information [file 41467_2025_60392_MOESM1_ESM.pdf]

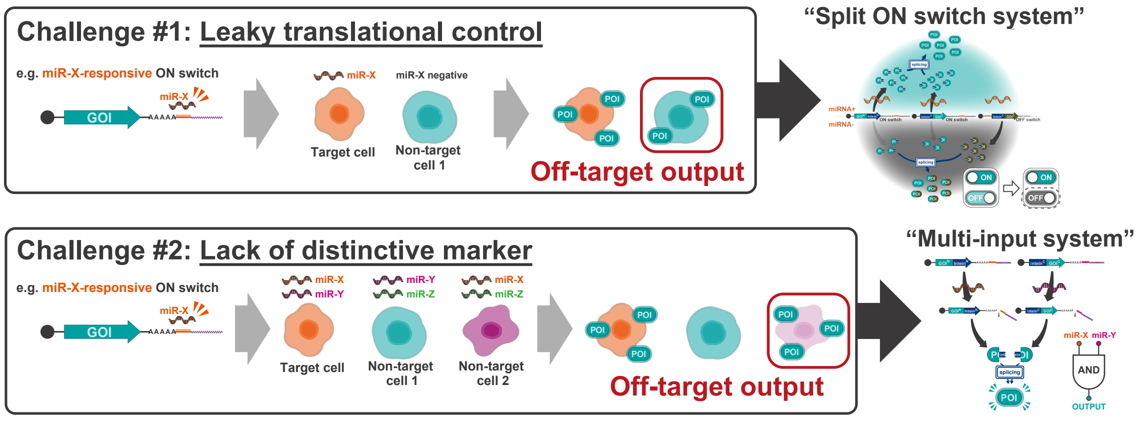

Supplementary Figure 1: Split RNA switch system can overcome two challenges that impair target cell specificity of RNA switch.

In general, translational control by a single RNA switch that targets one biomolecule (e.g., miRNA or protein) is insufficient for achieving high cell type-specificity due to two major challenges.

The first challenge is the undesired leaky expression of output protein in the OFF state ("leaky translational control"). To illustrate this issue, consider an experiment in which a miR-1-responsive ON switch is introduced into a mixed population of target cells (miR-1 activity positive, orange) and non-target cells (miR-1 negative, light blue) with the aim of expressing POI only in target cells. With leaky translation control, the POI is translated to some extent in non-target cells, resulting in a reduced ON/OFF ratio of the output protein level. Even in an "ideal" situation where only target cells in the population have the target miRNA activity and non-target cells have none, the leaky translation in the OFF state of the RNA switch itself prevents efficient fluorescent protein-based classification or cell fate control gene-based purification of target cells based on miRNA activity.

The second challenge is the difficulty in finding a target biomolecule whose expression level in the target cell type is so different from all other cell types that an RNA switch can clearly distinguish the cell ("lack of distinctive marker"). This issue is particularly problematic when trying to identify or select only target cells from a heterogeneous population of multiple cell types. The example at the bottom of the figure illustrates an experiment in which a miR-1-responsive ON switch is introduced into a mixed population of three cell types: target cells (miR-1 and miR-2 positive, orange), non-target cell 1 (miR-2 and miR-3 positive, light blue), and non-target cell 2 (miR-1 and miR-3 positive, purple). In this case, because miR-1 is also expressed in non-target cell 2, even if the miR-1-responsive ON switch is an "ideal" ON switch that overcomes the aforementioned "leaky translational control" challenge, inducing no POI expression in miR-1-negative cells, it is impossible to produce POI specifically in only the target cells. To achieve

specific POI expression in target cells using a single miRNA-responsive ON switch, one must find a miRNA species with high activity exclusively in the target cells, which is difficult or impossible. The split RNA switch provides a robust solution to these two challenges. The implementation of systems such as the "split ON switch system" (Fig. 2-5) and the "split toggle-like system" (Fig. 6) using the split RNA switch can effectively address the "leaky translational control" challenge by eliminating undesired protein output in the OFF state post-translationally. In addition, the split RNA switch approach enables the construction of a "multi-input system" (Fig. 7 and 8) that incorporates multiple target miRNAs as inputs, thereby offering a powerful solution to the "lack of distinctive markers" challenge.

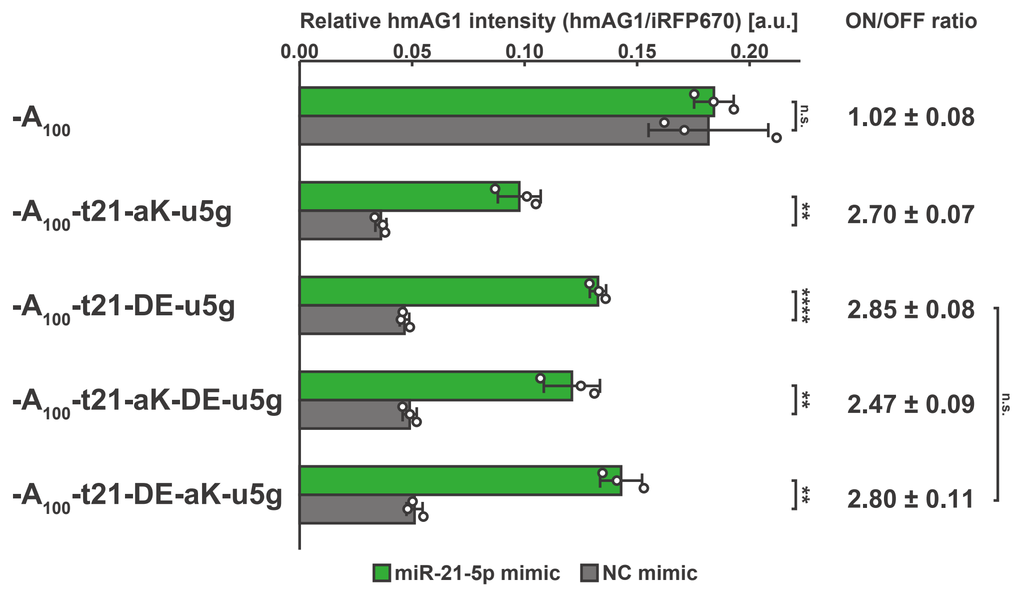

t21 (targeting sequence to hs-miR-21) : ucaacaucagucugauaagcua

aK (anti-Kozak sequence) : ccauggugge

DE (Decapping enzyme-binding element*) : accuccggaaaccguagauuccgggcggucggagccgccgggagcuguaguucucccgcg

u5g (decapping-enhanchment sequence**) : uuuuug

Supplementary Figure 2: Investigation of the extra sequence of ON switches in HEK293FT cells.

Designed miRNA-responsive ON switches with various sequences composed of “anti-Kozak” sequence and the recognition site for human Dcp2, a decapping enzyme (DE: Decapping enzyme-binding element)[1], arranged in varying orders, with u5g added at their 3’ ends as a decapping-enhancement sequence[2]. We evaluated translation levels of each ON switch with or without the miRNA activity in a reporter assay using hmAG1 as the output and iRFP670 as a reference. The bar graph shows relative hmAG1 intensity (hmAG1/iRFP670) of HEK293FT cells treated with miR-21-5p or negative control (NC) mimics. a.u., arbitrary units. The ON/OFF ratios of all switches were below three-fold, comparable to a previous study. Error bars represent means ± SD (n=3), and data of each biological replicate are shown as a point. Statistical analysis by two-sided Welch’s *t*-test, ***P*<0.01, *****P*<0.0001, n.s.: not significant (*P*>0.05). Each *P*-value is listed in Supplementary Table 4. Source data are provided as a Source Data file.

In this study, we used the ON switch with the “-t21-DE-aK-u5g” extra sequence, which exhibited the highest translational activity in the ON state in this assay. We prioritized the ON level of the ON switch because it was initially considered that controlling the target gene with a split ON switch might reduce the ON level of protein activity.

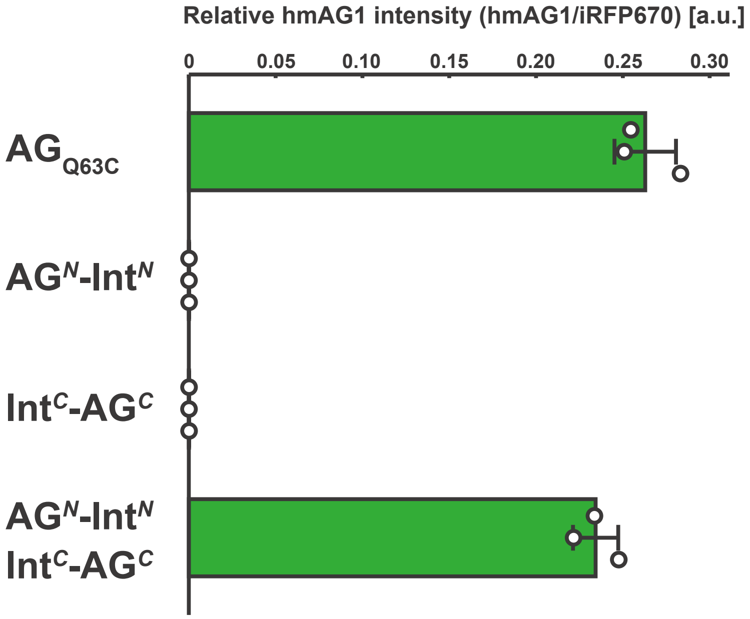

Supplementary Figure 3: Evaluation of protein splicing in HEK293FT cells.
The hmAG1 reporter assay was performed for the condition with mRNA coding protein fragments indicated on the left side of the bar graph. a.u., arbitrary units. Error bars represent means ± SD (n=3), and data of each biological replicate are shown as a point. Source data are provided as a Source Data file.

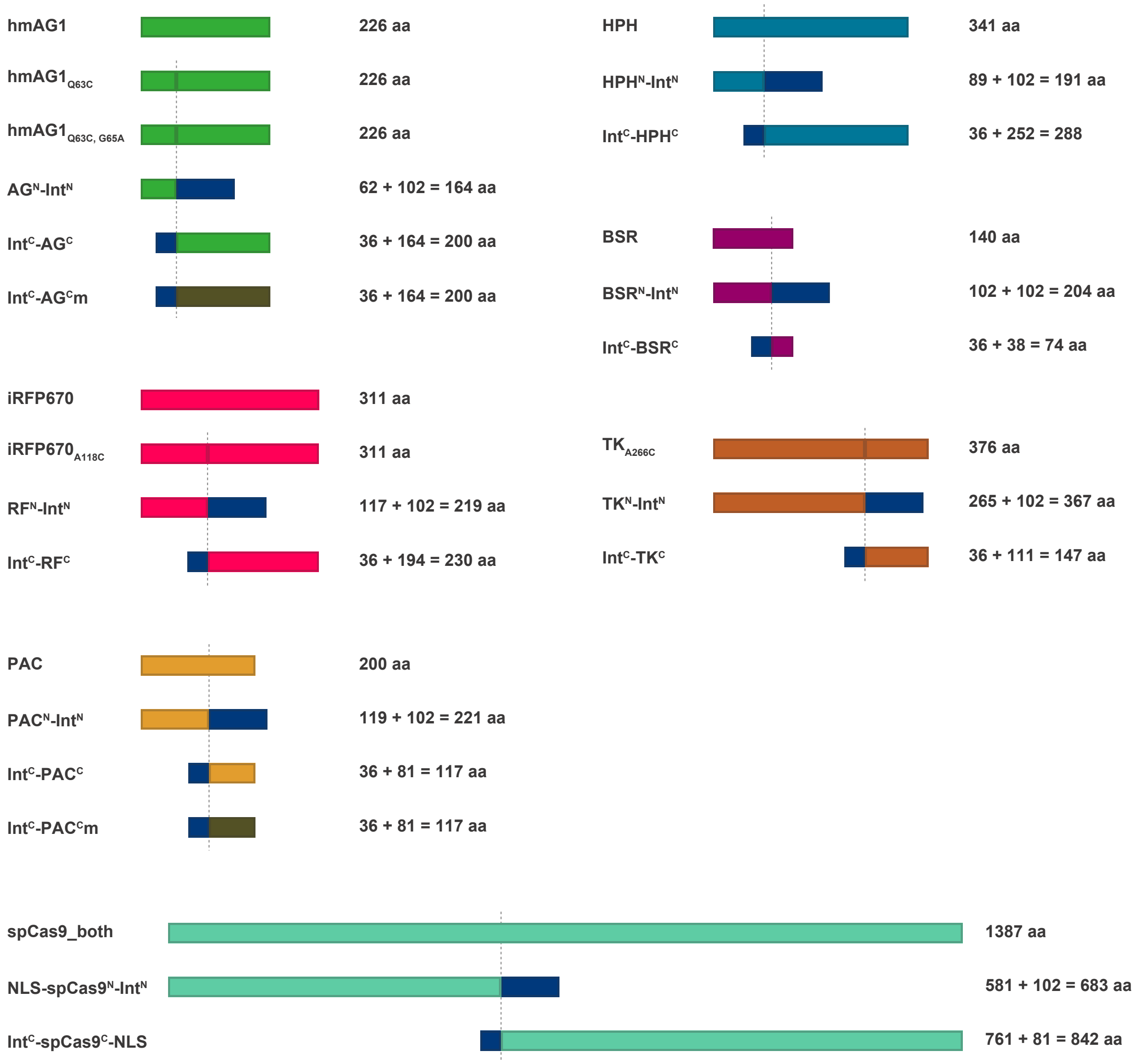

Supplementary Figure 4: The split sites of ORFs used in this research.

Schematic illustration for the split sites of the ORFs used in this research. The ORF sequence information is presented in Supplementary Table 1. The split sites of PAC, HPH, BSR, and TK are based on previous studies[3,4].

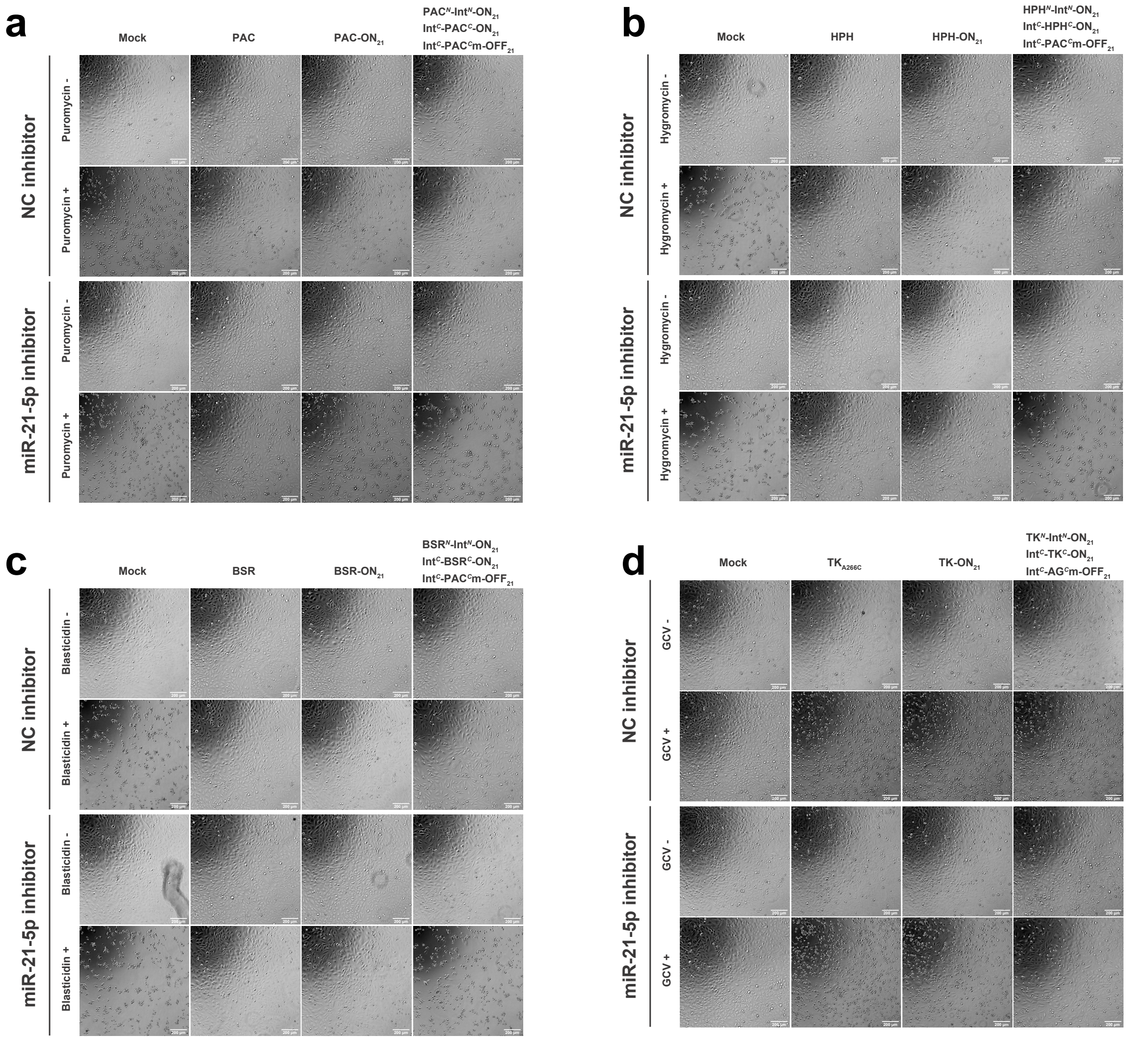

Supplementary Figure 5: Microscopic images of the split-PAC, -HPH, and -BSR switch system.

Representative microscopic bright-field images of HeLa cells transfected under the same conditions as shown in **Fig. 3b, d, f, h**. Scale bar, 200 µm.

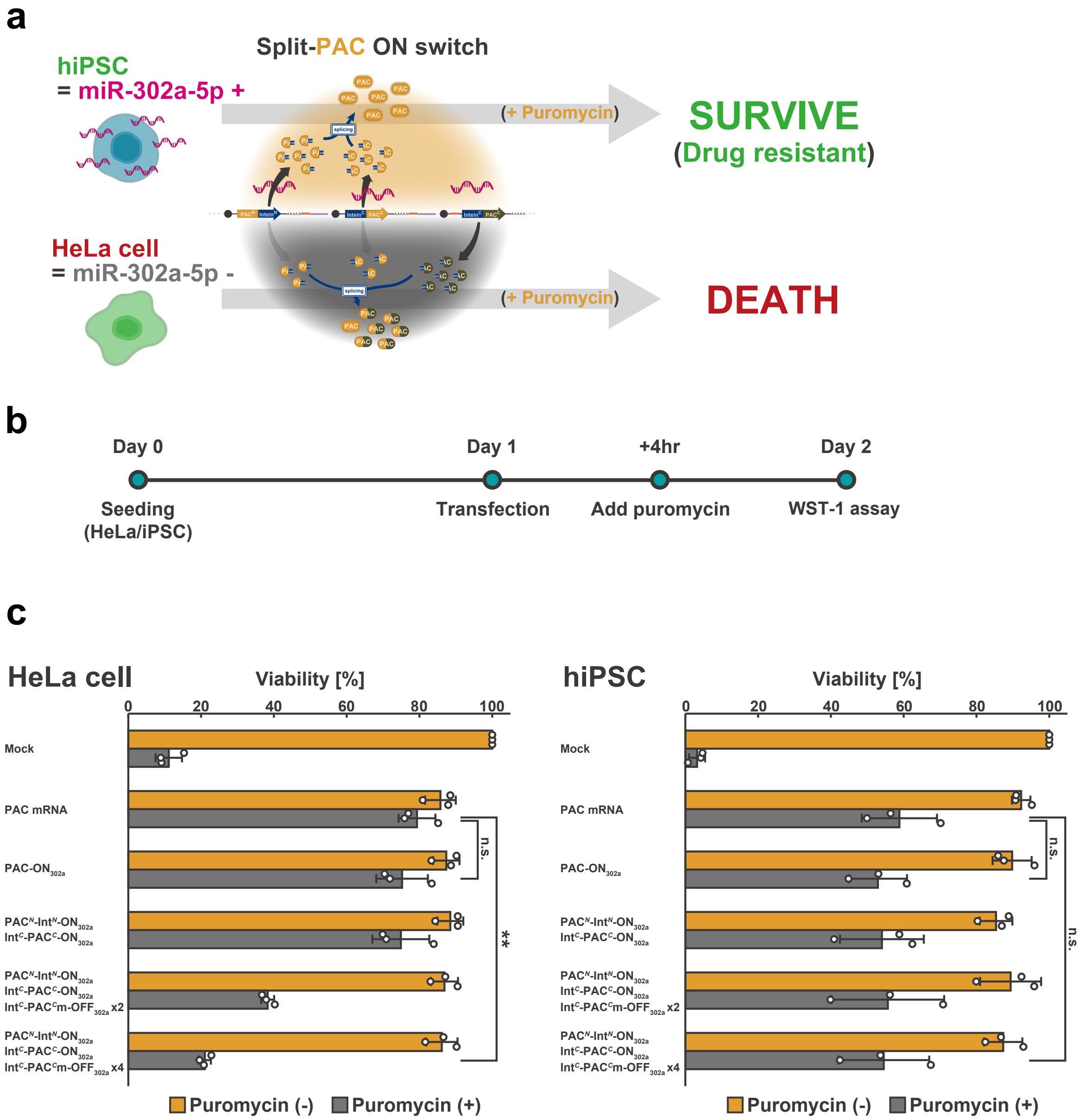

Supplementary Figure 6: miR-302a-5p responsive split-PAC ON switch system in hiPSCs and HeLa cells.

a, Schematic illustration of miR-302a-5p-responsive split ON switch system regulating puromycin N-acetyltransferase (PAC). This system suppresses the leaky activity of PAC in miR-302a-5p negative cells (HeLa cells) while maintaining the drug-resistance in miR-302a-5p positive cells (hiPSCs).

b, Schematic of the time course of this experiment.

c, Viability of HeLa cells and hiPSCs in the WST-1 assay. The viability of each cell line was determined by dividing the values of each condition by those of the mock control without puromycin. Error bars represent means ± SD (n=3), and data of each biological replicate are

shown as a point. Statistical analysis by two-sided Welch's *t*-test, $^{**}P<0.01$, n.s.: not significant ($P>0.05$). Each *P*-value is listed in Supplementary Table 4. Source data are provided as a Source Data file.

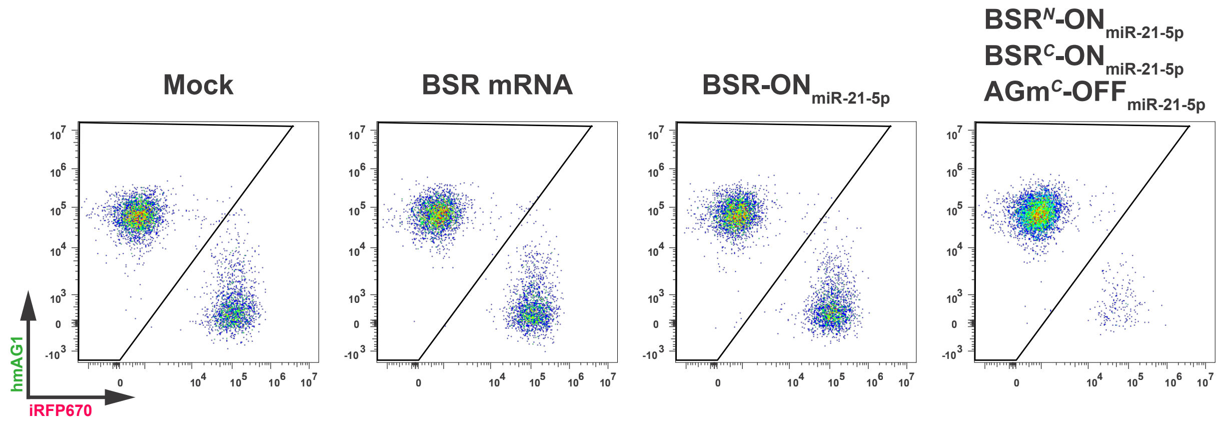

Supplementary Figure 7: Scatter plots of cells after purification by the split-BSR switch system.

Representative 2D flow cytometry plots. The horizontal axis shows the fluorescence intensity of iRFP670, and the vertical axis shows the fluorescence intensity of hmAG1.

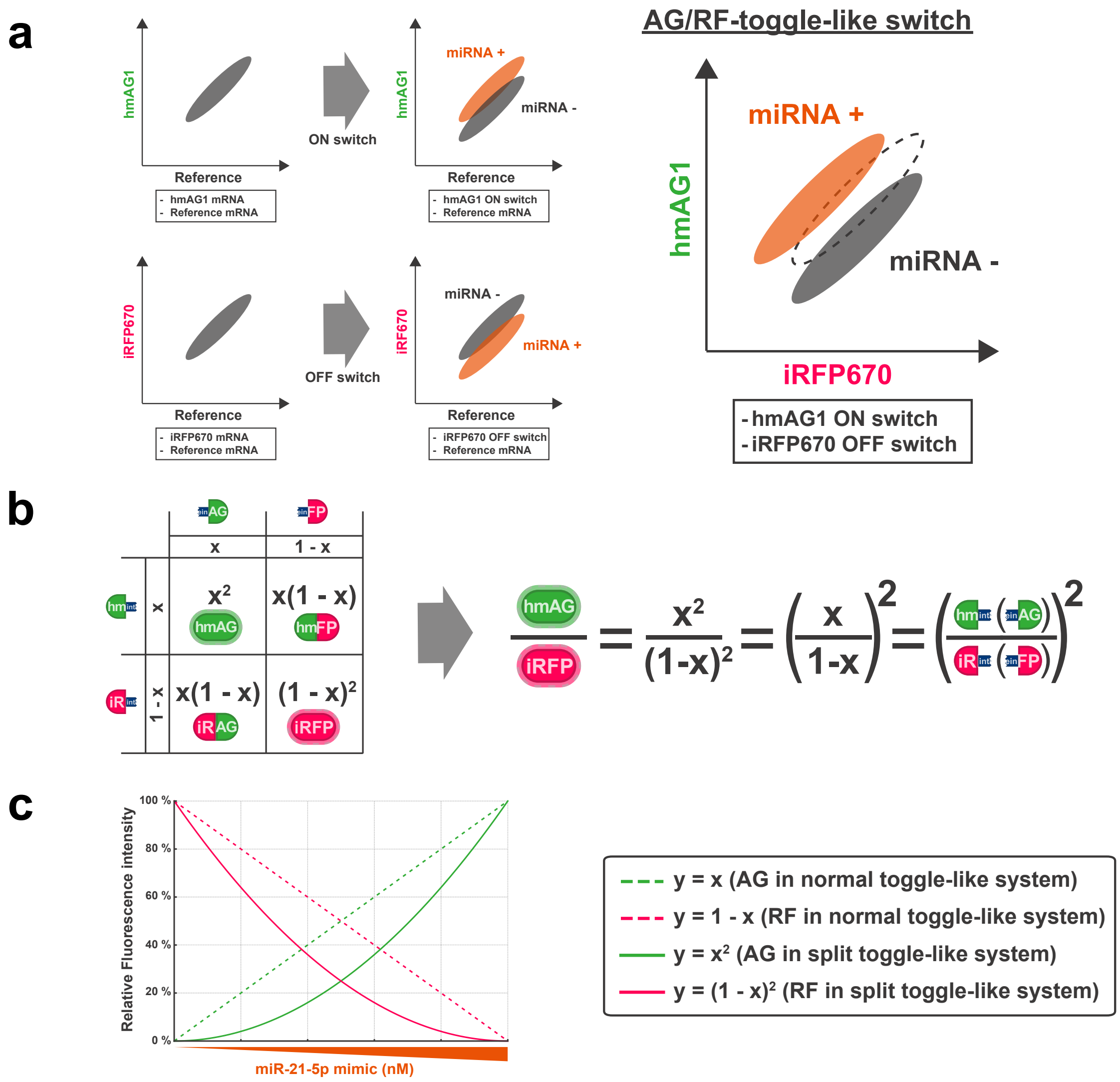

Supplementary Figure 8: Split toggle-like system shows improved binary behavior.

a, A toggle-like switch system, which switches between two fluorescences in response to target miRNA activity, can be implemented by introducing ON and OFF switches, each coding different fluorescence proteins (hmAG1 and iRFP670 in this schematic explanation). A single RNA switch often fails to completely separate two cell populations mainly due to a low ON/OFF ratio or small differences in miRNA activity. The toggle-like system enables clearer separation of two cell populations on flow cytometry 2D plots compared to the case of using a single RNA switch.

b, The expected relationship between the intracellular amount of protein fragments and the intensity of two fluorescence under the assumption that the protein splicing of N-intein and C-intein occurs randomly irrespective of the franking extein sequences with 100% splicing efficiency.

In this illustration, translation amount from the ON switch coding for hmAG1 fragments is set as x, and translation amount from the OFF switch coding for iRFP670 fragments is set as 1-x. In the case of the "normal toggle-like system," which uses an ON switch coding full-length hmAG1 and an OFF switch coding full-length iRFP670, it is predicted that the final observed ratio of green to red fluorescence intensity will be the same as the translation ratio from the ON and OFF switches, which is x:1-x. On the other hand, in the "split toggle-like system," which uses split ON switches coding hmAG1 fragments and split OFF switches coding iRFP670 fragments, the ratio of the full-length protein presence will be $x^2:(1-x)^2$ as shown in this figure. This means that the fluorescence intensity ratio is the square of the actual translation ratio, allowing for a more sensitive switch between the two fluorescence in response to miRNA activity.

c, The expected relationship between miRNA concentration and the normalized intensities of hmAG1 and iRFP670 under the assumption, in addition to the assumption mentioned in **b**, that the AG-ON and RF-OFF switches modulate the translation of downstream genes in proportion to the miRNA concentration and that the translation output from these switches reach 100% and 0% respectively at a certain threshold value.

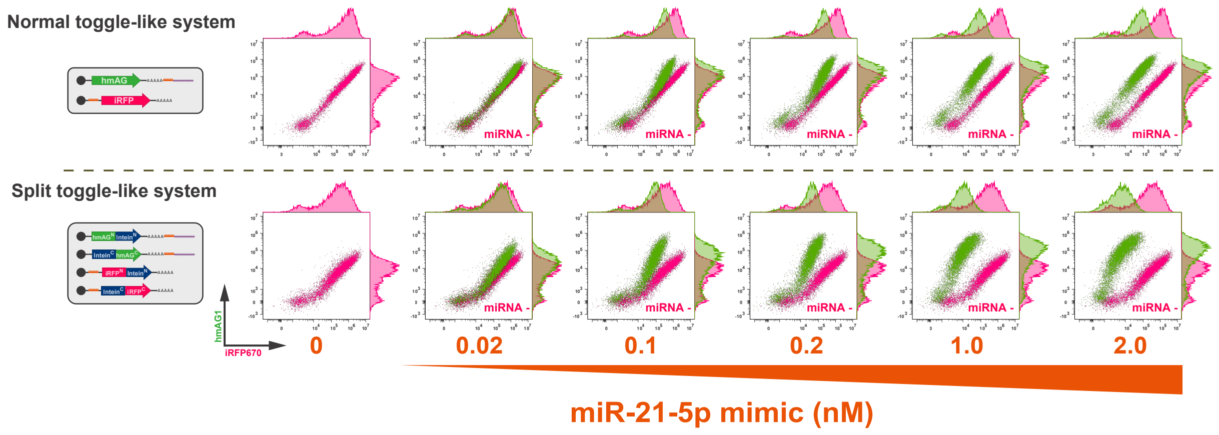

Supplementary Figure 9: Scatter plots of cells introduced with normal or split toggle-like systems.

Representative 2D flow cytometry plots. The horizontal axis shows the fluorescence intensity of iRFP670, and the vertical axis shows the fluorescence intensity of hmAG1. The plots for the cell populations without miR-21-5p mimic (far left, magenta) are shown alongside the results of each condition, with varying concentrations of miR-21-5p mimic (green).

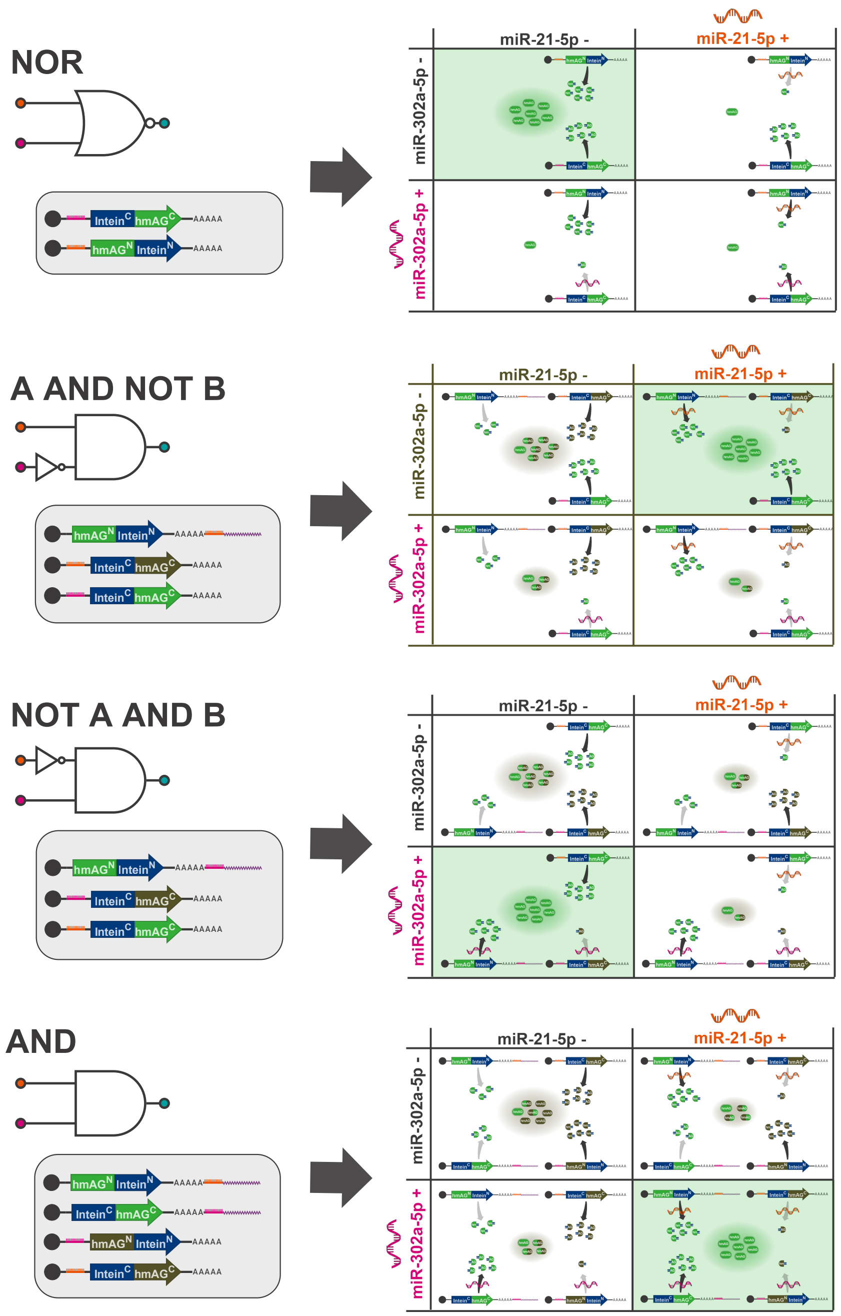

IN		OUT
miR-21	miR-302	hmAG
0	0	1
1	0	0
0	1	0
1	1	0

IN		OUT
miR-21	miR-302	hmAG
0	0	0
1	0	1
0	1	0
1	1	0

IN		OUT
miR-21	miR-302	hmAG
0	0	0
1	0	0
0	1	1
1	1	0

IN		OUT
miR-21	miR-302	hmAG
0	0	0
1	0	0
0	1	0
1	1	1

Supplementary Figure 10: Mechanism of four types of two-input systems.

Schematic illustrations of the expected behaviors of each RNA switch set in transfected cells. Introducing an ON switch along with a complementary OFF switch, coding the opposing inactivated fragment but sharing the same miRNA target site, allows for the suppression of the logic gate leakage.

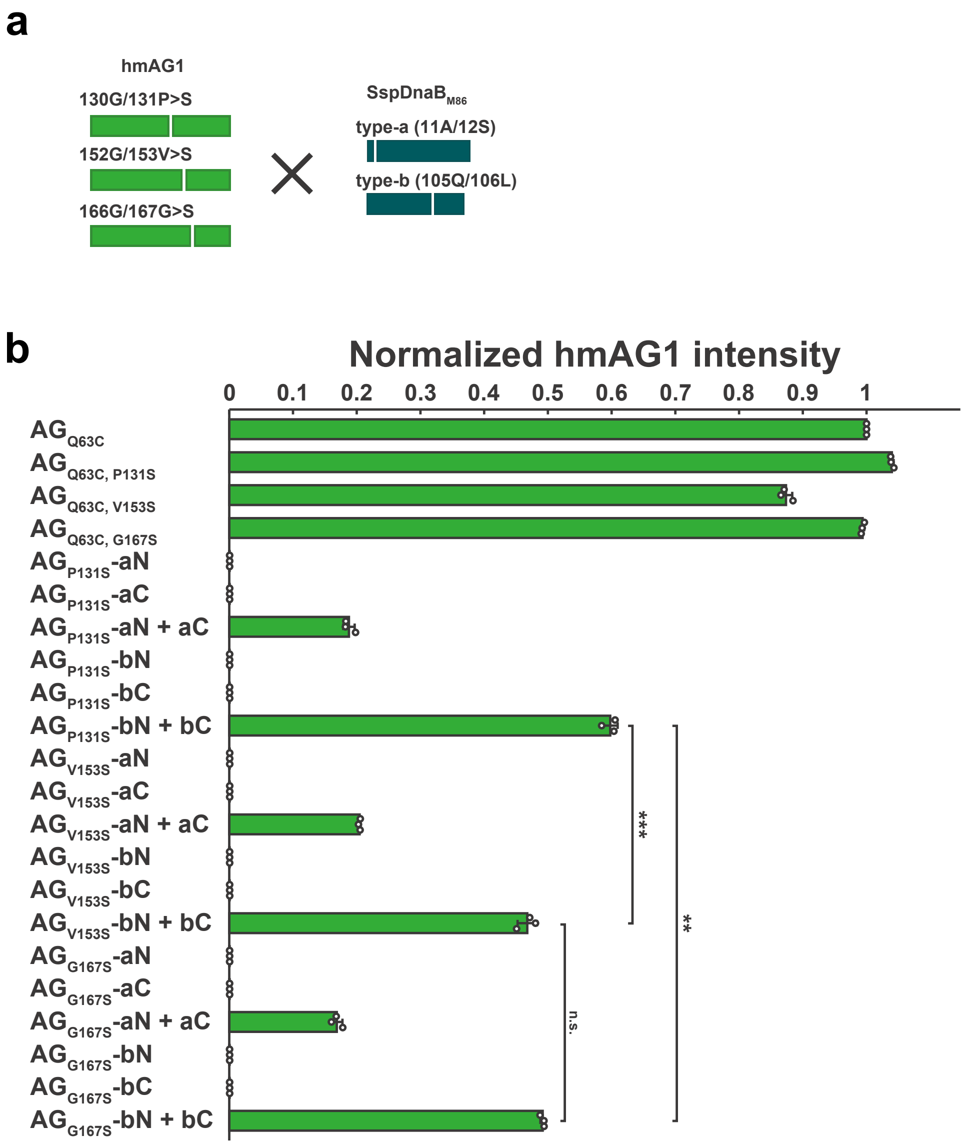

Supplementary Figure 11: Evaluation of split sites of hmAG1 and SspDnaB intein for the construction of three-input logic circuits.

a, In this study, to construct miRNA-responsive three-input logic circuits using hmAG1 as the output protein, we explored (i) a second split site within hmAG1 in addition to the first split site (62F/63Q) and (ii) a split site within the split-intein, SspDnaB$_{M86}$, which is known to be orthogonal to NpuDnaE. For (i), we evaluated three split patterns: 130G/131P, 152G/153V, and 166G/167G, while for (ii), we assessed two split patterns: 11A/12S ("type-a") and 105Q/106L ("type-b"). To evaluate all possible six combinations, we designed a total of 12 fragmented variants and measured the fluorescence activity when introducing either the N- or C-terminal fragments alone, or both fragments together, in HEK293FT cells.

b, Normalized fluorescence intensity in HEK293FT cells transfected with mRNA coding full-length or fragmented hmAG1 variants. Error bars represent means ± SD (n=3), and data of each biological replicate are shown as a point. Statistical analysis by two-sided Welch's *t*-test, $^{**}P<0.01$, $^{***}P<0.001$, n.s.: not significant ($P>0.05$). Each *P*-value is listed in Supplementary Table 4. Source data are provided as a Source Data file.

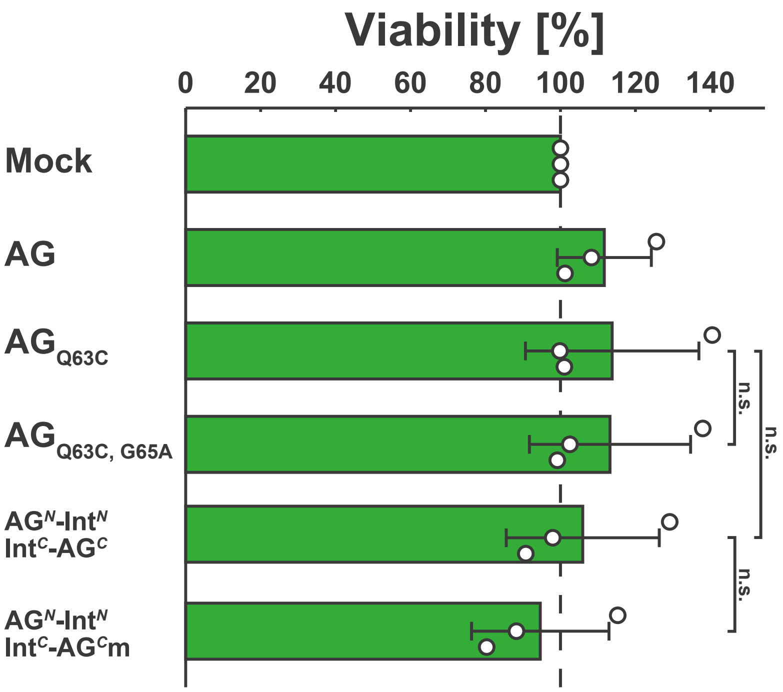

Supplementary Figure 12: Evaluation of cytotoxicity of split-intein and mutated protein fragments in HEK293FT cell.

Viability of HeLa cells transfected with each set of mRNAs in the CellTiter-Glo Luminescent Cell Viability Assay. The viability of each cell line was determined by dividing the values of each condition by those of the mock control. Error bars represent means ± SD (n=3), and data of each biological replicate are shown as a point. Statistical analysis by two-sided Welch's *t*-test, n.s.: not significant (P>0.05). Each P-value is listed in Supplementary Table 4. Source data are provided as a Source Data file.

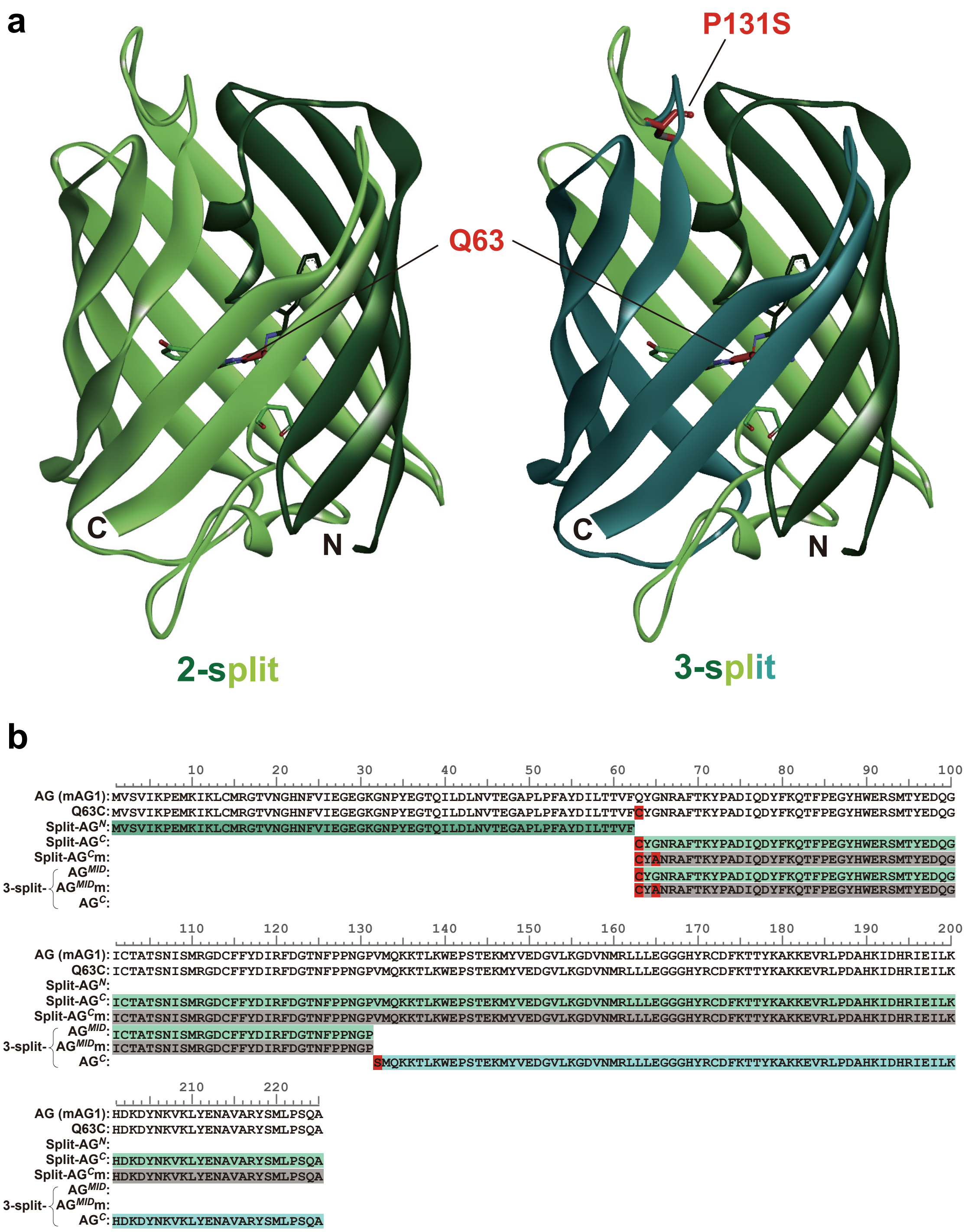

Supplementary Figure 13: Split points for monomeric AG.

a, Three-dimensional structure of AG (mAG, mAG1: monomeric Azami-Green (1); Protein Data Bank ID: 3adf). Dark green: split-AG^{N} (common for two-split (left) and three-split (right)), light

green: split-AG^{C} for two-split (left) and AG^{MID} for three-split (right), blue green: AG^{MID} for three-split (right).

b, Amino acid sequences of the original AG, Q63C mutant, split-AG^{N}, split-AG^{C}, split-AG^{C}m (leak-canceler), three-split-AG^{MID}, three-split-AG^{MID}m (leak canceler in three-split experiments), and three-split-AG^{C}. Q63C mutant corresponds to intein-spliced mature protein, which has the cysteine for the intein-splicing. Splitting position was designed referring to another fluorescent protein, GFP[5]. In split-AG^{C}m, the G65A mutant, corresponding to G67A in GFP, was used as a leak-canceller to produce a non-functional fragment to inhibit the formation of a functional fluorophore[6].

a

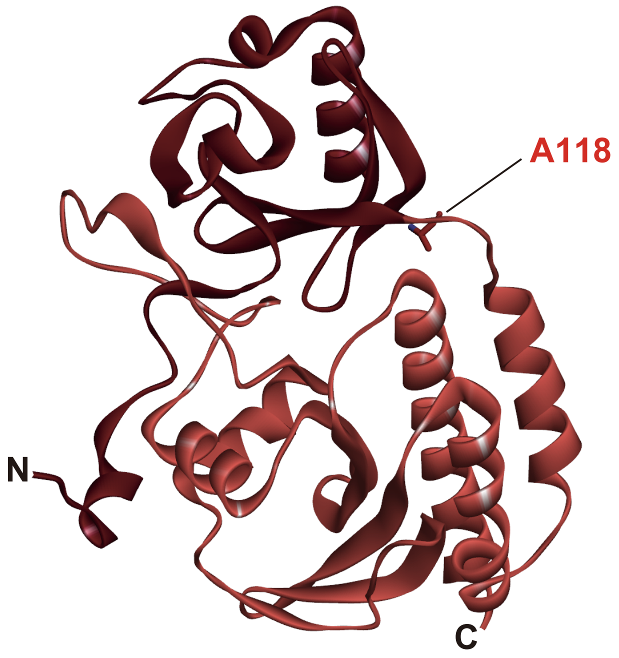

b

	1–100
iRFP670:	MARKVDLTSCDREPIHIPGSIQPCGCLLACDAQAVRITRITENAGAFFGRETPRVGELLADYFGETEAHALRNALAQSSDPKRPALIFGWRDGLTGRTFD
A118C:	MARKVDLTSCDREPIHIPGSIQPCGCLLACDAQAVRITRITENAGAFFGRETPRVGELLADYFGETEAHALRNALAQSSDPKRPALIFGWRDGLTGRTFD
Split-iRFP^N:	MARKVDLTSCDREPIHIPGSIQPCGCLLACDAQAVRITRITENAGAFFGRETPRVGELLADYFGETEAHALRNALAQSSDPKRPALIFGWRDGLTGRTFD
Split-iRFP^C:	

	101–200
iRFP670:	ISLHRHDGTSIIEFEPAAAEQADNPLRLTRQIIARTKELKSLEEMAARVPRYLQAMLGYHRVMLYRFADDGSGMVIGEAKRSDLESFLGQHFPASLVPQQ
A118C:	ISLHRHDGTSIIEFEPACAEQADNPLRLTRQIIARTKELKSLEEMAARVPRYLQAMLGYHRVMLYRFADDGSGMVIGEAKRSDLESFLGQHFPASLVPQQ
Split-iRFP^N:	ISLHRHDGTSIIEFEPA
Split-iRFP^C:	CAEQADNPLRLTRQIIARTKELKSLEEMAARVPRYLQAMLGYHRVMLYRFADDGSGMVIGEAKRSDLESFLGQHFPASLVPQQ

	201–300
iRFP670:	ARLLYLKNAIRVVSDSRGISSRIVPEHDASGAALDLSFAHLRSISPCHLEFLRNMGVSASMSLSIIIDGTLWGLIICHHYEPRAVPMAQRVAAEMFADFL
A118C:	ARLLYLKNAIRVVSDSRGISSRIVPEHDASGAALDLSFAHLRSISPCHLEFLRNMGVSASMSLSIIIDGTLWGLIICHHYEPRAVPMAQRVAAEMFADFL
Split-iRFP^N:	
Split-iRFP^C:	ARLLYLKNAIRVVSDSRGISSRIVPEHDASGAALDLSFAHLRSISPCHLEFLRNMGVSASMSLSIIIDGTLWGLIICHHYEPRAVPMAQRVAAEMFADFL

	301–311
iRFP670:	SLHFTAAHHQR
A118C:	SLHFTAAHHQR
Split-iRFP^N:	
Split-iRFP^C:	SLHFTAAHHQR

Supplementary Figure 14: Split points for RF.

a, Three-dimensional structure of RF (iRFP670) predicted by AlphaFold2. Dark red: split-iRFP^N, light red: split-iRFP^C.

b, Amino acid sequences of the original iRFP670, A118C mutant, split-iRFP^N, and split-iRFP^C. A118C mutant corresponds to intein-spliced mature protein, which has the cysteine for intein-splicing. The splitting position was designed within the loop between PAS and GAF domain[7].

a

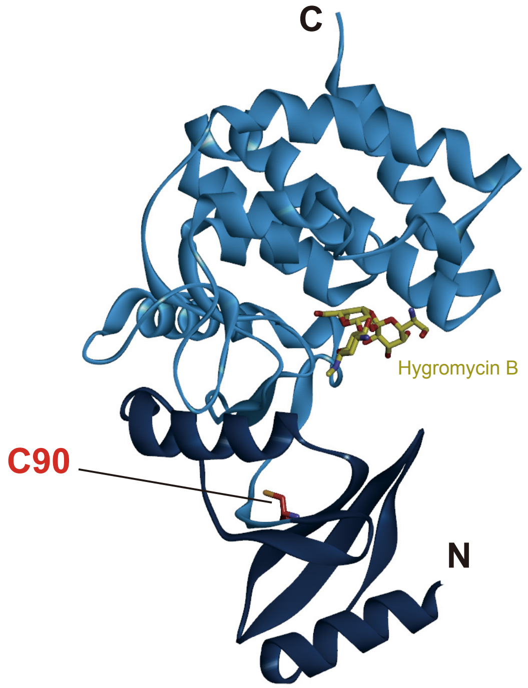

b

HPH: MKKPELTATSVEKFLIEKFDSVSDLMQLSEGEESRAFSFDVGGRGYVLRVNSCADGFYKDRYVYRHFASAALPIPEVLDIGEFSESLTYCISRRAQGVTL
Split-HPH^N: MKKPELTATSVEKFLIEKFDSVSDLMQLSEGEESRAFSFDVGGRGYVLRVNSCADGFYKDRYVYRHFASAALPIPEVLDIGEFSESLTY
Split-HPH^C: CISRRAQGVTL

HPH: QDLPETELPAVLQPVAEAMDAIAAADLSQTSGFGPFGPQGIGQYTTWRDFICAIADPHVYHWQTVMDDTVSASVAQALDELMLWAEDCPEVRHLVHADFG
Split-HPH^N:
Split-HPH^C: QDLPETELPAVLQPVAEAMDAIAAADLSQTSGFGPFGPQGIGQYTTWRDFICAIADPHVYHWQTVMDDTVSASVAQALDELMLWAEDCPEVRHLVHADFG

HPH: SNNVLTDNGRITAVIDWSEAMFGDSQYEVANIFFWRPWLACMEQQTRYFERRHPELAGSPRLRAYMLRIGLDQLYQSLVDGNFDDAAWAQGRCDAIVRSG
Split-HPH^N:
Split-HPH^C: SNNVLTDNGRITAVIDWSEAMFGDSQYEVANIFFWRPWLACMEQQTRYFERRHPELAGSPRLRAYMLRIGLDQLYQSLVDGNFDDAAWAQGRCDAIVRSG

HPH: AGTVGRTQIARRSAAVWTDGCVEVLADSGNRRPSTRPRAKE
Split-HPH^N:
Split-HPH^C: AGTVGRTQIARRSAAVWTDGCVEVLADSGNRRPSTRPRAKE

Supplementary Figure 15: Split points for HPH.

a, Three-dimensional structure of HPH (Hygromycin-B 4-O-kinase; Protein Data Bank ID: 3tyk). Dark blue: split-HPH^N, light blue: split-HPH^C.

b, Amino acid sequences of the original HPH (derived from Escherichia coli Uniprot: P00557), split-HPH^N, and split-HPH^C. Splitting position was designed by referring to ref[3].

a

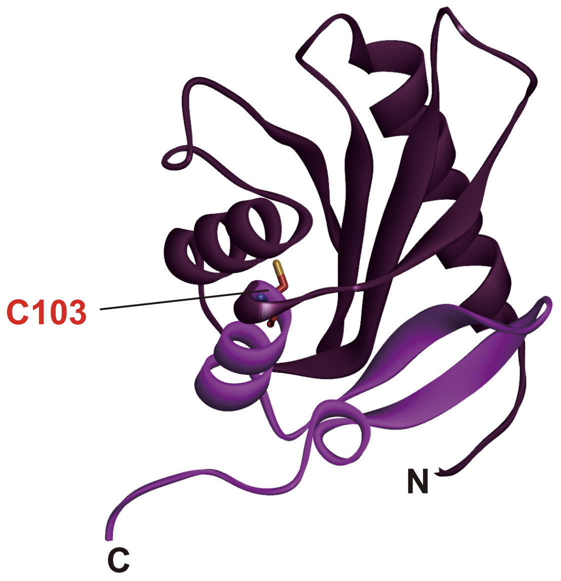

b

```
                 10        20        30        40        50        60        70        80        90       100
BSR:         MKTFNISQQDLELVEVATEKITMLYEDNKHHVGAAIRTKTGEIISAVHIEAYIGRVTVCAEAIAIGSAVSNGQKDFDTIVAVRHPYSDEVDRSIRVVSPC
Split-BSR^N: MKTFNISQQDLELVEVATEKITMLYEDNKHHVGAAIRTKTGEIISAVHIEAYIGRVTVCAEAIAIGSAVSNGQKDFDTIVAVRHPYSDEVDRSIRVVSPC
Split-BSR^C:

                110       120       130       140
BSR:         GMCRELISDYAPDCFVLIEMNGKLVKTTIEELIPLKYTRN
Split-BSR^N: GM
Split-BSR^C:   CRELISDYAPDCFVLIEMNGKLVKTTIEELIPLKYTRN
```

Supplementary Figure 16: Split points for BSR.

a, Three-dimensional structure of BSR (Blasticidin-S deaminase) predicted by AlphaFold (AlphaFold Protein Structure Database: AF-P33967-F1-model_v4). Dark purple: split-BSR^{N}, light purple: split-BSR^{C}.

b, Amino acid sequences of the original BSR (derived from Bacillus cereus; UniProt: P33967), split-BSR^{N}, and split-BSR^{C}. Splitting position was designed by referring to ref[3].

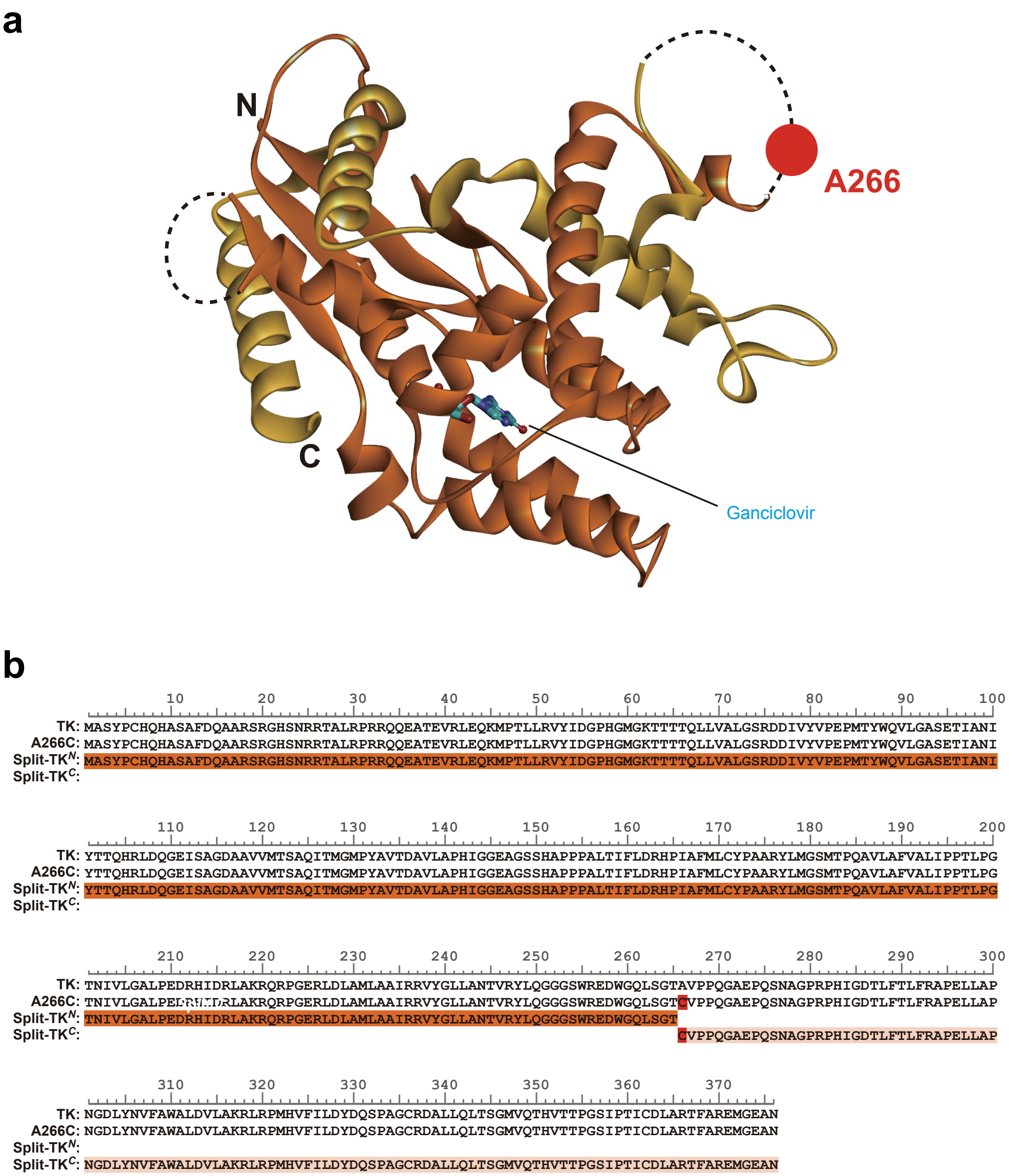

Supplementary Figure 17: Split points for TK.

a, Three-dimensional structure of TK (Thymidine kinase, Protein Data Bank ID: 1ki2). Dark orange: split-TK^N, light orange: split-TK^C.

b, Amino acid sequences of the original TK (derived from HSV: Human herpes simplex virus 1, Gene ID: 24271467), split-TK^N, split-TK^C. Splitting position was designed by referring to ref[4].

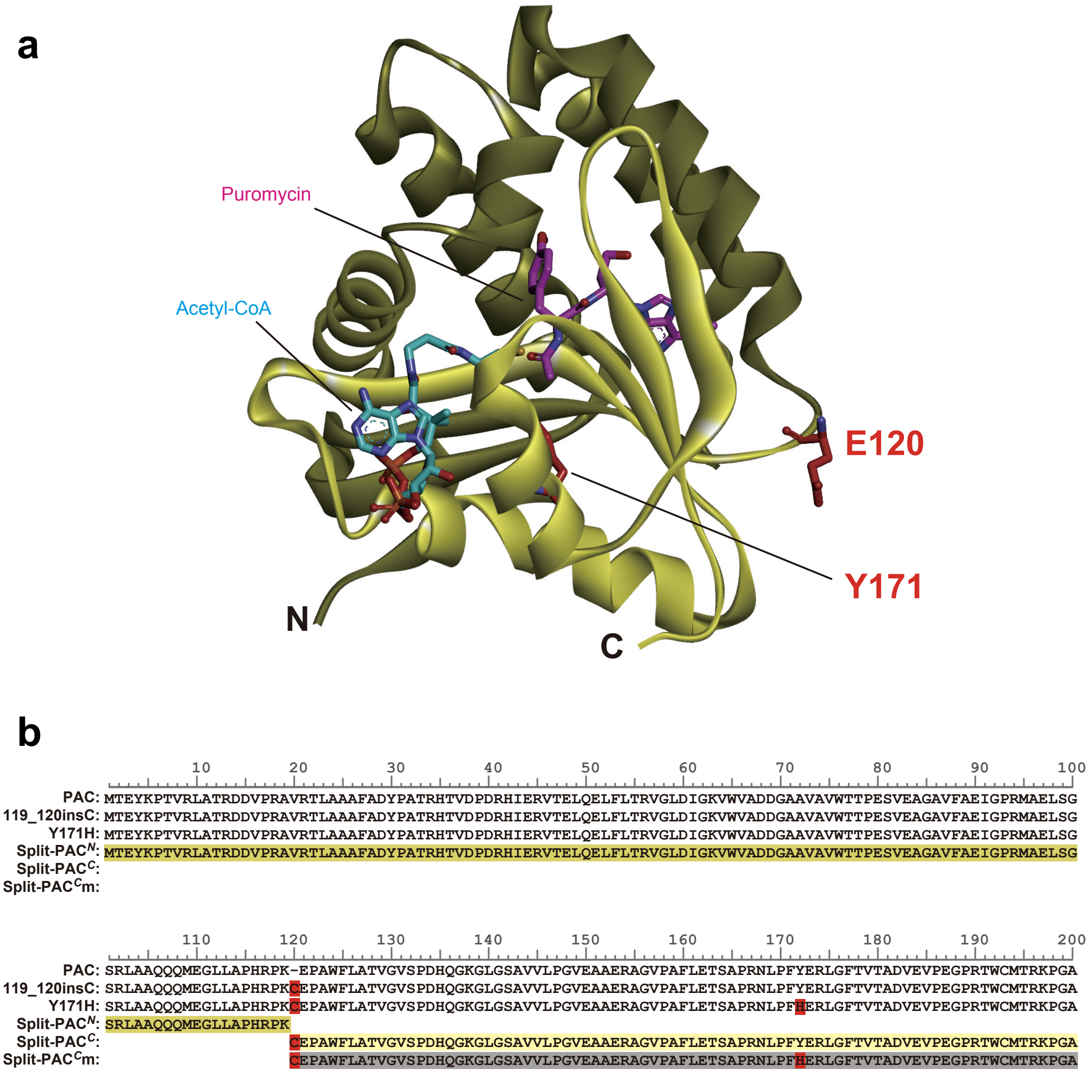

Supplementary Figure 18: Split points for PAC.

a, Three-dimensional structure of PAC (Puromycin-N-acetyltransferase; Protein Data Bank ID: 7k0a). Dark yellow: split-PAC^{N}, light yellow: split-PAC^{C}.

b, Amino acid sequences of the original PAC (derived from Streptomyces alboniger, UniProt AC: P13249), 119_120insC mutant, Y171H mutant, split-PAC^{N}, split-PAC^{C}, and split-PAC^{C}m. 119_120insC mutant corresponds to intein-spliced mature protein, which has a cysteine insertion between K119 and E120. Y171H is a defective mutation[8], used in split-PAC^{C}m as a leak-canceller to produce a non-function PAC protein. Splitting position was designed by referring to ref[3].

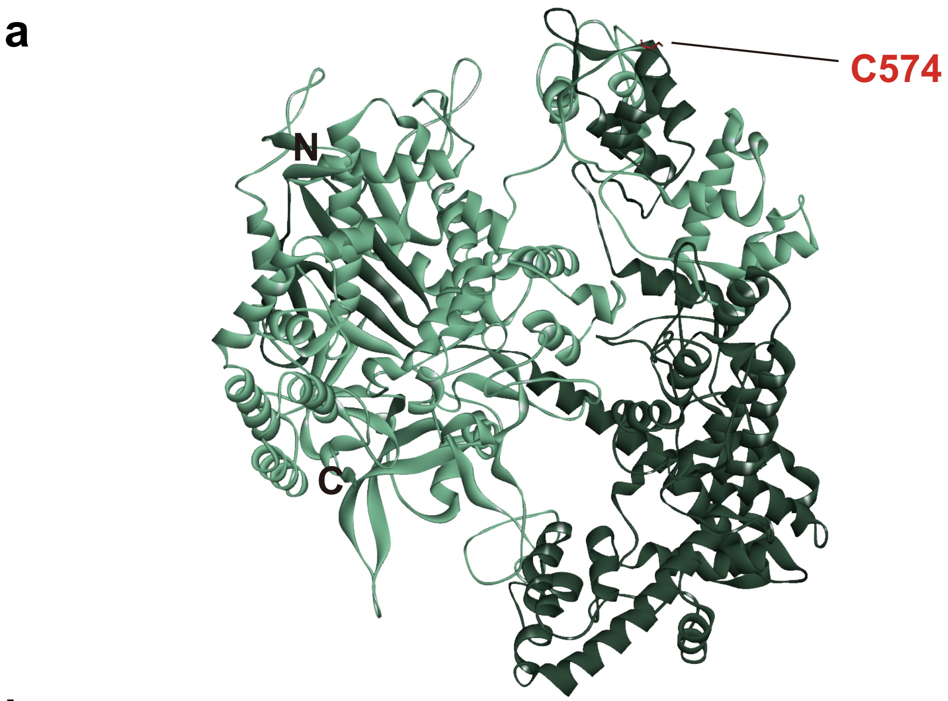

b

```
          10        20        30        40        50        60        70        80        90       100       110       120
Cas9  : MDKKYSIGLDIGTNSVGWAVITDEYKVPSKKFKVLGNTDRHSIKKNLIGALLFDSGETAEATRLKRTARRRYTRRKNRICYLQEIFSNEMAKVDDSFFHRLEESFLVEEDKKHERHPIFG
Cas9N : MDKKYSIGLDIGTNSVGWAVITDEYKVPSKKFKVLGNTDRHSIKKNLIGALLFDSGETAEATRLKRTARRRYTRRKNRICYLQEIFSNEMAKVDDSFFHRLEESFLVEEDKKHERHPIFG
Cas9C :

         130       140       150       160       170       180       190       200       210       220       230       240
Cas9  : NIVDEVAYHEKYPTIYHLRKKLVDSTDKADLRLIYLALAHMIKFRGHFLIEGDLNPDNSDVDKLFIQLVQTYNQLFEENPINASGVDAKAILSARLSKSRRLENLIAQLPGEKKNGLFGN
Cas9N : NIVDEVAYHEKYPTIYHLRKKLVDSTDKADLRLIYLALAHMIKFRGHFLIEGDLNPDNSDVDKLFIQLVQTYNQLFEENPINASGVDAKAILSARLSKSRRLENLIAQLPGEKKNGLFGN
Cas9C :

         250       260       270       280       290       300       310       320       330       340       350       360
Cas9  : LIALSLGLTPNFKSNFDLAEDAKLQLSKDTYDDDLDNLLAQIGDQYADLFLAAKNLSDAILLSDILRVNTEITKAPLSASMIKRYDEHHQDLTLLKALVRQQLPEKYKEIFFDQSKNGYA
Cas9N : LIALSLGLTPNFKSNFDLAEDAKLQLSKDTYDDDLDNLLAQIGDQYADLFLAAKNLSDAILLSDILRVNTEITKAPLSASMIKRYDEHHQDLTLLKALVRQQLPEKYKEIFFDQSKNGYA
Cas9C :

         370       380       390       400       410       420       430       440       450       460       470       480
Cas9  : GYIDGGASQEEFYKFIKPILEKMDGTEELLVKLNREDLLRKQRTFDNGSIPHQIHLGELHAILRRQEDFYPFLKDNREKIEKILTFRIPYYVGPLARGNSRFAWMTRKSEETITPWNFEE
Cas9N : GYIDGGASQE[illegible]KPILEKMDGTEELLVKLNREDLLRKQRTFDNGSIPHQIHLGELHAILRRQEDFYPFLKDNREKIEKILTFRIPYYVGPLARGNSRFAWMTRKSEETITPWNFEE
Cas9C :
```

Split C

```
         490       500       510       520       530       540       550       560       570       580       590       600
Cas9  : VVDKGASAQSFIERMTNFDKNLPNEKVLPKHSLLYEYFTVYNELTKVKYVTEGMRKPAFLSGEQKKAIVDLLFKTNRKVTVKQLKEDYFKKIECFDSVEISGVEDRFNASLGTYHDLLKI
Cas9N : VVDKGASAQSFIERMTNFDKNLPNEKVLPKHSLLYEYFTVYNELTKVKYVTEGMRKPAFLSGEQKKAIVDLLFKTNRKVTVKQLKEDYFKKIE
Cas9C :                                                                                              CFDSVEISGVEDRFNASLGTYHDLLKI

         610       620       630       640       650       660       670       680       690       700       710       720
Cas9  : IKDKDFLDNEENEDILEDIVLTLTLFEDREMIEERLKTYAHLFDDKVMKQLKRRRYTGWGRLSRKLINGIRDKQSGKTILDFLKSDGFANRNFMQLIHDDSLTFKEDIQKAQVSGQGDSL
Cas9N :
Cas9C : IKDKDFLDNEENEDILEDIVLTLTLFEDREMIEERLKTYAHLFDDKVMKQLKRRRYTGWGRLSRKLINGIRDKQSGKTILDFLKSDGFANRNFMQLIHDDSLTFKEDIQKAQVSGQGDSL

         730       740       750       760       770       780       790       800       810       820       830       840
Cas9  : HEHIANLAGSPAIKKGILQTVKVVDELVKVMGRHKPENIVIEMARENQTTQKGQKNSRERMKRIEEGIKELGSQILKEHPVENTQLQNEKLYLYYLQNGRDMYVDQELDINRLSDYDVDH
Cas9N :
Cas9C : HEHIANLAGSPAIKKGILQTVKVVDELVKVMGRHKPENIVIEMARENQTTQKGQKNSRERMKRIEEGIKELGSQILKEHPVENTQLQNEKLYLYYLQNGRDMYVDQELDINRLSDYDVDH

         850       860       870       880       890       900       910       920       930       940       950       960
Cas9  : IVPQSFLKDDSIDNKVLTRSDKNRGKSDNVPSEEVVKKMKNYWRQLLNAKLITQRKFDNLTKAERGGLSELDKAGFIKRQLVETRQITKHVAQILDSRMNTKYDENDKLIREVKVITLKS
Cas9N :
Cas9C : IVPQSFLKDDSIDNKVLTRSDKNRGKSDNVPSEEVVKKMKNYWRQLLNAKLITQRKFDNLTKAERGGLSELDKAGFIKRQLVETRQITKHVAQILDSRMNTKYDENDKLIREVKVITLKS

         970       980       990      1000      1010      1020      1030      1040      1050      1060      1070      1080
Cas9  : KLVSDFRKDFQFYKVREINNYHHAHDAYLNAVVGTALIKKYPKLESEFVYGDYKVYDVRKMIAKSEQEIGKATAKYFFYSNIMNFFKTEITLANGEIRKRPLIETNGETGEIVWDKGRDF
Cas9N :
Cas9C : KLVSDFRKDFQFYKVREINNYHHAHDAYLNAVVGTALIKKYPKLESEFVYGDYKVYDVRKMIAKSEQEIGKATAKYFFYSNIMNFFKTEITLANGEIRKRPLIETNGETGEIVWDKGRDF

        1090      1100      1110      1120      1130      1140      1150      1160      1170      1180      1190      1200
Cas9  : ATVRKVLSMPQVNIVKKTEVQTGGFSKESILPKRNSDKLIARKKDWDPKKYGGFDSPTVAYSVLVVAKVEKGKSKKLKSVKELLGITIMERSSFEKNPIDFLEAKGYKEVKKDLIIKLPK
Cas9N :
Cas9C : ATVRKVLSMPQVNIVKKTEVQTGGFSKESILPKRNSDKLIARKKDWDPKKYGGFDSPTVAYSVLVVAKVEKGKSKKLKSVKELLGITIMERSSFEKNPIDFLEAKGYKEVKKDLIIKLPK

        1210      1220      1230      1240      1250      1260      1270      1280      1290      1300      1310      1320
Cas9  : YSLFELENGRKRMLASAGELQKGNELALPSKYVNFLYLASHYEKLKGSPEDNEQKQLFVEQHKHYLDEIIEQISEFSKRVILADANLDKVLSAYNKHRDKPIREQAENIIHLFTLTNLGA
Cas9N :
Cas9C : YSLFELENGRKRMLASAGELQKGNELALPSKYVNFLYLASHYEKLKGSPEDNEQKQLFVEQHKHYLDEIIEQISEFSKRVILADANLDKVLSAYNKHRDKPIREQAENIIHLFTLTNLGA

        1330      1340      1350      1360
Cas9  : PAAFKYFDTTIDRKRYTSTKEVLDATLIHQSITGLYETRIDLSQLGGD
Cas9N :
Cas9C : PAAFKYFDTTIDRKRYTSTKEVLDATLIHQSITGLYETRIDLSQLGGD
```

Supplementary Figure 19: Split points for *sp*Cas9.

a, Three-dimensional structure of Cas9 (*Streptococcus pyogenes* Cas9, Protein Data Bank ID: 5f9r). Dark green: split-Cas9^{N}, light green: split-Cas9^{C}.

b, Amino acid sequences of original Cas9, split-Cas9^{N}, split-Cas9^{C}. Splitting position was designed referring to ref[9].

Supplementary Table 1

Nucleotide sequence used in this study.

Name	Sequence	Length (nt)	Length (aa)
**ORF**			
**hmAG1**	AUGGUGAGCGUGAUCAAGCCCGAGAUGAAGAUCAAGCUGUGCAUGAGGGGCACCGUGAACGGCCACAACUUCGUGAUCGAGGGCGAGGGCAAGGG<br>CAACCCCUACGAGGGCACCCAGAUCCUGGACCUGAACGUGACCGAGGGCGCCCCCCUGCCCUUCGCCUACGACAUCCUGACCACCGUGUUCCAGU<br>ACGGCAACAGGGCCUUCACCAAGUACCCCGCCGACAUCCAGGACUACUUCAAGCAGACCUUCCCCGAGGGCUACCACUGGGAGAGGAGCAUGACC<br>UACGAGGACCAGGGCAUCUGCACCGCCACCAGCAACAUCAGCAUGAGGGGCGACUGCUUCUUCUACGACAUCAGGUUCGACGGCACCAACUUCCC<br>CCCCAACGGCCCCGUGAUGCAGAAGAAGACCCUGAAGUGGGAGCCCAGCACCGAGAAGAUGUACGUGGAGGACGGCGUGCUGAAGGGCGACGUGA<br>ACAUGAGGCUGCUGCUGGAGGGCGGCGGCCACUACAGGUGCGACUUCAAGACCACCUACAAGGCCAAGAAGGAGGUGAGGCUGCCCGACGCCCAC<br>AAGAUCGACCACAGGAUCGAGAUCCUGAAGCACGACAAGGACUACAACAAGGUGAAGCUGUACGAGAACGCCGUGGCCAGGUACUCCAUGCUGCC<br>CAGCCAGGCCAAGuga	681	227
**hmAG1$_{Q63C}$**	AUGGUGAGCGUGAUCAAGCCCGAGAUGAAGAUCAAGCUGUGCAUGAGGGGCACCGUGAACGGCCACAACUUCGUGAUCGAGGGCGAGGGCAAGGG<br>CAACCCCUACGAGGGCACCCAGAUCCUGGACCUGAACGUGACCGAGGGCGCCCCCCUGCCCUUCGCCUACGACAUCCUGACCACCGUGUUCUGCU<br>ACGGCAACAGGGCCUUCACCAAGUACCCCGCCGACAUCCAGGACUACUUCAAGCAGACCUUCCCCGAGGGCUACCACUGGGAGAGGAGCAUGACC<br>UACGAGGACCAGGGCAUCUGCACCGCCACCAGCAACAUCAGCAUGAGGGGCGACUGCUUCUUCUACGACAUCAGGUUCGACGGCACCAACUUCCC<br>CCCCAACGGCCCCGUGAUGCAGAAGAAGACCCUGAAGUGGGAGCCCAGCACCGAGAAGAUGUACGUGGAGGACGGCGUGCUGAAGGGCGACGUGA<br>ACAUGAGGCUGCUGCUGGAGGGCGGCGGCCACUACAGGUGCGACUUCAAGACCACCUACAAGGCCAAGAAGGAGGUGAGGCUGCCCGACGCCCAC<br>AAGAUCGACCACAGGAUCGAGAUCCUGAAGCACGACAAGGACUACAACAAGGUGAAGCUGUACGAGAACGCCGUGGCCAGGUACUCCAUGCUGCC<br>CAGCCAGGCCAAGuga	681	227
**hmAG1$_{Q63C, G65A}$**	AUGGUGAGCGUGAUCAAGCCCGAGAUGAAGAUCAAGCUGUGCAUGAGGGGCACCGUGAACGGCCACAACUUCGUGAUCGAGGGCGAGGGCAAGGG<br>CAACCCCUACGAGGGCACCCAGAUCCUGGACCUGAACGUGACCGAGGGCGCCCCCCUGCCCUUCGCCUACGACAUCCUGACCACCGUGUUCUGCU<br>ACGCCAACAGGGCCUUCACCAAGUACCCCGCCGACAUCCAGGACUACUUCAAGCAGACCUUCCCCGAGGGCUACCACUGGGAGAGGAGCAUGACC<br>UACGAGGACCAGGGCAUCUGCACCGCCACCAGCAACAUCAGCAUGAGGGGCGACUGCUUCUUCUACGACAUCAGGUUCGACGGCACCAACUUCCC<br>CCCCAACGGCCCCGUGAUGCAGAAGAAGACCCUGAAGUGGGAGCCCAGCACCGAGAAGAUGUACGUGGAGGACGGCGUGCUGAAGGGCGACGUGA<br>ACAUGAGGCUGCUGCUGGAGGGCGGCGGCCACUACAGGUGCGACUUCAAGACCACCUACAAGGCCAAGAAGGAGGUGAGGCUGCCCGACGCCCAC<br>AAGAUCGACCACAGGAUCGAGAUCCUGAAGCACGACAAGGACUACAACAAGGUGAAGCUGUACGAGAACGCCGUGGCCAGGUACUCCAUGCUGCC<br>CAGCCAGGCCAAGuga	681	227
**AG^N-Int^N**	AUGGUGAGCGUGAUCAAGCCCGAGAUGAAGAUCAAGCUGUGCAUGAGGGGCACCGUGAACGGCCACAACUUCGUGAUCGAGGGCGAGGGCAAGGG<br>CAACCCCUACGAGGGCACCCAGAUCCUGGACCUGAACGUGACCGAGGGCGCCCCCCUGCCCUUCGCCUACGACAUCCUGACCACCGUGUUCUGCC<br>UGAGCUACGAGACAGAGAUCCUGACCGUGGAAUACGGCCUGCUGCCUAUCGGCAAGAUCGUGGAAAAGCGGAUCGAGUGCACCGUGUACUCCGUG<br>GAUAACAACGGCAACAUCUACACCCAGCCUGUGGCUCAGUGGCACGACAGAGGCGAGCAAGAGGUGUUCGAGUACUGCCUGGAAGAUGGCAGCCU<br>GAUCAGAGCCACCAAGGACCACAAGUUCAUGACAGUGGACGGCCAGAUGCUGCCCAUCGACGAGAUCUUCGAGCGCGAGCUGGACCUGAUGAGAG<br>UGGACAACCUGCCUAACuga	495	164
**Int^C-AG^C**	AUGAUCAAGAUCGCCACACGGAAGUACCUGGGCAAGCAGAACGUGUACGACAUCGGCGUGGAACGGGACCACAACUUCGCCCUGAAGAACGGCUU<br>UAUCGCCAGCAACUGCUACGGCAACAGGGCCUUCACCAAGUACCCCGCCGACAUCCAGGACUACUUCAAGCAGACCUUCCCCGAGGGCUACCACU<br>GGGAGAGGAGCAUGACCUACGAGGACCAGGGCAUCUGCACCGCCACCAGCAACAUCAGCAUGAGGGGCGACUGCUUCUUCUACGACAUCAGGUUC<br>GACGGCACCAACUUCCCCCCCAACGGCCCCGUGAUGCAGAAGAAGACCCUGAAGUGGGAGCCCAGCACCGAGAAGAUGUACGUGGAGGACGGCGU<br>GCUGAAGGGCGACGUGAACAUGAGGCUGCUGCUGGAGGGCGGCGGCCACUACAGGUGCGACUUCAAGACCACCUACAAGGCCAAGAAGGAGGUGA<br>GGCUGCCCGACGCCCACAAGAUCGACCACAGGAUCGAGAUCCUGAAGCACGACAAGGACUACAACAAGGUGAAGCUGUACGAGAACGCCGUGGCC<br>AGGUACUCCAUGCUGCCCAGCCAGGCCAAGuga	603	200
**Int^C-AG^{C}m**	AUGAUCAAGAUCGCCACACGGAAGUACCUGGGCAAGCAGAACGUGUACGACAUCGGCGUGGAACGGGACCACAACUUCGCCCUGAAGAACGGCUU<br>UAUCGCCAGCAACUGCUACGCCAACAGGGCCUUCACCAAGUACCCCGCCGACAUCCAGGACUACUUCAAGCAGACCUUCCCCGAGGGCUACCACU<br>GGGAGAGGAGCAUGACCUACGAGGACCAGGGCAUCUGCACCGCCACCAGCAACAUCAGCAUGAGGGGCGACUGCUUCUUCUACGACAUCAGGUUC<br>GACGGCACCAACUUCCCCCCCAACGGCCCCGUGAUGCAGAAGAAGACCCUGAAGUGGGAGCCCAGCACCGAGAAGAUGUACGUGGAGGACGGCGU<br>GCUGAAGGGCGACGUGAACAUGAGGCUGCUGCUGGAGGGCGGCGGCCACUACAGGUGCGACUUCAAGACCACCUACAAGGCCAAGAAGGAGGUGA<br>GGCUGCCCGACGCCCACAAGAUCGACCACAGGAUCGAGAUCCUGAAGCACGACAAGGACUACAACAAGGUGAAGCUGUACGAGAACGCCGUGGCC<br>AGGUACUCCAUGCUGCCCAGCCAGGCCAAGuga	603	200
**iRFP670**	AUGGCGCGUAAGGUCGAUCUCACCUCCUGCGAUCGCGAGCCGAUCCACAUCCCCGGCAGCAUUCAGCCGUGCGGCUGCCUGCUAGCCUGCGACGC<br>GCAGGCGGUGCGGAUCACGCGCAUUACGGAAAAUGCCGGCGCGUUCUUUGGACGCGAAACUCCGCGGGUCGGUGAGCUACUCGCCGAUUACUUCG<br>GCGAGACCGAAGCCCAUGCGCUGCGCAACGCACUGGCGCAGUCCUCCGAUCCAAAGCGACCGGCGCUGAUCUUCGGUUGGCGCGACGGCCUGACC<br>GGCCGCACCUUCGACAUCUCACUGCAUCGCCAUGACGGUACAUCGAUCAUCGAGUUCGAGCCUGCGGCGGCCGAACAGGCCGACAAUCCGCUGCG<br>GCUGACGCGGCAGAUCAUCGCGCGCACCAAAGAACUGAAGUCGCUCGAAGAGAUGGCCGCACGGGUGCCGCGCUAUCUGCAGGCGAUGCUCGGCU<br>AUCACCGCGUGAUGUUGUACCGCUUCGCGGACGACGGCUCCGGGAUGGUGAUCGGCGAGGCGAAGCGCAGCGACCUCGAGAGCUUUCUCGGUCAG<br>CACUUUCCGGCGUCGCUGGUCCCGCAGCAGGCGCGGCUACUGUACUUGAAGAACGCGAUCCGCGUGGUCUCGGAUUCGCGCGGCAUCAGCAGCCG<br>GAUCGUGCCCGAGCACGACGCCUCCGGCGCCGCGCUCGAUCUGUCGUUCGCGCACCUGCGCAGCAUCUCGCCCUGCCAUCUCGAAUUUCUGCGGA<br>ACAUGGGCGUCAGCGCCUCGAUGUCGCUGUCGAUCAUCAUUGACGGCACGCUAUGGGGAUUGAUCAUCUGUCAUCAUUACGAGCCGCGUGCCGUG<br>CCGAUGGCGCAGCGCGUCGCGGCCGAAAUGUUCGCCGACUUCUUAUCGCUGCACUUCACCGCCGCCCACCACCAACGCuga	936	311

**iRFP670$_{A118C}$**	AUGGCGCGUAAGGUCGAUCUCACCUCCUGCGAUCGCGAGCCGAUCCACAUCCCCGGCAGCAUUCAGCCGUGCGGCUGCCUGCUAGCCUGCGACGC<br>GCAGGCGGUGCGGAUCACGCGCAUUACGGAAAAUGCCGGCGCGUUCUUUGGACGCGAAACUCCGCGGGUCGGUGAGCUACUCGCCGAUUACUUCG<br>GCGAGACCGAAGCCCAUGCGCUGCGCAACGCACUGGCGCAGUCCUCCGAUCCAAAGCGACCGGCGCUGAUCUUCGGUUGGCGCGACGGCCUGACC<br>GGCCGCACCUUCGACAUCUCACUGCAUCGCCAUGACGGUACAUCGAUCAUCGAGUUCGAGCCUGCGUGCGCCGAACAGGCCGACAAUCCGCUGCG<br>GCUGACGCGGCAGAUCAUCGCGCGCACCAAAGAACUGAAGUCGCUCGAAGAGAUGGCCGCACGGGUGCCGCGCUAUCUGCAGGCGAUGCUCGGCU<br>AUCACCGCGUGAUGUUGUACCGCUUCGCGGACGACGGCUCCGGGAUGGUGAUCGGCGAGGCGAAGCGCAGCGACCUCGAGAGCUUUCUCGGUCAG<br>CACUUUCCGGCGUCGCUGGUCCCGCAGCAGGCGCGGCUACUGUACUUGAAGAACGCGAUCCGCGUGGUCUCGGAUUCGCGCGGCAUCAGCAGCCG<br>GAUCGUGCCCGAGCACGACGCCUCCGGCGCCGCGCUCGAUCUGUCGUUCGCGCACCUGCGCAGCAUCUCGCCCUGCCAUCUCGAAUUUCUGCGGA<br>ACAUGGGCGUCAGCGCCUCGAUGUCGCUGUCGAUCAUCAUUGACGGCACGCUAUGGGGAUUGAUCAUCUGUCAUCAUUACGAGCCGCGUGCCGUG<br>CCGAUGGCGCAGCGCGUCGCGGCCGAAAUGUUCGCCGACUUCUUAUCGCUGCACUUCACCGCCGCCCACCACCAACGCuga	936	311
**RF^N-Int^N**	AUGGCGCGUAAGGUCGAUCUCACCUCCUGCGAUCGCGAGCCGAUCCACAUCCCCGGCAGCAUUCAGCCGUGCGGCUGCCUGCUAGCCUGCGACGC<br>GCAGGCGGUGCGGAUCACGCGCAUUACGGAAAAUGCCGGCGCGUUCUUUGGACGCGAAACUCCGCGGGUCGGUGAGCUACUCGCCGAUUACUUCG<br>GCGAGACCGAAGCCCAUGCGCUGCGCAACGCACUGGCGCAGUCCUCCGAUCCAAAGCGACCGGCGCUGAUCUUCGGUUGGCGCGACGGCCUGACC<br>GGCCGCACCUUCGACAUCUCACUGCAUCGCCAUGACGGUACAUCGAUCAUCGAGUUCGAGCCUGCGUGCCUGAGCUACGAGACAGAGAUCCUGAC<br>CGUGGAAUACGGCCUGCUGCCUAUCGGCAAGAUCGUGGAAAAGCGGAUCGAGUGCACCGUGUACUCCGUGGAUAACAACGGCAACAUCUACACCC<br>AGCCUGUGGCUCAGUGGCACGACAGAGGCGAGCAAGAGGUGUUCGAGUACUGCCUGGAAGAUGGCAGCCUGAUCAGAGCCACCAAGGACCACAAG<br>UUCAUGACAGUGGACGGCCAGAUGCUGCCCAUCGACGAGAUCUUCGAGCGCGAGCUGGACCUGAUGAGAGUGGACAACCUGCCUAACuga	660	219
**Int^C-RF^C**	AUGAUCAAGAUCGCCACACGGAAGUACCUGGGCAAGCAGAACGUGUACGACAUCGGCGUGGAACGGGACCACAACUUCGCCCUGAAGAACGGCUU<br>UAUCGCCAGCAACUGCGCCGAACAGGCCGACAAUCCGCUGCGGCUGACGCGGCAGAUCAUCGCGCGCACCAAAGAACUGAAGUCGCUCGAAGAGA<br>UGGCCGCACGGGUGCCGCGCUAUCUGCAGGCGAUGCUCGGCUAUCACCGCGUGAUGUUGUACCGCUUCGCGGACGACGGCUCCGGGAUGGUGAUC<br>GGCGAGGCGAAGCGCAGCGACCUCGAGAGCUUUCUCGGUCAGCACUUUCCGGCGUCGCUGGUCCCGCAGCAGGCGCGGCUACUGUACUUGAAGAA<br>CGCGAUCCGCGUGGUCUCGGAUUCGCGCGGCAUCAGCAGCCGGAUCGUGCCCGAGCACGACGCCUCCGGCGCCGCGCUCGAUCUGUCGUUCGCGC<br>ACCUGCGCAGCAUCUCGCCCUGCCAUCUCGAAUUUCUGCGGAACAUGGGCGUCAGCGCCUCGAUGUCGCUGUCGAUCAUCAUUGACGGCACGCUA<br>UGGGGAUUGAUCAUCUGUCAUCAUUACGAGCCGCGUGCCGUGCCGAUGGCGCAGCGCGUCGCGGCCGAAAUGUUCGCCGACUUCUUAUCGCUGCA<br>CUUCACCGCCGCCCACCACCAACGCuga	693	230
**PAC**	AUGACCGAGUACAAGCCUACAGUGCGGCUGGCCACCAGAGAUGAUGUGCCUAGAGCCGUCAGAACACUGGCCGCUGCCUUCGCCGAUUAUCCUGC<br>CACAAGACACACCGUGGAUCCCGACAGACACAUCGAGAGAGUGACCGAGCUGCAAGAGCUGUUCCUGACCAGAGUCGGCCUGGACAUCGGCAAAG<br>UGUGGGUUGCAGAUGAUGGCGCCGCUGUGGCUGUGUGGACAACACCUGAAUCUGUGGAAGCCGGCGCUGUGUUCGCCGAGAUCGGACCUAGAAUG<br>GCCGAGCUGUCUGGCUCUAGACUGGCUGCUCAGCAGCAGAUGGAAGGACUGCUGGCCCCUCACAGACCUAAGUGCGAGCCCGCCUGGUUUCUUGC<br>UACCGUGGGAGUGUCCCCUGACCACCAAGGCAAAGGACUGGGAUCUGCUGUGGUGCUGCCUGGCGUUGAAGCCGCUGAAAGAGCUGGCGUUCCCG<br>CCUUCCUGGAAACAAGCGCCCCUAGAAACCUGCCUUUCUACGAGAGACUGGGCUUCACCGUGACCGCCGAUGUGGAAGUUCCUGAGGGCCCUAGA<br>ACCUGGUGCAUGACCAGAAAACCUGGCGCCuga	603	200
**PAC$_{Y171H}$**	AUGACCGAGUACAAGCCUACAGUGCGGCUGGCCACCAGAGAUGAUGUGCCUAGAGCCGUCAGAACACUGGCCGCUGCCUUCGCCGAUUAUCCUGC<br>CACAAGACACACCGUGGAUCCCGACAGACACAUCGAGAGAGUGACCGAGCUGCAAGAGCUGUUCCUGACCAGAGUCGGCCUGGACAUCGGCAAAG<br>UGUGGGUUGCAGAUGAUGGCGCCGCUGUGGCUGUGUGGACAACACCUGAAUCUGUGGAAGCCGGCGCUGUGUUCGCCGAGAUCGGACCUAGAAUG<br>GCCGAGCUGUCUGGCUCUAGACUGGCUGCUCAGCAGCAGAUGGAAGGACUGCUGGCCCCUCACAGACCUAAGUGCGAGCCCGCCUGGUUUCUUGC<br>UACCGUGGGAGUGUCCCCUGACCACCAAGGCAAAGGACUGGGAUCUGCUGUGGUGCUGCCUGGCGUUGAAGCCGCUGAAAGAGCUGGCGUUCCCG<br>CCUUCCUGGAAACAAGCGCCCCUAGAAACCUGCCUUUCCACGAGAGACUGGGCUUCACCGUGACCGCCGAUGUGGAAGUUCCUGAGGGCCCUAGA<br>ACCUGGUGCAUGACCAGAAAACCUGGCGCCuga	603	200
**PAC^N-Int^N**	AUGACCGAGUACAAGCCUACAGUGCGGCUGGCCACCAGAGAUGAUGUGCCUAGAGCCGUCAGAACACUGGCCGCUGCCUUCGCCGAUUAUCCUGC<br>CACAAGACACACCGUGGAUCCCGACAGACACAUCGAGAGAGUGACCGAGCUGCAAGAGCUGUUCCUGACCAGAGUCGGCCUGGACAUCGGCAAAG<br>UGUGGGUUGCAGAUGAUGGCGCCGCUGUGGCUGUGUGGACAACACCUGAAUCUGUGGAAGCCGGCGCUGUGUUCGCCGAGAUCGGACCUAGAAUG<br>GCCGAGCUGUCUGGCUCUAGACUGGCUGCUCAGCAGCAGAUGGAAGGACUGCUGGCCCCUCACAGACCUAAGUGCCUGAGCUACGAGACAGAGAU<br>CCUGACCGUGGAAUACGGCCUGCUGCCUAUCGGCAAGAUCGUGGAAAAGCGGAUCGAGUGCACCGUGUACUCCGUGGAUAACAACGGCAACAUCU<br>ACACCCAGCCUGUGGCUCAGUGGCACGACAGAGGCGAGCAAGAGGUGUUCGAGUACUGCCUGGAAGAUGGCAGCCUGAUCAGAGCCACCAAGGAC<br>CACAAGUUCAUGACAGUGGACGGCCAGAUGCUGCCCAUCGACGAGAUCUUCGAGCGCGAGCUGGACCUGAUGAGAGUGGACAACCUGCCUAACug<br>a	666	221
**Int^C-PAC^C**	AUGAUCAAGAUCGCCACACGGAAGUACCUGGGCAAGCAGAACGUGUACGACAUCGGCGUGGAACGGGACCACAACUUCGCCCUGAAGAACGGCUU<br>UAUCGCCAGCAACUGCGAGCCCGCCUGGUUUCUUGCUACCGUGGGAGUGUCCCCUGACCACCAAGGCAAAGGACUGGGAUCUGCUGUGGUGCUGC<br>CUGGCGUUGAAGCCGCUGAAAGAGCUGGCGUUCCCGCCUUCCUGGAAACAAGCGCCCCUAGAAACCUGCCUUUCUACGAGAGACUGGGCUUCACC<br>GUGACCGCCGAUGUGGAAGUUCCUGAGGGCCCUAGAACCUGGUGCAUGACCAGAAAACCUGGCGCCuga	354	117
**Int^C-PAC^Cm**	AUGAUCAAGAUCGCCACACGGAAGUACCUGGGCAAGCAGAACGUGUACGACAUCGGCGUGGAACGGGACCACAACUUCGCCCUGAAGAACGGCUU<br>UAUCGCCAGCAACUGCGAGCCCGCCUGGUUUCUUGCUACCGUGGGAGUGUCCCCUGACCACCAAGGCAAAGGACUGGGAUCUGCUGUGGUGCUGC<br>CUGGCGUUGAAGCCGCUGAAAGAGCUGGCGUUCCCGCCUUCCUGGAAACAAGCGCCCCUAGAAACCUGCCUUUCCACGAGAGACUGGGCUUCACC<br>GUGACCGCCGAUGUGGAAGUUCCUGAGGGCCCUAGAACCUGGUGCAUGACCAGAAAACCUGGCGCCuga	354	117

**HPH**	AUGAAGAAGCCUGAGCUGACCGCUACCAGCGUGGAAAAGUUCCUGAUCGAGAAGUUCGACAGCGUGUCCGACCUGAUGCAGCUGUCUGAGGGCGA<br>AGAGAGCAGAGCCUUCAGCUUCGAUGUUGGCGGCAGAGGCUACGUGCUGAGAGUGAAUUCUUGCGCCGACGGCUUCUACAAGGACAGAUACGUGU<br>ACCGGCACUUCGCCUCUGCUGCCCUGCCUAUUCCUGAGGUGCUGGACAUCGGCGAGUUCAGCGAGAGCCUGACCUACUGCAUUUCUCGGAGAGCC<br>CAGGGCGUGACCCUGCAGGAUCUUCCUGAAACAGAACUGCCCGCCGUGCUGCAGCCUGUGGCUGAAGCUAUGGAUGCCAUUGCCGCCGCUGAUCU<br>GAGCCAGACAUCUGGCUUUGGCCCCUUCGGACCUCAAGGCAUCGGCCAGUACACCACCUGGCGGGAUUUCAUCUGCGCUAUCGCCGAUCCUCACG<br>UGUACCACUGGCAGACCGUGAUGGACGAUACCGUGUCUGCCUCUGUGGCCCAGGCUCUGGAUGAACUGAUGCUGUGGGCCGAAGAUUGCCCCGAA<br>GUGCGGCAUCUGGUGCACGCCGAUUUCGGCAGCAACAACGUGCUGACCGACAACGGCAGAAUCACCGCCGUGAUCGAUUGGAGCGAGGCCAUGUU<br>UGGCGACAGCCAGUACGAGGUGGCCAAUAUCUUCUUCUGGCGGCCUUGGCUGGCCUGCAUGGAACAGCAGACCAGAUACUUCGAGCGGAGACACC<br>CUGAGCUGGCCGGAUCUCCUAGACUGAGAGCCUACAUGCUGCGGAUCGGCCUGGAUCAGCUGUACCAGUCUCUGGUGGACGGCAACUUCGACGAU<br>GCCGCUUGGGCUCAGGGAAGAUGUGAUGCUAUUGUGCGGAGCGGAGCCGGCACAGUGGGAAGAACACAAAUCGCUAGAAGAUCUGCCGCCGUGUG<br>GACCGAUGGCUGUGUGGAAGUUCUGGCCGACAGCGGCAAUAGACGGCCUAGCACAAGACCCAGAGCCAAAGAGuga	1026	341
**HPH^{N}-Int^{N}**	AUGAAGAAGCCUGAGCUGACCGCUACCAGCGUGGAAAAGUUCCUGAUCGAGAAGUUCGACAGCGUGUCCGACCUGAUGCAGCUGUCUGAGGGCGA<br>AGAGAGCAGAGCCUUCAGCUUCGAUGUUGGCGGCAGAGGCUACGUGCUGAGAGUGAAUUCUUGCGCCGACGGCUUCUACAAGGACAGAUACGUGU<br>ACCGGCACUUCGCCUCUGCUGCCCUGCCUAUUCCUGAGGUGCUGGACAUCGGCGAGUUCAGCGAGAGCCUGACCUACUGCCUGAGCUACGAGACA<br>GAGAUCCUGACCGUGGAAUACGGCCUGCUGCCUAUCGGCAAGAUCGUGGAAAAGCGGAUCGAGUGCACCGUGUACUCCGUGGAUAACAACGGCAA<br>CAUCUACACCCAGCCUGUGGCUCAGUGGCACGACAGAGGCGAGCAAGAGGUGUUCGAGUACUGCCUGGAAGAUGGCAGCCUGAUCAGAGCCACCA<br>AGGACCACAAGUUCAUGACAGUGGACGGCCAGAUGCUGCCCAUCGACGAGAUCUUCGAGCGCGAGCUGGACCUGAUGAGAGUGGACAACCUGCCU<br>AACuga	576	191
**Int^{C}-HPH^{C}**	AUGAUCAAGAUCGCCACACGGAAGUACCUGGGCAAGCAGAACGUGUACGACAUCGGCGUGGAACGGGACCACAACUUCGCCCUGAAGAACGGCUU<br>UAUCGCCAGCAACUGCAUUUCUCGGAGAGCCCAGGGCGUGACCCUGCAGGAUCUUCCUGAAACAGAACUGCCCGCCGUGCUGCAGCCUGUGGCUG<br>AAGCUAUGGAUGCCAUUGCCGCCGCUGAUCUGAGCCAGACAUCUGGCUUUGGCCCCUUCGGACCUCAAGGCAUCGGCCAGUACACCACCUGGCGG<br>GAUUUCAUCUGCGCUAUCGCCGAUCCUCACGUGUACCACUGGCAGACCGUGAUGGACGAUACCGUGUCUGCCUCUGUGGCCCAGGCUCUGGAUGA<br>ACUGAUGCUGUGGGCCGAAGAUUGCCCCGAAGUGCGGCAUCUGGUGCACGCCGAUUUCGGCAGCAACAACGUGCUGACCGACAACGGCAGAAUCA<br>CCGCCGUGAUCGAUUGGAGCGAGGCCAUGUUUGGCGACAGCCAGUACGAGGUGGCCAAUAUCUUCUUCUGGCGGCCUUGGCUGGCCUGCAUGGAA<br>CAGCAGACCAGAUACUUCGAGCGGAGACACCCUGAGCUGGCCGGAUCUCCUAGACUGAGAGCCUACAUGCUGCGGAUCGGCCUGGAUCAGCUGUA<br>CCAGUCUCUGGUGGACGGCAACUUCGACGAUGCCGCUUGGGCUCAGGGAAGAUGUGAUGCUAUUGUGCGGAGCGGAGCCGGCACAGUGGGAAGAA<br>CACAAAUCGCUAGAAGAUCUGCCGCCGUGUGGACCGAUGGCUGUGUGGAAGUUCUGGCCGACAGCGGCAAUAGACGGCCUAGCACAAGACCCAGA<br>GCCAAAGAGuga	867	288
**BSR**	AUGAAGACCUUCAACAUCAGCCAGCAGGACCUGGAACUGGUGGAAGUGGCCACCGAGAAGAUCACCAUGCUGUACGAGGACAACAAGCACCACGU<br>GGGAGCCGCCAUCAGAACAAAGACCGGCGAGAUCAUCAGCGCCGUGCACAUCGAGGCCUACAUCGGCAGAGUGACAGUGUGCGCCGAGGCCAUUG<br>CUAUCGGAAGCGCCGUGUCUAACGGCCAGAAGGACUUCGAUACCAUCGUGGCCGUGCGGCACCCUUACAGCGACGAAGUGGACAGAUCCAUCAGA<br>GUGGUGUCCCCAUGCGGCAUGUGCAGAGAGCUGAUCUCCGACUACGCCCCUGACUGCUUCGUGCUGAUCGAGAUGAACGGCAAGCUGGUCAAGAC<br>CACCAUCGAGGAACUGAUCCCUCUGAAGUACACCCGGAACuga	423	140
**BSR^{N}-Int^{N}**	AUGAAGACCUUCAACAUCAGCCAGCAGGACCUGGAACUGGUGGAAGUGGCCACCGAGAAGAUCACCAUGCUGUACGAGGACAACAAGCACCACGU<br>GGGAGCCGCCAUCAGAACAAAGACCGGCGAGAUCAUCAGCGCCGUGCACAUCGAGGCCUACAUCGGCAGAGUGACAGUGUGCGCCGAGGCCAUUG<br>CUAUCGGAAGCGCCGUGUCUAACGGCCAGAAGGACUUCGAUACCAUCGUGGCCGUGCGGCACCCUUACAGCGACGAAGUGGACAGAUCCAUCAGA<br>GUGGUGUCCCCAUGCGGCAUGUGCCUGAGCUACGAGACAGAGAUCCUGACCGUGGAAUACGGCCUGCUGCCUAUCGGCAAGAUCGUGGAAAAGCG<br>GAUCGAGUGCACCGUGUACUCCGUGGAUAACAACGGCAACAUCUACACCCAGCCUGUGGCUCAGUGGCACGACAGAGGCGAGCAAGAGGUGUUCG<br>AGUACUGCCUGGAAGAUGGCAGCCUGAUCAGAGCCACCAAGGACCACAAGUUCAUGACAGUGGACGGCCAGAUGCUGCCCAUCGACGAGAUCUUC<br>GAGCGCGAGCUGGACCUGAUGAGAGUGGACAACCUGCCUAACuga	615	204
**Int^{C}-BSR^{C}**	AUGAUCAAGAUCGCCACACGGAAGUACCUGGGCAAGCAGAACGUGUACGACAUCGGCGUGGAACGGGACCACAACUUCGCCCUGAAGAACGGCUU<br>UAUCGCCAGCAACUGCAGAGAGCUGAUCUCCGACUACGCCCCUGACUGCUUCGUGCUGAUCGAGAUGAACGGCAAGCUGGUCAAGACCACCAUCG<br>AGGAACUGAUCCCUCUGAAGUACACCCGGAACuga	225	74
**TK$_{A266C}$**	AUGGCCAGCUAUCCUUGUCACCAGCACGCCAGCGCCUUUGAUCAGGCCGCAAGAUCUAGAGGCCACAGCAACAGAAGAACAGCCCUGCGGCCUCG<br>GAGACAGCAAGAGGCUACAGAAGUUCGGCUGGAACAGAAGAUGCCCACACUGCUGCGGGUGUACAUCGAUGGCCCUCACGGCAUGGGCAAGACCA<br>CCACAACACAGCUGCUGGUGGCCCUGGGCAGCAGAGAUGAUAUCGUGUACGUGCCCGAGCCUAUGACCUACUGGCAGGUUCUGGGAGCCAGCGAG<br>ACAAUCGCCAACAUCUACACCACACAGCACCGGCUGGAUCAGGGCGAAAUUUCUGCUGGCGACGCCGCCGUGGUUAUGACAUCUGCCCAGAUCAC<br>CAUGGGCAUGCCUUACGCCGUGACAGAUGCUGUGCUGGCCCCUCACAUUGGCGGAGAAGCCGGAUCUUCUCAUGCCCCACCUCCAGCUCUGACCA<br>UCUUCCUGGACAGACACCCUAUCGCCUUCAUGCUGUGUUACCCUGCCGCCAGAUACCUGAUGGGCAGCAUGACACCUCAGGCCGUGCUGGCUUUC<br>GUGGCCCUGAUUCCUCCUACACUGCCCGGCACCAAUAUCGUGCUGGGAGCCCUGCCUGAGGACCGGCACAUUGAUAGACUGGCCAAGAGACAGCG<br>GCCUGGCGAGAGACUGGAUCUGGCUAUGCUGGCCGCCAUCAGAAGAGUGUACGGCCUGCUGGCCAACACCGUGCGGUAUCUUCAAGGCGGCGGAU<br>CUUGGAGAGAGGACUGGGGACAACUGAGCGGCACAUGUGUUCCUCCACAAGGCGCCGAGCCUCAGUCUAAUGCUGGACCCAGACCUCACAUCGGC<br>GACACCCUGUUUACCCUGUUCAGAGCCCCUGAGCUGCUGGCUCCUAACGGCGACCUGUACAACGUGUUCGCCUGGGCUCUUGACGUGCUGGCAAA<br>AAGACUGCGGCCCAUGCACGUGUUCAUCCUGGACUACGAUCAGUCCCCUGCCGGCUGUAGAGAUGCUCUGCUGCAGCUGACAAGCGGCAUGGUGC<br>AGACCCACGUUACAACCCCUGGCAGCAUCCCCACCAUCUGUGACCUGGCCAGAACCUUCGCCAGAGAGAUGGGCGAAGCCAACuga	1131	376

**TK^N-Int^N**	AUGGCCAGCUAUCCUUGUCACCAGCACGCCAGCGCCUUUGAUCAGGCCGCAAGAUCUAGAGGCCACAGCAACAGAAGAACAGCCCUGCGGCCUCG<br>GAGACAGCAAGAGGCUACAGAAGUUCGGCUGGAACAGAAGAUGCCCACACUGCUGCGGGUGUACAUCGAUGGCCCUCACGGCAUGGGCAAGACCA<br>CCACAACACAGCUGCUGGUGGCCCUGGGCAGCAGAGAUGAUAUCGUGUACGUGCCCGAGCCUAUGACCUACUGGCAGGUUCUGGGAGCCAGCGAG<br>ACAAUCGCCAACAUCUACACCACACAGCACCGGCUGGAUCAGGGCGAAAUUUCUGCUGGCGACGCCGCCGUGGUUAUGACAUCUGCCCAGAUCAC<br>CAUGGGCAUGCCUUACGCCGUGACAGAUGCUGUGCUGGCCCCUCACAUUGGCGGAGAAGCCGGAUCUUCUCAUGCCCCACCUCCAGCUCUGACCA<br>UCUUCCUGGACAGACACCCUAUCGCCUUCAUGCUGUGUUACCCUGCCGCCAGAUACCUGAUGGGCAGCAUGACACCUCAGGCCGUGCUGGCUUUC<br>GUGGCCCUGAUUCCUCCUACACUGCCCGGCACCAAUAUCGUGCUGGGAGCCCUGCCUGAGGACCGGCACAUUGAUAGACUGGCCAAGAGACAGCG<br>GCCUGGCGAGAGACUGGAUCUGGCUAUGCUGGCCGCCAUCAGAAGAGUGUACGGCCUGCUGGCCAACACCGUGCGGUAUCUUCAAGGCGGCGGAU<br>CUUGGAGAGAGGACUGGGGACAACUGAGCGGCACAUGCCUGAGCUACGAGACAGAGAUCCUGACCGUGGAAUACGGCCUGCUGCCUAUCGGCAAG<br>AUCGUGGAAAAGCGGAUCGAGUGCACCGUGUACUCCGUGGAUAACAACGGCAACAUCUACACCCAGCCUGUGGCUCAGUGGCACGACAGAGGCGA<br>GCAAGAGGUGUUCGAGUACUGCCUGGAAGAUGGCAGCCUGAUCAGAGCCACCAAGGACCACAAGUUCAUGACAGUGGACGGCCAGAUGCUGCCCA<br>UCGACGAGAUCUUCGAGCGCGAGCUGGACCUGAUGAGAGUGGACAACCUGCCUAACuga	1104	367
**Int^C-TK^C**	AUGAUCAAGAUCGCCACACGGAAGUACCUGGGCAAGCAGAACGUGUACGACAUCGGCGUGGAACGGGACCACAACUUCGCCCUGAAGAACGGCUU<br>UAUCGCCAGCAACUGUGUUCCUCCACAAGGCGCCGAGCCUCAGUCUAAUGCUGGACCCAGACCUCACAUCGGCGACACCCUGUUUACCCUGUUCA<br>GAGCCCCUGAGCUGCUGGCUCCUAACGGCGACCUGUACAACGUGUUCGCCUGGGCUCUUGACGUGCUGGCAAAAAGACUGCGGCCCAUGCACGUG<br>UUCAUCCUGGACUACGAUCAGUCCCCUGCCGGCUGUAGAGAUGCUCUGCUGCAGCUGACAAGCGGCAUGGUGCAGACCCACGUUACAACCCCUGG<br>CAGCAUCCCCACCAUCUGUGACCUGGCCAGAACCUUCGCCAGAGAGAUGGGCGAAGCCAACuga	444	147
**spCas9both**	AUGCCCAAGAAGAAGAGGAAGGUGAUGGACAAGAAGUACUCCAUUGGGCUCGAUAUCGGCACAAACAGCGUCGGCUGGGCCGUCAUUACGGACGA<br>GUACAAGGUGCCGAGCAAAAAAUUCAAAGUUCUGGGCAAUACCGAUCGCCACAGCAUAAAGAAGAACCUCAUUGGCGCCCUCCUGUUCGACUCCG<br>GGGAGACGGCCGAAGCCACGCGGCUCAAAAGAACAGCACGGCGCAGAUAUACCCGCAGAAAGAAUCGGAUCUGCUACCUGCAGGAGAUCUUUAGU<br>AAUGAGAUGGCUAAGGUGGAUGACUCUUUCUUCCAUAGGCUGGAGGAGUCCUUUUUGGUGGAGGAGGAUAAAAAGCACGAGCGCCACCCAAUCUU<br>UGGCAAUAUCGUGGACGAGGUGGCGUACCAUGAAAAGUACCCAACCAUAUAUCAUCUGAGGAAGAAGCUUGUAGACAGUACUGAUAAGGCUGACU<br>UGCGGUUGAUCUAUCUCGCGCUGGCGCAUAUGAUCAAAUUUCGGGGACACUUCCUCAUCGAGGGGGACCUGAACCCAGACAACAGCGAUGUCGAC<br>AAACUCUUUAUCCAACUGGUUCAGACUUACAAUCAGCUUUUCGAAGAGAACCCGAUCAACGCAUCCGGAGUUGACGCCAAAGCAAUCCUGAGCGC<br>UAGGCUGUCCAAAUCCCGGCGGCUCGAAAACCUCAUCGCACAGCUCCCUGGGGAGAAGAAGAACGGCCUGUUUGGUAAUCUUAUCGCCCUGUCAC<br>UCGGGCUGACCCCCAACUUUAAAUCUAACUUCGACCUGGCCGAAGAUGCCAAGCUUCAACUGAGCAAAGACACCUACGAUGAUGAUCUCGACAAU<br>CUGCUGGCCCAGAUCGGCGACCAGUACGCAGACCUUUUUUUGGCGGCAAAGAACCUGUCAGACGCCAUUCUGCUGAGUGAUAUUCUGCGAGUGAA<br>CACGGAGAUCACCAAAGCUCCGCUGAGCGCUAGUAUGAUCAAGCGCUAUGAUGAGCACCACCAAGACUUGACUUUGCUGAAGGCCCUUGUCAGAC<br>AGCAACUGCCUGAGAAGUACAAGGAAAUUUUCUUCGAUCAGUCUAAAAAUGGCUACGCCGGAUACAUUGACGGCGGAGCAAGCCAGGAGGAAUUU<br>UACAAAUUUAUUAAGCCCAUCUUGGAAAAAAUGGACGGCACCGAGGAGCUGCUGGUAAAGCUUAACAGAGAAGAUCUGUUGCGCAAACAGCGCAC<br>UUUCGACAAUGGAAGCAUCCCCCACCAGAUUCACCUGGGCGAACUGCACGCUAUCCUCAGGCGGCAAGAGGAUUUCUACCCCUUUUUGAAAGAUA<br>ACAGGGAAAAGAUUGAGAAAAUCCUCACAUUUCGGAUACCCUACUAUGUAGGCCCCCUCGCCCGGGGAAAUUCCAGAUUCGCGUGGAUGACUCGC<br>AAAUCAGAAGAGACCAUCACUCCCUGGAACUUCGAGGAAGUCGUGGAUAAGGGGGCCUCUGCCCAGUCCUUCAUCGAAAGGAUGACUAACUUUGA<br>UAAAAAUCUGCCUAACGAAAAGGUGCUUCCUAAACACUCUCUGCUGUACGAGUACUUCACAGUUUAUAACGAGCUCACCAAGGUCAAAUACGUCA<br>CAGAAGGGAUGAGAAAGCCAGCAUUCCUGUCUGGAGAGCAGAAGAAAGCUAUCGUGGACCUCCUCUUCAAGACGAACCGGAAAGUUACCGUGAAA<br>CAGCUCAAAGAAGACUAUUUCAAAAAGAUUGAAUGUUUCGACUCUGUUGAAAUCAGCGGAGUGGAGGAUCGCUUCAACGCAUCCCUGGGAACGUA<br>UCACGAUCUCCUGAAAAUCAUUAAAGACAAGGACUUCCUGGACAAUGAGGAGAACGAGGACAUUCUUGAGGACAUUGUCCUCACCCUUACGUUGU<br>UUGAAGAUAGGGAGAUGAUUGAAGAACGCUUGAAAACUUACGCUCAUCUCUUCGACGACAAAGUCAUGAAACAGCUCAAGAGGCGCCGAUAUACA<br>GGAUGGGGGCGGCUGUCAAGAAAACUGAUCAAUGGGAUCCGAGACAAGCAGAGUGGAAAGACAAUCCUGGAUUUUCUUAAGUCCGAUGGAUUUGC<br>CAACCGGAACUUCAUGCAGUUGAUCCAUGAUGACUCUCUCACCUUUAAGGAGGACAUCCAGAAAGCACAAGUUUCUGGCCAGGGGGACAGUCUUC<br>ACGAGCACAUCGCUAAUCUUGCAGGUAGCCCAGCUAUCAAAAAGGGAAUACUGCAGACCGUUAAGGUCGUGGAUGAACUCGUCAAAGUAAUGGGA<br>AGGCAUAAGCCCGAGAAUAUCGUUAUCGAGAUGGCCCGAGAGAACCAAACUACCCAGAAGGGACAGAAGAACAGUAGGGAAAGGAUGAAGAGGAU<br>UGAAGAGGGUAUAAAAGAACUGGGGUCCCAAAUCCUUAAGGAACACCCAGUUGAAAACACCCAGCUUCAGAAUGAGAAGCUCUACCUGUACUACC<br>UGCAGAACGGCAGGGACAUGUACGUGGAUCAGGAACUGGACAUCAAUCGGCUCUCCGACUACGACGUGGAUCAUAUCGUGCCCCAGUCUUUUCUC<br>AAAGAUGAUUCUAUUGAUAAUAAAGUGUUGACAAGAUCCGAUAAAAAUAGAGGGAAGAGUGAUAACGUCCCCUCAGAAGAAGUUGUCAAGAAAAU<br>GAAAAAUUAUUGGCGGCAGCUGCUGAACGCCAAACUGAUCACACAACGGAAGUUCGAUAAUCUGACUAAGGCUGAACGAGGUGGCCUGUCUGAGU<br>UGGAUAAAGCCGGCUUCAUCAAAAGGCAGCUUGUUGAGACACGCCAGAUCACCAAGCACGUGGCCCAAAUUCUCGAUUCACGCAUGAACACCAAG<br>UACGAUGAAAAUGACAAACUGAUUCGAGAGGUGAAAGUUAUUACUCUGAAGUCUAAGCUGGUCUCAGAUUUCAGAAAGGACUUUCAGUUUUAUAA<br>GGUGAGAGAGAUCAACAAUUACCACCAUGCGCAUGAUGCCUACCUGAAUGCAGUGGUAGGCACUGCACUUAUCAAAAAAUAUCCCAAGCUUGAAU<br>CUGAAUUUGUUUACGGAGACUAUAAAGUGUACGAUGUUAGGAAAAUGAUCGCAAAGUCUGAGCAGGAAAUAGGCAAGGCCACCGCUAAGUACUUC<br>UUUUACAGCAAUAUUAUGAAUUUUUUCAAGACCGAGAUUACACUGGCCAAUGGAGAGAUUCGGAAGCGACCACUUAUCGAAACAAACGGAGAAAC<br>AGGAGAAAUCGUGUGGGACAAGGGUAGGGAUUUCGCGACAGUCCGGAAGGUCCUGUCCAUGCCGCAGGUGAACAUCGUUAAAAAGACCGAAGUAC<br>AGACCGGAGGCUUCUCCAAGGAAAGUAUCCUCCCGAAAAGGAACAGCGACAAGCUGAUCGCACGCAAAAAAGAUUGGGACCCCAAGAAAUACGGC<br>GGAUUCGAUUCUCCUACAGUCGCUUACAGUGUACUGGUUGUGGCCAAAGUGGAGAAAGGGAAGUCUAAAAAACUCAAAAGCGUCAAGGAACUGCU<br>GGGCAUCACAAUCAUGGAGCGAUCAAGCUUCGAAAAAAACCCCAUCGACUUUCUCGAGGCGAAAGGAUAUAAAGAGGUCAAAAAAGACCUCAUCA<br>UUAAGCUUCCCAAGUACUCUCUCUUUGAGCUUGAAAACGGCCGGAAACGAAUGCUCGCUAGUGCGGGCGAGCUGCAGAAAGGUAACGAGCUGGCA<br>CUGCCCUCUAAAUACGUUAAUUUCUUGUAUCUGGCCAGCCACUAUGAAAAGCUCAAAGGGUCUCCCGAAGAUAAUGAGCAGAAGCAGCUGUUCGU<br>GGAACAACACAAACACUACCUUGAUGAGAUCAUCGAGCAAAUAAGCGAAUUCUCCAAAAGAGUGAUCCUCGCCGACGCUAACCUCGAUAAGGUGC<br>UUUCUGCUUACAAUAAGCACAGGGAUAAGCCCAUCAGGGAGCAGGCAGAAAACAUUAUCCACUUGUUUACUCUGACCAACUUGGGCGCGCCUGCA<br>GCCUUCAAGUACUUCGACACCACCAUAGACAGAAAGCGGUACACCUCUACAAAGGAGGUCCUGGACGCCACACUGAUUCAUCAGUCAAUUACGGG<br>GCUCUAUGAAACAAGAAUCGACCUCUCUCAGCUCGGUGGAGACAGCAGGGCUGACCCCAAGAAGAAGAGGAAGGUGuga	4164	1387

**NLS-spCas9^{N}-Int^{N}**	AUGCCCAAGAAGAAGAGGAAGGUGAUGGACAAGAAGUACUCCAUUGGGCUCGAUAUCGGCACAAACAGCGUCGGCUGGGCCGUCAUUACGGACGA<br>GUACAAGGUGCCGAGCAAAAAAUUCAAAGUUCUGGGCAAUACCGAUCGCCACAGCAUAAAGAAGAACCUCAUUGGCGCCCUCCUGUUCGACUCCG<br>GGGAGACGGCCGAAGCCACGCGGCUCAAAAGAACAGCACGGCGCAGAUAUACCCGCAGAAAGAAUCGGAUCUGCUACCUGCAGGAGAUCUUUAGU<br>AAUGAGAUGGCUAAGGUGGAUGACUCUUUCUUCCAUAGGCUGGAGGAGUCCUUUUUGGUGGAGGAGGAUAAAAAGCACGAGCGCCACCCAAUCUU<br>UGGCAAUAUCGUGGACGAGGUGGCGUACCAUGAAAAGUACCCAACCAUAUAUCAUCUGAGGAAGAAGCUUGUAGACAGUACUGAUAAGGCUGACU<br>UGCGGUUGAUCUAUCUCGCGCUGGCGCAUAUGAUCAAAUUUCGGGGACACUUCCUCAUCGAGGGGGACCUGAACCCAGACAACAGCGAUGUCGAC<br>AAACUCUUUAUCCAACUGGUUCAGACUUACAAUCAGCUUUUCGAAGAGAACCCGAUCAACGCAUCCGGAGUUGACGCCAAAGCAAUCCUGAGCGC<br>UAGGCUGUCCAAAUCCCGGCGGCUCGAAAACCUCAUCGCACAGCUCCCUGGGGAGAAGAAGAACGGCCUGUUUGGUAAUCUUAUCGCCCUGUCAC<br>UCGGGCUGACCCCCAACUUUAAAUCUAACUUCGACCUGGCCGAAGAUGCCAAGCUUCAACUGAGCAAAGACACCUACGAUGAUGAUCUCGACAAU<br>CUGCUGGCCCAGAUCGGCGACCAGUACGCAGACCUUUUUUUGGCGGCAAAGAACCUGUCAGACGCCAUUCUGCUGAGUGAUAUUCUGCGAGUGAA<br>CACGGAGAUCACCAAAGCUCCGCUGAGCGCUAGUAUGAUCAAGCGCUAUGAUGAGCACCACCAAGACUUGACUUUGCUGAAGGCCCUUGUCAGAC<br>AGCAACUGCCUGAGAAGUACAAGGAAAUUUUCUUCGAUCAGUCUAAAAAUGGCUACGCCGGAUACAUUGACGGCGGAGCAAGCCAGGAGGAAUUU<br>UACAAAUUUAUUAAGCCCAUCUUGGAAAAAAUGGACGGCACCGAGGAGCUGCUGGUAAAGCUUAACAGAGAAGAUCUGUUGCGCAAACAGCGCAC<br>UUUCGACAAUGGAAGCAUCCCCCACCAGAUUCACCUGGGCGAACUGCACGCUAUCCUCAGGCGGCAAGAGGAUUUCUACCCCUUUUUGAAAGAUA<br>ACAGGGAAAAGAUUGAGAAAAUCCUCACAUUUCGGAUACCCUACUAUGUAGGCCCCCUCGCCCGGGGAAAUUCCAGAUUCGCGUGGAUGACUCGC<br>AAAUCAGAAGAGACCAUCACUCCCUGGAACUUCGAGGAAGUCGUGGAUAAGGGGGCCUCUGCCCAGUCCUUCAUCGAAAGGAUGACUAACUUUGA<br>UAAAAAUCUGCCUAACGAAAAGGUGCUUCCUAAACACUCUCUGCUGUACGAGUACUUCACAGUUUAUAACGAGCUCACCAAGGUCAAAUACGUCA<br>CAGAAGGGAUGAGAAAGCCAGCAUUCCUGUCUGGAGAGCAGAAGAAAGCUAUCGUGGACCUCCUCUUCAAGACGAACCGGAAAGUUACCGUGAAA<br>CAGCUCAAAGAAGACUAUUUCAAAAAGAUUGAAUGCCUGAGCUACGAGACAGAGAUCCUGACCGUGGAAUACGGCCUGCUGCCUAUCGGCAAGAU<br>CGUGGAAAAGCGGAUCGAGUGCACCGUGUACUCCGUGGAUAACAACGGCAACAUCUACACCCAGCCUGUGGCUCAGUGGCACGACAGAGGCGAGC<br>AAGAGGUGUUCGAGUACUGCCUGGAAGAUGGCAGCCUGAUCAGAGCCACCAAGGACCACAAGUUCAUGACAGUGGACGGCCAGAUGCUGCCCAUC<br>GACGAGAUCUUCGAGCGCGAGCUGGACCUGAUGAGAGUGGACAACCUGCCUAACuga	2052	683
**Int^{C}-spCas9^{C}-NLS**	AUGAUCAAGAUCGCCACACGGAAGUACCUGGGCAAGCAGAACGUGUACGACAUCGGCGUGGAACGGGACCACAACUUCGCCCUGAAGAACGGCUU<br>UAUCGCCAGCAACUGUUUCGACUCUGUUGAAAUCAGCGGAGUGGAGGAUCGCUUCAACGCAUCCCUGGGAACGUAUCACGAUCUCCUGAAAAUCA<br>UUAAAGACAAGGACUUCCUGGACAAUGAGGAGAACGAGGACAUUCUUGAGGACAUUGUCCUCACCCUUACGUUGUUUGAAGAUAGGGAGAUGAUU<br>GAAGAACGCUUGAAAACUUACGCUCAUCUCUUCGACGACAAAGUCAUGAAACAGCUCAAGAGGCGCCGAUAUACAGGAUGGGGGCGGCUGUCAAG<br>AAAACUGAUCAAUGGGAUCCGAGACAAGCAGAGUGGAAAGACAAUCCUGGAUUUUCUUAAGUCCGAUGGAUUUGCCAACCGGAACUUCAUGCAGU<br>UGAUCCAUGAUGACUCUCUCACCUUUAAGGAGGACAUCCAGAAAGCACAAGUUUCUGGCCAGGGGGACAGUCUUCACGAGCACAUCGCUAAUCUU<br>GCAGGUAGCCCAGCUAUCAAAAAGGGAAUACUGCAGACCGUUAAGGUCGUGGAUGAACUCGUCAAAGUAAUGGGAAGGCAUAAGCCCGAGAAUAU<br>CGUUAUCGAGAUGGCCCGAGAGAACCAAACUACCCAGAAGGGACAGAAGAACAGUAGGGAAAGGAUGAAGAGGAUUGAAGAGGGUAUAAAAGAAC<br>UGGGGUCCCAAAUCCUUAAGGAACACCCAGUUGAAAACACCCAGCUUCAGAAUGAGAAGCUCUACCUGUACUACCUGCAGAACGGCAGGGACAUG<br>UACGUGGAUCAGGAACUGGACAUCAAUCGGCUCUCCGACUACGACGUGGAUCAUAUCGUGCCCCAGUCUUUUCUCAAAGAUGAUUCUAUUGAUAA<br>UAAAGUGUUGACAAGAUCCGAUAAAAAUAGAGGGAAGAGUGAUAACGUCCCCUCAGAAGAAGUUGUCAAGAAAAUGAAAAAUUAUUGGCGGCAGC<br>UGCUGAACGCCAAACUGAUCACACAACGGAAGUUCGAUAAUCUGACUAAGGCUGAACGAGGUGGCCUGUCUGAGUUGGAUAAAGCCGGCUUCAUC<br>AAAAGGCAGCUUGUUGAGACACGCCAGAUCACCAAGCACGUGGCCCAAAUUCUCGAUUCACGCAUGAACACCAAGUACGAUGAAAAUGACAAACU<br>GAUUCGAGAGGUGAAAGUUAUUACUCUGAAGUCUAAGCUGGUCUCAGAUUUCAGAAAGGACUUUCAGUUUUAUAAGGUGAGAGAGAUCAACAAUU<br>ACCACCAUGCGCAUGAUGCCUACCUGAAUGCAGUGGUAGGCACUGCACUUAUCAAAAAAUAUCCCAAGCUUGAAUCUGAAUUUGUUUACGGAGAC<br>UAUAAAGUGUACGAUGUUAGGAAAAUGAUCGCAAAGUCUGAGCAGGAAAUAGGCAAGGCCACCGCUAAGUACUUCUUUUACAGCAAUAUUAUGAA<br>UUUUUUCAAGACCGAGAUUACACUGGCCAAUGGAGAGAUUCGGAAGCGACCACUUAUCGAAACAAACGGAGAAACAGGAGAAAUCGUGUGGGACA<br>AGGGUAGGGAUUUCGCGACAGUCCGGAAGGUCCUGUCCAUGCCGCAGGUGAACAUCGUUAAAAAGACCGAAGUACAGACCGGAGGCUUCUCCAAG<br>GAAAGUAUCCUCCCGAAAAGGAACAGCGACAAGCUGAUCGCACGCAAAAAAGAUUGGGACCCCAAGAAAUACGGCGGAUUCGAUUCUCCUACAGU<br>CGCUUACAGUGUACUGGUUGUGGCCAAAGUGGAGAAAGGGAAGUCUAAAAAACUCAAAAGCGUCAAGGAACUGCUGGGCAUCACAAUCAUGGAGC<br>GAUCAAGCUUCGAAAAAAACCCCAUCGACUUUCUCGAGGCGAAAGGAUAUAAAGAGGUCAAAAAAGACCUCAUCAUUAAGCUUCCCAAGUACUCU<br>CUCUUUGAGCUUGAAAACGGCCGGAAACGAAUGCUCGCUAGUGCGGGCGAGCUGCAGAAAGGUAACGAGCUGGCACUGCCCUCUAAAUACGUUAA<br>UUUCUUGUAUCUGGCCAGCCACUAUGAAAAGCUCAAAGGGUCUCCCGAAGAUAAUGAGCAGAAGCAGCUGUUCGUGGAACAACACAAACACUACC<br>UUGAUGAGAUCAUCGAGCAAAUAAGCGAAUUCUCCAAAAGAGUGAUCCUCGCCGACGCUAACCUCGAUAAGGUGCUUUCUGCUUACAAUAAGCAC<br>AGGGAUAAGCCCAUCAGGGAGCAGGCAGAAAACAUUAUCCACUUGUUUACUCUGACCAACUUGGGCGCGCCUGCAGCCUUCAAGUACUUCGACAC<br>CACCAUAGACAGAAAGCGGUACACCUCUACAAAGGAGGUCCUGGACGCCACACUGAUUCAUCAGUCAAUUACGGGGCUCUAUGAAACAAGAAUCG<br>ACCUCUCUCAGCUCGGUGGAGACAGCAGGGCUGACCCCAAGAAGAAGAGGAAGGUGuga	2529	842
**L7Ae**	AUGUACGUGAGAUUUGAGGUUCCUGAGGACAUGCAGAACGAAGCUCUGAGUCUGCUGGAGAAGGUUAGGGAGAGCGGUAAGGUAAAGAAAGGUAC<br>CAACGAGACGACAAAGGCUGUGGAGAGGGGACUGGCAAAGCUCGUUUACAUCGCAGAGGAUGUUGACCCGCCUGAGAUCGUUGCUCAUCUGCCCC<br>UCCUCUGCGAGGAGAAGAAUGUGCCGUACAUUUACGUUAAAAGCAAGAACGACCUUGGAAGGGCUGUGGGCAUUGAGGUGCCAUGCGCUUCGGCA<br>GCGAUAAUCAACGAGGGAGAGCUGAGAAAGGAGCUUGGAAGCCUUGUGGAGAAGAUUAAAGGCCUUCAGAAGuga	360	119
**LIN28A**	AUGGGCAGCGUGUCCAAUCAGCAAUUUGCCGGCGGAUGUGCCAAGGCCGCUGAAGAAGCUCCAGAAGAAGCACCUGAGGACGCCGCCAGAGCUGC<br>UGAUGAACCUCAACUGCUUCACGGCGCUGGCAUCUGCAAGUGGUUCAAUGUGCGGAUGGGCUUCGGCUUCCUGAGCAUGACAGCUAGAGCUGGCG<br>UGGCCCUGGAUCCUCCUGUGGAUGUGUUUGUGCACCAGAGCAAGCUGCACAUGGAAGGCUUCCGCAGCCUGAAAGAGGGCGAAGCCGUCGAGUUC<br>ACCUUCAAGAAGUCUGCCAAGGGCCUCGAGAGCAUCAGAGUGACAGGACCUGGCGGAGUGUUCUGCAUCGGCUCUGAGAGAAGGCCCAAGGGCAA<br>GAGCAUGCAGAAGCGGAGAAGCAAGGGCGACAGAUGCUACAACUGUGGCGGCCUGGAUCACCACGCCAAAGAGUGCAAACUGCCUCCUCAGCCUA<br>AGAAGUGCCACUUUUGCCAGAGCAUCUCCCACAUGGUGGCCAGCUGUCCUCUGAAAGCUCAGCAGGGACCUAGCGCUCAGGGCAAGCCUACCUAC<br>UUCAGAGAGGAAGAGGAAGAAAUUCACAGCCCCACACUGCUGCCCGAGGCUCAGAAUuga	630	209

**AG$_{\text{Q63C, P131S}}$**	AUGGUGAGCGUGAUCAAGCCCGAGAUGAAGAUCAAGCUGUGCAUGAGGGGCACCGUGAACGGCCACAACUUCGUGAUCGAGGGCGAGGGCAAGGG<br>CAACCCCUACGAGGGCACCCAGAUCCUGGACCUGAACGUGACCGAGGGCGCCCCCCUGCCCUUCGCCUACGACAUCCUGACCACCGUGUUCUGCU<br>ACGGCAACAGGGCCUUCACCAAGUACCCCGCCGACAUCCAGGACUACUUCAAGCAGACCUUCCCCGAGGGCUACCACUGGGAGAGGAGCAUGACC<br>UACGAGGACCAGGGCAUCUGCACCGCCACCAGCAACAUCAGCAUGAGGGGCGACUGCUUCUUCUACGACAUCAGGUUCGACGGCACCAACUUCCC<br>CCCCAACGGCAGCGUGAUGCAGAAGAAGACCCUGAAGUGGGAGCCCAGCACCGAGAAGAUGUACGUGGAGGACGGCGUGCUGAAGGGCGACGUGA<br>ACAUGAGGCUGCUGCUGGAGGGCGGCGGCCACUACAGGUGCGACUUCAAGACCACCUACAAGGCCAAGAAGGAGGUGAGGCUGCCCGACGCCCAC<br>AAGAUCGACCACAGGAUCGAGAUCCUGAAGCACGACAAGGACUACAACAAGGUGAAGCUGUACGAGAACGCCGUGGCCAGGUACUCCAUGCUGCC<br>CAGCCAGGCCAAGuga	681	226
**AG$_{\text{Q63C, V153S}}$**	AUGGUGAGCGUGAUCAAGCCCGAGAUGAAGAUCAAGCUGUGCAUGAGGGGCACCGUGAACGGCCACAACUUCGUGAUCGAGGGCGAGGGCAAGGG<br>CAACCCCUACGAGGGCACCCAGAUCCUGGACCUGAACGUGACCGAGGGCGCCCCCCUGCCCUUCGCCUACGACAUCCUGACCACCGUGUUCUGCU<br>ACGGCAACAGGGCCUUCACCAAGUACCCCGCCGACAUCCAGGACUACUUCAAGCAGACCUUCCCCGAGGGCUACCACUGGGAGAGGAGCAUGACC<br>UACGAGGACCAGGGCAUCUGCACCGCCACCAGCAACAUCAGCAUGAGGGGCGACUGCUUCUUCUACGACAUCAGGUUCGACGGCACCAACUUCCC<br>CCCCAACGGCCCCGUGAUGCAGAAGAAGACCCUGAAGUGGGAGCCCAGCACCGAGAAGAUGUACGUGGAGGACGGCAGCCUGAAGGGCGACGUGA<br>ACAUGAGGCUGCUGCUGGAGGGCGGCGGCCACUACAGGUGCGACUUCAAGACCACCUACAAGGCCAAGAAGGAGGUGAGGCUGCCCGACGCCCAC<br>AAGAUCGACCACAGGAUCGAGAUCCUGAAGCACGACAAGGACUACAACAAGGUGAAGCUGUACGAGAACGCCGUGGCCAGGUACUCCAUGCUGCC<br>CAGCCAGGCCAAGuga	681	226
**AG$_{\text{Q63C, G167S}}$**	AUGGUGAGCGUGAUCAAGCCCGAGAUGAAGAUCAAGCUGUGCAUGAGGGGCACCGUGAACGGCCACAACUUCGUGAUCGAGGGCGAGGGCAAGGG<br>CAACCCCUACGAGGGCACCCAGAUCCUGGACCUGAACGUGACCGAGGGCGCCCCCCUGCCCUUCGCCUACGACAUCCUGACCACCGUGUUCUGCU<br>ACGGCAACAGGGCCUUCACCAAGUACCCCGCCGACAUCCAGGACUACUUCAAGCAGACCUUCCCCGAGGGCUACCACUGGGAGAGGAGCAUGACC<br>UACGAGGACCAGGGCAUCUGCACCGCCACCAGCAACAUCAGCAUGAGGGGCGACUGCUUCUUCUACGACAUCAGGUUCGACGGCACCAACUUCCC<br>CCCCAACGGCCCCGUGAUGCAGAAGAAGACCCUGAAGUGGGAGCCCAGCACCGAGAAGAUGUACGUGGAGGACGGCGUGCUGAAGGGCGACGUGA<br>ACAUGAGGCUGCUGCUGGAGGGCAGCGGCCACTACAGGTGCGACTTCAAGACCACCTACAAGGCCAAGAAGGAGGTGAGGCTGCCCGACGCCCAC<br>AAGATCGACCACAGGATCGAGATCCTGAAGCACGACAAGGACTACAACAAGGTGAAGCTGTACGAGAACGCCGTGGCCAGGTACTCCATGCTGCC<br>CAGCCAGGCCAAGtga	681	226
**AG$_{\text{P131S}}$-aN**	AUGGUGAGCGUGAUCAAGCCCGAGAUGAAGAUCAAGCUGUGCAUGAGGGGCACCGUGAACGGCCACAACUUCGUGAUCGAGGGCGAGGGCAAGGG<br>CAACCCCUACGAGGGCACCCAGAUCCUGGACCUGAACGUGACCGAGGGCGCCCCCCUGCCCUUCGCCUACGACAUCCUGACCACCGUGUUCUGCU<br>ACGGCAACAGGGCCUUCACCAAGUACCCCGCCGACAUCCAGGACUACUUCAAGCAGACCUUCCCCGAGGGCUACCACUGGGAGAGGAGCAUGACC<br>UACGAGGACCAGGGCAUCUGCACCGCCACCAGCAACAUCAGCAUGAGGGGCGACUGCUUCUUCUACGACAUCAGGUUCGACGGCACCAACUUCCC<br>CCCCAACGGCUGCAUCAGCGGCGAUAGCCUGAUCUCUCUGGCCuga	426	141
**AG$_{\text{P131S}}$-aC**	AUGAGCACAGGCAAGAGAGUGCCCAUCAAGGAUCUGCUGGGCGAGAAGGACUUCGAGAUCUGGGCCAUCAACGAGCAGACCAUGAAGCUGGAAAG<br>CGCCAAGGUGUCCCGGGUGUUCUGCACCGGAAAGAAACUGGUGUACACACUGAAAACCCGGCUGGGCAGAACCAUCAAGGCCACCGCCAACCACC<br>GGUUUCUGACAAUCGACGGCUGGAAGAGACUGGACGAGCUGAGCCUGAAAGAGCACAUUGCCCUGCCUAGAAAGCUGGAAUCCAGCAGCCUGCAG<br>CUGGCCCCUGAGAUUGAGAAGCUGCCCCAGAGCGACAUCUACUGGGACCCCAUCGUGUCCAUCACCGAGACAGGCGUGGAAGAGGUGUUCGACCU<br>GACAGUGCCCGGCCUGAGAAACUUCGUGGCCAACGACAUCAUCGUGCACAACAGCGUGAUGCAGAAGAAGACCCUGAAGUGGGAGCCCAGCACCG<br>AGAAGAUGUACGUGGAGGACGGCGUGCUGAAGGGCGACGUGAACAUGAGGCUGCUGCUGGAGGGCGGCGGCCACUACAGGUGCGACUUCAAGACC<br>ACCUACAAGGCCAAGAAGGAGGUGAGGCUGCCCGACGCCCACAAGAUCGACCACAGGAUCGAGAUCCUGAAGCACGACAAGGACUACAACAAGGU<br>GAAGCUGUACGAGAACGCCGUGGCCAGGUACUCCAUGCUGCCCAGCCAGGCCAAGuga	723	240
**AG$_{\text{P131S}}$-bN**	AUGGUGAGCGUGAUCAAGCCCGAGAUGAAGAUCAAGCUGUGCAUGAGGGGCACCGUGAACGGCCACAACUUCGUGAUCGAGGGCGAGGGCAAGGG<br>CAACCCCUACGAGGGCACCCAGAUCCUGGACCUGAACGUGACCGAGGGCGCCCCCCUGCCCUUCGCCUACGACAUCCUGACCACCGUGUUCUGCU<br>ACGGCAACAGGGCCUUCACCAAGUACCCCGCCGACAUCCAGGACUACUUCAAGCAGACCUUCCCCGAGGGCUACCACUGGGAGAGGAGCAUGACC<br>UACGAGGACCAGGGCAUCUGCACCGCCACCAGCAACAUCAGCAUGAGGGGCGACUGCUUCUUCUACGACAUCAGGUUCGACGGCACCAACUUCCC<br>CCCCAACGGCUGCAUCAGCGGCGAUAGCCUGAUCUCUCUGGCCAGCACAGGCAAGAGAGUGCCCAUCAAGGAUCUGCUGGGCGAGAAGGACUUCG<br>AGAUCUGGGCCAUCAACGAGCAGACCAUGAAGCUGGAAAGCGCCAAGGUGUCCCGGGUGUUCUGCACCGGAAAGAAACUGGUGUACACACUGAAA<br>ACCCGGCUGGGCAGAACCAUCAAGGCCACCGCCAACCACCGGUUUCUGACAAUCGACGGCUGGAAGAGACUGGACGAGCUGAGCCUGAAAGAGCA<br>CAUUGCCCUGCCUAGAAAGCUGGAAUCCAGCAGCCUGCAGuga	708	235
**AG$_{\text{P131S}}$-bC**	AUGCUGGCCCCUGAGAUUGAGAAGCUGCCCCAGAGCGACAUCUACUGGGACCCCAUCGUGUCCAUCACCGAGACAGGCGUGGAAGAGGUGUUCGA<br>CCUGACAGUGCCCGGCCUGAGAAACUUCGUGGCCAACGACAUCAUCGUGCACAACAGCGUGAUGCAGAAGAAGACCCUGAAGUGGGAGCCCAGCA<br>CCGAGAAGAUGUACGUGGAGGACGGCGUGCUGAAGGGCGACGUGAACAUGAGGCUGCUGCUGGAGGGCGGCGGCCACUACAGGUGCGACUUCAAG<br>ACCACCUACAAGGCCAAGAAGGAGGUGAGGCUGCCCGACGCCCACAAGAUCGACCACAGGAUCGAGAUCCUGAAGCACGACAAGGACUACAACAA<br>GGUGAAGCUGUACGAGAACGCCGUGGCCAGGUACUCCAUGCUGCCCAGCCAGGCCAAGuga	441	146
**AG$_{\text{V153S}}$-aN**	AUGGUGAGCGUGAUCAAGCCCGAGAUGAAGAUCAAGCUGUGCAUGAGGGGCACCGUGAACGGCCACAACUUCGUGAUCGAGGGCGAGGGCAAGGG<br>CAACCCCUACGAGGGCACCCAGAUCCUGGACCUGAACGUGACCGAGGGCGCCCCCCUGCCCUUCGCCUACGACAUCCUGACCACCGUGUUCUGCU<br>ACGGCAACAGGGCCUUCACCAAGUACCCCGCCGACAUCCAGGACUACUUCAAGCAGACCUUCCCCGAGGGCUACCACUGGGAGAGGAGCAUGACC<br>UACGAGGACCAGGGCAUCUGCACCGCCACCAGCAACAUCAGCAUGAGGGGCGACUGCUUCUUCUACGACAUCAGGUUCGACGGCACCAACUUCCC<br>CCCCAACGGCCCCGUGAUGCAGAAGAAGACCCUGAAGUGGGAGCCCAGCACCGAGAAGAUGUACGUGGAGGACGGCUGCAUCAGCGGCGAUAGCC<br>UGAUCUCUCUGGCCuga	492	163
**AG$_{\text{V153S}}$-aC**	AUGAGCACAGGCAAGAGAGUGCCCAUCAAGGAUCUGCUGGGCGAGAAGGACUUCGAGAUCUGGGCCAUCAACGAGCAGACCAUGAAGCUGGAAAG<br>CGCCAAGGUGUCCCGGGUGUUCUGCACCGGAAAGAAACUGGUGUACACACUGAAAACCCGGCUGGGCAGAACCAUCAAGGCCACCGCCAACCACC<br>GGUUUCUGACAAUCGACGGCUGGAAGAGACUGGACGAGCUGAGCCUGAAAGAGCACAUUGCCCUGCCUAGAAAGCUGGAAUCCAGCAGCCUGCAG<br>CUGGCCCCUGAGAUUGAGAAGCUGCCCCAGAGCGACAUCUACUGGGACCCCAUCGUGUCCAUCACCGAGACAGGCGUGGAAGAGGUGUUCGACCU<br>GACAGUGCCCGGCCUGAGAAACUUCGUGGCCAACGACAUCAUCGUGCACAACAGCCUGAAGGGCGACGUGAACAUGAGGCUGCUGCUGGAGGGCG<br>GCGGCCACUACAGGUGCGACUUCAAGACCACCUACAAGGCCAAGAAGGAGGUGAGGCUGCCCGACGCCCACAAGAUCGACCACAGGAUCGAGAUC<br>CUGAAGCACGACAAGGACUACAACAAGGUGAAGCUGUACGAGAACGCCGUGGCCAGGUACUCCAUGCUGCCCAGCCAGGCCAAGuga	657	218

**AG$_{V153S}$-bN**	AUGGUGAGCGUGAUCAAGCCCGAGAUGAAGAUCAAGCUGUGCAUGAGGGGCACCGUGAACGGCCACAACUUCGUGAUCGAGGGCGAGGGCAAGGG<br>CAACCCCUACGAGGGCACCCAGAUCCUGGACCUGAACGUGACCGAGGGCGCCCCCCUGCCCUUCGCCUACGACAUCCUGACCACCGUGUUCUGCU<br>ACGGCAACAGGGCCUUCACCAAGUACCCCGCCGACAUCCAGGACUACUUCAAGCAGACCUUCCCCGAGGGCUACCACUGGGAGAGGAGCAUGACC<br>UACGAGGACCAGGGCAUCUGCACCGCCACCAGCAACAUCAGCAUGAGGGGCGACUGCUUCUUCUACGACAUCAGGUUCGACGGCACCAACUUCCC<br>CCCCAACGGCCCCGUGAUGCAGAAGAAGACCCUGAAGUGGGAGCCCAGCACCGAGAAGAUGUACGUGGAGGACGGCUGCAUCAGCGGCGAUAGCC<br>UGAUCUCUCUGGCCAGCACAGGCAAGAGAGUGCCCAUCAAGGAUCUGCUGGGCGAGAAGGACUUCGAGAUCUGGGCCAUCAACGAGCAGACCAUG<br>AAGCUGGAAAGCGCCAAGGUGUCCCGGGUGUUCUGCACCGGAAAGAAACUGGUGUACACACUGAAAACCCGGCUGGGCAGAACCAUCAAGGCCAC<br>CGCCAACCACCGGUUUCUGACAAUCGACGGCUGGAAGAGACUGGACGAGCUGAGCCUGAAAGAGCACAUUGCCCUGCCUAGAAAGCUGGAAUCCA<br>GCAGCCUGCAGuga	774	257
**AG$_{V153S}$-bC**	AUGCUGGCCCCUGAGAUUGAGAAGCUGCCCCAGAGCGACAUCUACUGGGACCCCAUCGUGUCCAUCACCGAGACAGGCGUGGAAGAGGUGUUCGA<br>CCUGACAGUGCCCGGCCUGAGAAACUUCGUGGCCAACGACAUCAUCGUGCACAACAGCCUGAAGGGCGACGUGAACAUGAGGCUGCUGCUGGAGG<br>GCGGCGGCCACUACAGGUGCGACUUCAAGACCACCUACAAGGCCAAGAAGGAGGUGAGGCUGCCCGACGCCCACAAGAUCGACCACAGGAUCGAG<br>AUCCUGAAGCACGACAAGGACUACAACAAGGUGAAGCUGUACGAGAACGCCGUGGCCAGGUACUCCAUGCUGCCCAGCCAGGCCAAGuga	375	124
**AG$_{G167S}$-aN**	AUGGUGAGCGUGAUCAAGCCCGAGAUGAAGAUCAAGCUGUGCAUGAGGGGCACCGUGAACGGCCACAACUUCGUGAUCGAGGGCGAGGGCAAGGG<br>CAACCCCUACGAGGGCACCCAGAUCCUGGACCUGAACGUGACCGAGGGCGCCCCCCUGCCCUUCGCCUACGACAUCCUGACCACCGUGUUCUGCU<br>ACGGCAACAGGGCCUUCACCAAGUACCCCGCCGACAUCCAGGACUACUUCAAGCAGACCUUCCCCGAGGGCUACCACUGGGAGAGGAGCAUGACC<br>UACGAGGACCAGGGCAUCUGCACCGCCACCAGCAACAUCAGCAUGAGGGGCGACUGCUUCUUCUACGACAUCAGGUUCGACGGCACCAACUUCCC<br>CCCCAACGGCCCCGUGAUGCAGAAGAAGACCCUGAAGUGGGAGCCCAGCACCGAGAAGAUGUACGUGGAGGACGGCGUGCUGAAGGGCGACGUGA<br>ACAUGAGGCUGCUGCUGGAGGGCUGCAUCAGCGGCGAUAGCCUGAUCUCUCUGGCCuga	534	177
**AG$_{G167S}$-aC**	AUGAGCACAGGCAAGAGAGUGCCCAUCAAGGAUCUGCUGGGCGAGAAGGACUUCGAGAUCUGGGCCAUCAACGAGCAGACCAUGAAGCUGGAAAG<br>CGCCAAGGUGUCCCGGGUGUUCUGCACCGGAAAGAAACUGGUGUACACACUGAAAACCCGGCUGGGCAGAACCAUCAAGGCCACCGCCAACCACC<br>GGUUUCUGACAAUCGACGGCUGGAAGAGACUGGACGAGCUGAGCCUGAAAGAGCACAUUGCCCUGCCUAGAAAGCUGGAAUCCAGCAGCCUGCAG<br>CUGGCCCCUGAGAUUGAGAAGCUGCCCCAGAGCGACAUCUACUGGGACCCCAUCGUGUCCAUCACCGAGACAGGCGUGGAAGAGGUGUUCGACCU<br>GACAGUGCCCGGCCUGAGAAACUUCGUGGCCAACGACAUCAUCGUGCACAACAGCGGCCACTACAGGTGCGACTTCAAGACCACCTACAAGGCCA<br>AGAAGGAGGTGAGGCTGCCCGACGCCCACAAGATCGACCACAGGATCGAGATCCTGAAGCACGACAAGGACTACAACAAGGTGAAGCTGTACGAG<br>AACGCCGTGGCCAGGTACTCCATGCTGCCCAGCCAGGCCAAGtga	615	204
**AG$_{G167S}$-bN**	AUGGUGAGCGUGAUCAAGCCCGAGAUGAAGAUCAAGCUGUGCAUGAGGGGCACCGUGAACGGCCACAACUUCGUGAUCGAGGGCGAGGGCAAGGG<br>CAACCCCUACGAGGGCACCCAGAUCCUGGACCUGAACGUGACCGAGGGCGCCCCCCUGCCCUUCGCCUACGACAUCCUGACCACCGUGUUCUGCU<br>ACGGCAACAGGGCCUUCACCAAGUACCCCGCCGACAUCCAGGACUACUUCAAGCAGACCUUCCCCGAGGGCUACCACUGGGAGAGGAGCAUGACC<br>UACGAGGACCAGGGCAUCUGCACCGCCACCAGCAACAUCAGCAUGAGGGGCGACUGCUUCUUCUACGACAUCAGGUUCGACGGCACCAACUUCCC<br>CCCCAACGGCCCCGUGAUGCAGAAGAAGACCCUGAAGUGGGAGCCCAGCACCGAGAAGAUGUACGUGGAGGACGGCGUGCUGAAGGGCGACGUGA<br>ACAUGAGGCUGCUGCUGGAGGGCUGCAUCAGCGGCGAUAGCCUGAUCUCUCUGGCCAGCACAGGCAAGAGAGUGCCCAUCAAGGAUCUGCUGGGC<br>GAGAAGGACUUCGAGAUCUGGGCCAUCAACGAGCAGACCAUGAAGCUGGAAAGCGCCAAGGUGUCCCGGGUGUUCUGCACCGGAAAGAAACUGGU<br>GUACACACUGAAAACCCGGCUGGGCAGAACCAUCAAGGCCACCGCCAACCACCGGUUUCUGACAAUCGACGGCUGGAAGAGACUGGACGAGCUGA<br>GCCUGAAAGAGCACAUUGCCCUGCCUAGAAAGCUGGAAUCCAGCAGCCUGCAGuga	816	271
**AG$_{G167S}$-bC**	AUGCUGGCCCCUGAGAUUGAGAAGCUGCCCCAGAGCGACAUCUACUGGGACCCCAUCGUGUCCAUCACCGAGACAGGCGUGGAAGAGGUGUUCGA<br>CCUGACAGUGCCCGGCCUGAGAAACUUCGUGGCCAACGACAUCAUCGUGCACAACAGCGGCCACTACAGGTGCGACTTCAAGACCACCTACAAGG<br>CCAAGAAGGAGGTGAGGCTGCCCGACGCCCACAAGATCGACCACAGGATCGAGATCCTGAAGCACGACAAGGACTACAACAAGGTGAAGCTGTAC<br>GAGAACGCCGTGGCCAGGTACTCCATGCTGCCCAGCCAGGCCAAGtga	333	110
**Npu^{C}-AG^{mid}-Ssp^{N}**	AUGAUCAAGAUCGCCACACGGAAGUACCUGGGCAAGCAGAACGUGUACGACAUCGGCGUGGAACGGGACCACAACUUCGCCCUGAAGAACGGCUU<br>UAUCGCCAGCAACUGCUACGGCAACAGGGCCUUCACCAAGUACCCCGCCGACAUCCAGGACUACUUCAAGCAGACCUUCCCCGAGGGCUACCACU<br>GGGAGAGGAGCAUGACCUACGAGGACCAGGGCAUCUGCACCGCCACCAGCAACAUCAGCAUGAGGGGCGACUGCUUCUUCUACGACAUCAGGUUC<br>GACGGCACCAACUUCCCCCCCAACGGCUGCAUCAGCGGCGAUAGCCUGAUCUCUCUGGCCAGCACAGGCAAGAGAGUGCCCAUCAAGGAUCUGCU<br>GGGCGAGAAGGACUUCGAGAUCUGGGCCAUCAACGAGCAGACCAUGAAGCUGGAAAGCGCCAAGGUGUCCCGGGUGUUCUGCACCGGAAAGAAAC<br>UGGUGUACACACUGAAAACCCGGCUGGGCAGAACCAUCAAGGCCACCGCCAACCACCGGUUUCUGACAAUCGACGGCUGGAAGAGACUGGACGAG<br>CUGAGCCUGAAAGAGCACAUUGCCCUGCCUAGAAAGCUGGAAUCCAGCAGCCUGCAGuga	630	209
**AG^{mid}m-Npu^{N}**	AUGUGCUACGGCAACAGGGCCUUCACCAAGUACCCCGCCGACAUCCAGGACUACUUCAAGCAGACCUUCCCCGAGGGCUACCACUGGGAGAGGAG<br>CAUGACCUACGAGGACCAGGGCAUCUGCACCGCCACCAGCAACAUCAGCAUGAGGGGCGACUGCUUCUUCUACGACAUCAGGUUCGACGGCACCA<br>ACUUCCCCCCCAACGGCUGCCUGAGCUACGAGACAGAGAUCCUGACCGUGGAAUACGGCCUGCUGCCUAUCGGCAAGAUCGUGGAAAAGCGGAUC<br>GAGUGCACCGUGUACUCCGUGGAUAACAACGGCAACAUCUACACCCAGCCUGUGGCUCAGUGGCACGACAGAGGCGAGCAAGAGGUGUUCGAGUA<br>CUGCCUGGAAGAUGGCAGCCUGAUCAGAGCCACCAAGGACCACAAGUUCAUGACAGUGGACGGCCAGAUGCUGCCCAUCGACGAGAUCUUCGAGC<br>GCGAGCUGGACCUGAUGAGAGUGGACAACCUGCCUAACuga	516	171
**Npu^{C}-AG^{mid}m-Ssp^{N}**	AUGAUCAAGAUCGCCACACGGAAGUACCUGGGCAAGCAGAACGUGUACGACAUCGGCGUGGAACGGGACCACAACUUCGCCCUGAAGAACGGCUU<br>UAUCGCCAGCAACUGCUACGGCAACAGGGCCUUCACCAAGUACCCCGCCGACAUCCAGGACUACUUCAAGCAGACCUUCCCCGAGGGCUACCACU<br>GGGAGAGGAGCAUGACCUACGAGGACCAGGGCAUCUGCACCGCCACCAGCAACAUCAGCAUGAGGGGCGACUGCUUCUUCUACGACAUCAGGUUC<br>GACGGCACCAACUUCCCCCCCAACGGCUGCAUCAGCGGCGAUAGCCUGAUCUCUCUGGCCAGCACAGGCAAGAGAGUGCCCAUCAAGGAUCUGCU<br>GGGCGAGAAGGACUUCGAGAUCUGGGCCAUCAACGAGCAGACCAUGAAGCUGGAAAGCGCCAAGGUGUCCCGGGUGUUCUGCACCGGAAAGAAAC<br>UGGUGUACACACUGAAAACCCGGCUGGGCAGAACCAUCAAGGCCACCGCCAACCACCGGUUUCUGACAAUCGACGGCUGGAAGAGACUGGACGAG<br>CUGAGCCUGAAAGAGCACAUUGCCCUGCCUAGAAAGCUGGAAUCCAGCAGCCUGCAGuga	630	209
**Ssp^{C}-AG^{mid}m**	AUGCUGGCCCCUGAGAUUGAGAAGCUGCCCCAGAGCGACAUCUACUGGGACCCCAUCGUGUCCAUCACCGAGACAGGCGUGGAAGAGGUGUUCGA<br>CCUGACAGUGCCCGGCCUGAGAAACUUCGUGGCCAACGACAUCAUCGUGCACAACUGCUACGCCAACAGGGCCUUCACCAAGUACCCCGCCGACA<br>UCCAGGACUACUUCAAGCAGACCUUCCCCGAGGGCUACCACUGGGAGAGGAGCAUGACCUACGAGGACCAGGGCAUCUGCACCGCCACCAGCAAC<br>AUCAGCAUGAGGGGCGACUGCUUCUUCUACGACAUCAGGUUCGACGGCACCAACUUCCCCCCCAACGGCuga	357	118

**5'UTR**			
**5UTR**	aggcgaacuaguauucuucugguccccacagacucagagagaacccgccacc	52	-
**Min5UTR**	aggagacugccacc	14	-
**5UTR-t21**	aggcgaacuaguauucuucuggucucaacaucagucugauaagcuagccacc	52	-
**5UTR-t302a5p**	aggcgaacuaguauucuucuggucagcaaguacauccacguuuaagugccacc	53	-
**5UTR-t206**	aggcgaacuaguauucuucuggucccacacacuuccuuacauuccagccacc	52	-
**3'UTR**			
**mtRNR1-AES 3UTR**	ugaggauccgaucugguacugcaugcacgcaaugcuagcugccccuuucccguccugguaccccgagucucccccgaccucgggucccaggauau gcucccaccuccaccugccccacucaccaccucugcuaguuccagacaccucccaagcacgcagcaaugcagcucaaaacgcuuagccuagccac acccccacgggaaacagcagugauuaaccuuuagcaauaaacgaaaguuuaacuaagcuauacuaaccccaggguuggucaauuucgugccagcc acacc	290	-
**Extra sequence**			
**t21-deaKu5g**	ucaacaucagucugauaagcuaaccuccggaaaccguagauuccgggcggucggagccgccgggagcuguaguucucccgcgccauggugggcuuu uug	98	-
**t302a5p-deaKu5g**	agcaaguacauccacguuuaaguaccuccggaaaccguagauuccgggcggucggagccgccgggagcuguaguucucccgcgccauggugggcuu uuug	99	-
**t206-deaKu5g**	ccacacacuuccuuacauuccaaccuccggaaaccguagauuccgggcggucggagccgccgggagcuguaguucucccgcgccauggugggcuuu uug	98	-
**gRNA**			
**EGFP-targeting**	GGGCACGGGCAGCUUGCCGGguuuuagagcuagaaauagcaaguuaaaauaaggcuaguccguuaucaacuugaaaaaguggcaccgagucggug cuuu	99	-
**DMDex45-targeting**	GGGUAUCUUACAGGAACUCCguuuuagagcuagaaauagcaaguuaaaauaaggcuaguccguuaucaacuugaaaaaguggcaccgagucggug cuuu	99	-

Supplementary Table 2

Description of mRNAs used in this study.

#	Name	Description	CleanCap AG or CleanCap AG 3'Ome	5'UTR	ORF	3'UTR	length of poly(A)	extra sequence
**S1**	**21switch_ctrl**	CC-Min5UTR-hmAG1-3UTR-A100	CC	Min5UTR	hmAG1	3UTR	100	-
**S2**	**21switch_a**	CC-Min5UTR-hmAG1-3UTR-A100-t21-ak	CC	Min5UTR	hmAG1	3UTR	100	t21-ak
**S3**	**21switch_d**	CC-Min5UTR-hmAG1-3UTR-A100-t21-de	CC	Min5UTR	hmAG1	3UTR	100	t21-de
**S4**	**21switch_ad**	CC-Min5UTR-hmAG1-3UTR-A100-t21-ak-de	CC	Min5UTR	hmAG1	3UTR	100	t21-ak-de
**S5**	**21switch_da**	CC-Min5UTR-hmAG1-3UTR-A100-t21-de-ak	CC	Min5UTR	hmAG1	3UTR	100	t21-de-ak
**R**	**iRFP**	CCm-5UTR-iRFP670-3UTR-A120	CCm	5UTR	iRFP670	3UTR	120	-
**1**	**AG**	CC-5UTR-hmAG1-3UTR-A$_{100}$	CC	5UTR	hmAG1	3UTR	100	-
**2**	**AG$_{Q63C}$**	CC-5UTR-hmAG1$_{Q63C}$-3UTR-A$_{100}$	CC	5UTR	hmAG1$_{Q63C}$	3UTR	100	-
**3**	**AG$_{Q63C, G65A}$**	CC-5UTR-hmAG1$_{Q63C, G65A}$-3UTR-A$_{100}$	CC	5UTR	hmAG1$_{Q63C, G65A}$	3UTR	100	-
**4**	**AG^{N}-Int^{N}**	CC-5UTR-AG^{N}-Int^{N}-3UTR-A$_{100}$	CC	5UTR	AG^{N}-Int^{N}	3UTR	100	-
**5**	**Int^{C}-AG^{C}**	CC-5UTR-Int^{C}-AG^{C}-3UTR-A$_{100}$	CC	5UTR	Int^{C}-AG^{C}	3UTR	100	-
**6**	**Int^{C}-AG^{C}m**	CC-5UTR-Int^{C}-AG^{C}m-3UTR-A$_{100}$	CC	5UTR	Int^{C}-AG^{C}m	3UTR	100	-
**11**	**AG$_{Q63C}$-ON$_{21}$**	CC-5UTR-hmAG1$_{Q63C}$-3UTR-A$_{100}$-t21-deaKu5g	CC	5UTR	hmAG1$_{Q63C}$	3UTR	100	t21-deaKu5g
**12**	**AG^{N}-Int^{N}-ON$_{21}$**	CC-5UTR-AG^{N}-Int^{N}-3UTR-A$_{100}$-t21-deaKu5g	CC	5UTR	AG^{N}-Int^{N}	3UTR	100	t21-deaKu5g
**12m**	**AG^{N}-Int^{N}-ON$_{21}$**	CCm-5UTR-AG^{N}-Int^{N}-3UTR-A$_{100}$-t21-deaKu5g	CCm	5UTR	AG^{N}-Int^{N}	3UTR	100	t21-deaKu5g
**13**	**Int^{C}-AG^{C}-ON$_{21}$**	CC-5UTR-Int^{C}-AG^{C}-3UTR-A$_{100}$-t21-deaKu5g	CC	5UTR	Int^{C}-AG^{C}	3UTR	100	t21-deaKu5g
**14**	**Int^{C}-AG^{C}m-OFF$_{21}$**	CC-5UTR-t21-Int^{C}-AG^{C}m-3UTR-A$_{120}$	CC	5UTR-t21	Int^{C}-AG^{C}m	3UTR	120	-
**14m**	**Int^{C}-AG^{C}m-OFF$_{21}$**	CCm-5UTR-t21-Int^{C}-AG^{C}m-3UTR-A$_{120}$	CCm	5UTR-t21	Int^{C}-AG^{C}m	3UTR	120	-
**15**	**PAC**	CCm-5UTR-PAC-3UTR-A$_{120}$	CCm	5UTR	PAC	3UTR	120	-
**18**	**PAC-ON$_{21}$**	CCm-5UTR-PAC-3UTR-A$_{100}$-t21-deaKu5g	CCm	5UTR	PAC	3UTR	100	t21-deaKu5g
**21**	**PAC^{N}-Int^{N}-ON$_{21}$**	CCm-5UTR-PAC^{N}-Int^{N}-3UTR-A$_{100}$-t21-deaKu5g	CCm	5UTR	PAC^{N}-Int^{N}	3UTR	100	t21-deaKu5g
**22**	**Int^{C}-PAC^{C}-ON$_{21}$**	CCm-5UTR-Int^{C}-PAC^{C}-3UTR-A$_{100}$-t21-deaKu5g	CCm	5UTR	Int^{C}-PAC^{C}	3UTR	100	t21-deaKu5g
**24**	**Int^{C}-PAC^{C}m-OFF$_{21}$**	CCm-5UTR-t21-Int^{C}-PAC^{C}m-3UTR-A$_{120}$	CCm	5UTR-t21	Int^{C}-PAC^{C}m	3UTR	120	-
**26**	**RF$_{A118C}$**	CC-5UTR-iRFP670$_{A118C}$-3UTR-A$_{100}$	CC	5UTR	iRFP670$_{A118C}$	3UTR	100	-
**27**	**RF^{N}-Int^{N}**	CC-5UTR-iRFP^{N}-Int^{N}-3UTR-A$_{100}$	CC	5UTR	RF^{N}-Int^{N}	3UTR	100	-
**28**	**Int^{C}-RF^{C}**	CC-5UTR-Int^{C}-iRFP^{C}-3UTR-A$_{100}$	CC	5UTR	Int^{C}-RF^{C}	3UTR	100	-
**29**	**RF$_{A118C}$-OFF$_{21}$**	CC-5UTR-t21-iRFP670$_{A118C}$-3UTR-A$_{120}$	CC	5UTR-t21	iRFP670$_{A118C}$	3UTR	120	-
**30**	**RF^{N}-Int^{N}-OFF$_{21}$**	CC-5UTR-t21-iRFP^{N}-Int^{N}-3UTR-A$_{120}$	CC	5UTR-t21	RF^{N}-Int^{N}	3UTR	120	-
**31**	**Int^{C}-RF^{C}-OFF$_{21}$**	CC-5UTR-t21-Int^{C}-iRFP^{C}-3UTR-A$_{120}$	CC	5UTR-t21	Int^{C}-RF^{C}	3UTR	120	-
**32**	**PAC-ON$_{302a}$**	CCm-5UTR-PAC-3UTR-A$_{100}$-t302a5p-deaKu5g	CCm	5UTR	PAC	3UTR	100	-
**33**	**PAC^{N}-Int^{N}-ON$_{302a}$**	CCm-5UTR-PAC^{N}-Int^{N}-3UTR-A$_{100}$-t302a5p-deaKu5g	CCm	5UTR	PAC^{N}-Int^{N}	3UTR	100	t302a5p-deaKu5g
**34**	**Int^{C}-PAC^{C}-ON$_{302a}$**	CCm-5UTR-Int^{C}-PAC^{C}-3UTR-A$_{100}$-t302a5p-deaKu5g	CCm	5UTR	Int^{C}-PAC^{C}	3UTR	100	t302a5p-deaKu5g
**35**	**Int^{C}-PAC^{C}m-OFF$_{302a}$**	CCm-5UTR-t302a5p-Int^{C}-PAC^{C}m-3UTR-A$_{120}$	CCm	5UTR-t302a5p	Int^{C}-PAC^{C}m	3UTR	120	-
**36**	**RF-ON$_{302a}$**	CC-5UTR-iRFP670-3UTR-A$_{100}$-t302a5p-deaKu5g	CC	5UTR	iRFP670$_{A118C}$	3UTR	100	t302a5p-deaKu5g
**37**	**Int^{C}-AG^{C}-ON$_{302a}$**	CC-5UTR-Int^{C}-AG^{C}-3UTR-A$_{100}$-t302a5p-deaKu5g	CC	5UTR	Int^{C}-AG^{C}	3UTR	100	t302a5p-deaKu5g
**38**	**Int^{C}-AG^{C}m-OFF$_{302a}$**	CC-5UTR-t302a5p-Int^{C}-AG^{C}m-3UTR-A$_{120}$	CC	5UTR-t302a5p	Int^{C}-AG^{C}m	3UTR	120	-
**38m**	**Int^{C}-AG^{C}m-OFF$_{302a}$**	CCm-5UTR-t302a5p-Int^{C}-AG^{C}m-3UTR-A$_{120}$	CCm	5UTR-t302a5p	Int^{C}-AG^{C}m	3UTR	120	-
**39**	**RF^{N}-Int^{N}-OFF$_{302a}$**	CC-5UTR-t302a5p-iRFP^{N}-Int^{N}-3UTR-A$_{120}$	CC	5UTR-t302a5p	RF^{N}-Int^{N}	3UTR	120	-
**40**	**AG^{N}-Int^{N}-OFF$_{21}$**	CC-5UTR-t21-AG^{N}-Int^{N}-3UTR-A$_{120}$	CC	5UTR-t21	AG^{N}-Int^{N}	3UTR	120	-
**40m**	**AG^{N}-Int^{N}-OFF$_{21}$**	CCm-5UTR-t21-AG^{N}-Int^{N}-3UTR-A$_{120}$	CCm	5UTR-t21	AG^{N}-Int^{N}	3UTR	120	-
**41**	**Int^{C}-AG^{C}-OFF$_{21}$**	CC-5UTR-t21-Int^{C}-AG^{C}-3UTR-A$_{120}$	CC	5UTR-t21	Int^{C}-AG^{C}	3UTR	120	-
**41m**	**Int^{C}-AG^{C}-OFF$_{21}$**	CCm-5UTR-t21-Int^{C}-AG^{C}-3UTR-A$_{120}$	CCm	5UTR-t21	Int^{C}-AG^{C}	3UTR	120	-
**42**	**Int^{C}-AG^{C}-OFF$_{302a}$**	CC-5UTR-t302a5p-Int^{C}-AG^{C}m-3UTR-A$_{120}$	CC	5UTR-t302a5p	Int^{C}-AG^{C}	3UTR	120	-
**42m**	**Int^{C}-AG^{C}-OFF$_{302a}$**	CCm-5UTR-t302a5p-Int^{C}-AG^{C}m-3UTR-A$_{120}$	CCm	5UTR-t302a5p	Int^{C}-AG^{C}	3UTR	120	-
**43**	**AG^{N}-Int^{N}-ON$_{302a}$**	CCm-5UTR-AG^{N}-Int^{N}-3UTR-A$_{100}$-t302a5p-deaKu5g	CCm	5UTR	AG^{N}-Int^{N}	3UTR	100	t302a5p-deaKu5g
**44**	**TK$_{A266C}$**	CCm-5UTR-TK$_{A266C}$-3UTR-A$_{120}$	CCm	5UTR	TK$_{A266C}$	3UTR	120	-

45	**TK^{N}-Int^{N}**	CCm-5UTR-TK^{N}-Int^{N}-3UTR-A$_{120}$	CCm	5UTR	TK^{N}-Int^{N}	3UTR	120	-
46	**Int^{C}-TK^{C}**	CCm-5UTR- Int^{C}-TK^{C}-3UTR-A$_{120}$	CCm	5UTR	Int^{C}-TK^{C}	3UTR	120	-
47	**TK$_{A266C}$-ON$_{21}$**	CCm-5UTR-TK$_{A266C}$-3UTR-A$_{100}$-t21-deaKu5g	CCm	5UTR	TK$_{A266C}$	3UTR	100	t21-deaKu5g
48	**TK^{N}-Int^{N}-ON$_{21}$**	CCm-5UTR-TK^{N}-Int^{N}-3UTR-A$_{100}$-t21-deaKu5g	CCm	5UTR	TK^{N}-Int^{N}	3UTR	100	t21-deaKu5g
49	**Int^{C}-TK^{C}-ON$_{21}$**	CCm-5UTR- Int^{C}-TK^{C}-3UTR-A$_{100}$-t21-deaKu5g	CCm	5UTR	Int^{C}-TK^{C}	3UTR	100	t21-deaKu5g
50	**HPH**	CCm-5UTR-HPH-3UTR-A$_{120}$	CCm	5UTR	HPH	3UTR	120	-
51	**HPH^{N}-Int^{N}**	CCm-5UTR-HPH^{N}-Int^{N}-3UTR-A$_{120}$	CCm	5UTR	HPH^{N}-Int^{N}	3UTR	120	-
52	**Int^{C}-HPH^{C}**	CCm-5UTR- Int^{C}-HPH^{C}-3UTR-A$_{120}$	CCm	5UTR	Int^{C}-HPH^{C}	3UTR	120	-
53	**BSR**	CCm-5UTR-BSR-3UTR-A$_{120}$	CCm	5UTR	BSR	3UTR	120	-
54	**BSR^{N}-Int^{N}**	CCm-5UTR-BSR^{N}-Int^{N}-3UTR-A$_{120}$	CCm	5UTR	BSR^{N}-Int^{N}	3UTR	120	-
55	**Int^{C}-BSR^{C}**	CCm-5UTR- Int^{C}-BSR^{C}-3UTR-A$_{120}$	CCm	5UTR	Int^{C}-BSR^{C}	3UTR	120	-
56	**HPH-ON$_{21}$**	CCm-5UTR-HPH-3UTR-A$_{100}$-t21-deaKu5g	CCm	5UTR	HPH	3UTR	100	t21-deaKu5g
57	**HPH^{N}-Int^{N}-ON$_{21}$**	CCm-5UTR-HPH^{N}-Int^{N}-3UTR-A$_{100}$-t21-deaKu5g	CCm	5UTR	HPH^{N}-Int^{N}	3UTR	100	t21-deaKu5g
58	**Int^{C}-HPH^{C}-ON$_{21}$**	CCm-5UTR- Int^{C}-HPH^{C}-3UTR-A$_{100}$-t21-deaKu5g	CCm	5UTR	Int^{C}-HPH^{C}	3UTR	100	t21-deaKu5g
59	**BSR-ON$_{21}$**	CCm-5UTR-BSR-3UTR-A$_{100}$-t21-deaKu5g	CCm	5UTR	BSR	3UTR	100	t21-deaKu5g
60	**BSR^{N}-Int^{N}-ON$_{21}$**	CCm-5UTR-BSR^{N}-Int^{N}-3UTR-A$_{100}$-t21-deaKu5g	CCm	5UTR	BSR^{N}-Int^{N}	3UTR	100	t21-deaKu5g
61	**Int^{C}-BSR^{C}-ON$_{21}$**	CCm-5UTR- Int^{C}-BSR^{C}-3UTR-A$_{100}$-t21-deaKu5g	CCm	5UTR	Int^{C}-BSR^{C}	3UTR	100	t21-deaKu5g
63	**spCas9$_{both}$**	CCm-Kit5UTR-spCas9$_{both}$-Kit3UTR-A$_{120}$	CCm	5UTR	spCas9$_{both}$	3UTR	120	-
65	**NLS-spCas9^{N}-Int^{N}**	CCm-Kit5UTR-NLS-spCas9^{N}-Int^{N}-Kit3UTR-A$_{120}$	CCm	5UTR	NLS-spCas9^{N}-Int^{N}	3UTR	120	-
66	**Int^{C}-spCas9^{C}**	CCm-Kit5UTR- Int^{C}-spCas9^{C}-Kit3UTR-A$_{120}$	CCm	5UTR	Int^{C}-spCas9^{C}	3UTR	120	-
67	**AG-OFF$_{L7Ae}$**	CCm-Kt-Kit5UTR-hmAG1$_{Q63C}$-Kit3UTR-A$_{120}$	CCm	5UTR	hmAG1$_{Q63C}$	3UTR	120	-
68	**AG^{N}-Int^{N}-OFF$_{L7Ae}$**	CCm-Kt-Kit5UTR-AG^{N}-Int^{N}-Kit3UTR-A$_{120}$	CCm	5UTR	AG^{N}-Int^{N}	3UTR	120	-
69	**L7Ae**	CCm-Kit5UTR-L7Ae-Kit3UTR-A$_{120}$	CCm	5UTR	L7Ae	3UTR	120	-
70	**AG-OFF$_{LIN28A}$**	CCm-stbC-Kit5UTR-hmAG1$_{Q63C}$-Kit3UTR-A$_{120}$	CCm	5UTR	hmAG1$_{Q63C}$	3UTR	120	-
71	**AG^{N}-Int^{N}-OFF$_{LIN28A}$**	CCm-stbC-Kit5UTR-AG^{N}-Int^{N}-Kit3UTR-A$_{120}$	CCm	5UTR	AG^{N}-Int^{N}	3UTR	120	-
72	**LIN28A**	CCm-Kit5UTR-LIN28A-Kit3UTR-A$_{120}$	CCm	5UTR	LIN28A	3UTR	120	-
73	**AG$_{Q63C}$_v2**	CCm-Kit5UTR-hmAG1$_{Q63C}$-Kit3UTR-A$_{120}$	CCm	5UTR	hmAG1$_{Q63C}$	3UTR	120	-
74	**RF$_{A118C}$_v2**	CCm-Kit5UTR-iRFP670$_{A118C}$-Kit3UTR-A$_{120}$	CCm	5UTR	iRFP670$_{A118C}$	3UTR	120	-
75	**AG$_{Q63C}$ -OFF$_{302a}$**	CCm-Kit5UTR-t302a5p-hmAG1$_{Q63C}$-Kit3UTR-A$_{120}$	CCm	5UTR-t302a5p	hmAG1$_{Q63C}$	3UTR	120	-
76	**spCas9$_{both}$-ON$_{21}$**	CCm-Kit5UTR-spCas9$_{both}$-Kit3UTR-A$_{100}$-t21-deaKu5g	CCm	5UTR	spCas9$_{both}$	3UTR	100	t21-deaKu5g
77	**NLS-spCas9^{N}-Int^{N}-ON$_{21}$**	CCm-Kit5UTR-NLS-spCas9^{N}-Int^{N}-Kit3UTR-A$_{100}$-t21-deaKu5g	CCm	5UTR	NLS-spCas9^{N}-Int^{N}	3UTR	100	t21-deaKu5g
78	**Int^{C}-spCas9^{C}-NLS-ON$_{21}$**	CCm-Kit5UTR-Int^{C}-spCas9^{C}-NLS-Kit3UTR-A$_{100}$-t21-deaKu5g	CCm	5UTR	Int^{C}-spCas9^{C}	3UTR	100	t21-deaKu5g
79	**AG$_{Q63C, P131S}$**	CCm-Kit5UTR- AG$_{Q63C, P131S}$ -Kit3UTR-A$_{120}$	CCm	5UTR	AG$_{Q63C, P131S}$	3UTR	120	-
80	**AG$_{Q63C, V153S}$**	CCm-Kit5UTR- AG$_{Q63C, V153S}$ -Kit3UTR-A$_{120}$	CCm	5UTR	AG$_{Q63C, V153S}$	3UTR	120	-
81	**AG$_{Q63C, G167S}$**	CCm-Kit5UTR- AG$_{Q63C, G167S}$ -Kit3UTR-A$_{120}$	CCm	5UTR	AG$_{Q63C, G167S}$	3UTR	120	-
82	**AG$_{P131S}$-aN**	CCm-Kit5UTR-AG$_{P131S}$-aN-Kit3UTR-A$_{120}$	CCm	5UTR	AG$_{P131S}$-aN	3UTR	120	-
83	**AG$_{P131S}$-aC**	CCm-Kit5UTR-AG$_{P131S}$-aC-Kit3UTR-A$_{120}$	CCm	5UTR	AG$_{P131S}$-aC	3UTR	120	-
84	**AG$_{P131S}$-bN**	CCm-Kit5UTR-AG$_{P131S}$-bN-Kit3UTR-A$_{120}$	CCm	5UTR	AG$_{P131S}$-bN	3UTR	120	-
85	**AG$_{P131S}$-bC**	CCm-Kit5UTR-AG$_{P131S}$-bC-Kit3UTR-A$_{120}$	CCm	5UTR	AG$_{P131S}$-bC	3UTR	120	-
86	**AG$_{V153S}$-aN**	CCm-Kit5UTR-AG$_{V153S}$-aN-Kit3UTR-A$_{120}$	CCm	5UTR	AG$_{V153S}$-aN	3UTR	120	-
87	**AG$_{V153S}$-aC**	CCm-Kit5UTR-AG$_{V153S}$-aC-Kit3UTR-A$_{120}$	CCm	5UTR	AG$_{V153S}$-aC	3UTR	120	-
88	**AG$_{V153S}$-bN**	CCm-Kit5UTR-AG$_{V153S}$-bN-Kit3UTR-A$_{120}$	CCm	5UTR	AG$_{V153S}$-bN	3UTR	120	-
89	**AG$_{V153S}$-bC**	CCm-Kit5UTR-AG$_{V153S}$-bC-Kit3UTR-A$_{120}$	CCm	5UTR	AG$_{V153S}$-bC	3UTR	120	-
90	**AG$_{G167S}$-aN**	CCm-Kit5UTR-AG$_{G167S}$-aN-Kit3UTR-A$_{120}$	CCm	5UTR	AG$_{G167S}$-aN	3UTR	120	-
91	**AG$_{G167S}$-aC**	CCm-Kit5UTR-AG$_{G167S}$-aC-Kit3UTR-A$_{120}$	CCm	5UTR	AG$_{G167S}$-aC	3UTR	120	-
92	**AG$_{G167S}$-bN**	CCm-Kit5UTR-AG$_{G167S}$-bN-Kit3UTR-A$_{120}$	CCm	5UTR	AG$_{G167S}$-bN	3UTR	120	-
93	**AG$_{G167S}$-bC**	CCm-Kit5UTR-AG$_{G167S}$-bC-Kit3UTR-A$_{120}$	CCm	5UTR	AG$_{G167S}$-bC	3UTR	120	-
96	**spCas9$_{both}$-ON$_{302a}$**	CCm-Kit5UTR-spCas9$_{both}$-Kit3UTR-A$_{100}$-t302a-deaKu5g	CCm	5UTR	spCas9$_{both}$	3UTR	100	t302a5p-deaKu5g
97	**NLS-spCas9^{N}-Int^{N}-ON$_{302a}$**	CCm-Kit5UTR-NLS-spCas9^{N}-Int^{N}-Kit3UTR-A$_{100}$-t302a-deaKu5g	CCm	5UTR	NLS-spCas9^{N}-Int^{N}	3UTR	100	t302a5p-deaKu5g
98	**Int^{C}-spCas9^{C}-NLS-ON$_{302a}$**	CCm-Kit5UTR-Int^{C}-spCas9^{C}-NLS-Kit3UTR-A$_{100}$-t302a-deaKu5g	CCm	5UTR	Int^{C}-spCas9^{C}-NLS	3UTR	100	t302a5p-deaKu5g

**99**	**Npu^{C}-AG^{mid}-Ssp^{N}**	CCm-Kit5UTR-Npu^{C}-AG^{mid}-Ssp^{N}-Kit3UTR-A_{120}	CCm	5UTR	Npu^{C}-AG^{mid}-Ssp^{N}	3UTR	120	-
**100**	**Npu^{C}-AG^{mid}-Ssp^{N}-OFF$_{302a}$**	CCm-5UTR-t302a5p-Npu^{C}-AG^{mid}-Ssp^{N}-3UTR-A_{120}	CCm	5UTR-t302a5p	Npu^{C}-AG^{mid}-Ssp^{N}	3UTR	120	-
**101**	**AG$_{P131S}$-bC-OFF$_{206}$**	CCm-5UTR-t206-AG$_{P131S}$-bC-3UTR-A_{120}	CCm	5UTR-t206	AG$_{P131S}$-bC	3UTR	120	-
**102**	**AG^{N}-Int^{N}**	CCm-5UTR-AG^{N}-Int^{N}-3UTR-A_{120}	CCm	5UTR	AG^{N}-Int^{N}	3UTR	120	-
**103**	**Npu^{C}-AG^{mid}-Ssp^{N}-ON$_{302a}$**	CCm-5UTR-Npu^{C}-AG^{mid}-Ssp^{N}-3UTR-A_{100}-t302a5p-deaKu5g	CCm	5UTR	Npu^{C}-AG^{mid}-Ssp^{N}	3UTR	100	t302a5p-deaKu5g
**104**	**AG$_{P131S}$-bC-ON$_{206}$**	CCm-5UTR-AG$_{P131S}$-bC-3UTR-A_{100}-t206-deaKu5g	CCm	5UTR	AG$_{P131S}$-bC	3UTR	100	t206-deaKu5g
**105**	**AG^{mid}m-Npu^{N}-OFF$_{21}$**	CCm-5UTR-t21-AG^{mid}m-Npu^{N}-3UTR-A_{120}	CCm	5UTR-t21	AG^{mid}m-Npu^{N}	3UTR	120	-
**106**	**Npu^{C}-AG^{mid}m-Ssp^{N}-OFF$_{302a}$**	CCm-5UTR-t302a5p-Npu^{C}-AG^{mid}m-Ssp^{N}-3UTR-A_{120}	CCm	5UTR-t302a5p	Npu^{C}-AG^{mid}m-Ssp^{N}	3UTR	120	-
**107**	**Ssp^{C}-AG^{mid}m-OFF$_{206}$**	CCm-5UTR-t206-Ssp^{C}-AG^{mid}m-3UTR-A_{120}	CCm	5UTR-t206	Ssp^{C}-AG^{mid}m	3UTR	120	-

Supplementary Table 3

Detailed information on the conditions for cell experiments.

Fig.	Experiment	Plate type	Medium volume (μl)	Cell type	Cell number per well	miRNA mimic or Inhibitor	Conc. (nM)	mRNA	Volume (ng)	Antibiotics
**2b, c, d**	**split-AG ON switch**	24 well plate	500	293FT	75,000	miR-21-5p mimic<br>NC mimic	4 nM<br>4 nM	11: AG$_{Q63C}$-ON$_{21}$<br>12: AG^{N}-Int^{N}-ON$_{21}$<br>13: Int^{C}-AG^{C}-ON$_{21}$<br>14: Int^{C}-AG^{C}m-OFF$_{21}$<br>26: RF$_{A118C}$ (internal control)	50<br>50<br>50<br>50<br>x1 = 50, x4 = 200	-
**3b**	**split-PAC ON switch**	96 well plate	100	HeLa	10,000	miR-21-5p inhibitor<br>NC inhibitor	4 nM<br>4 nM	15: PAC<br>18: PAC-ON$_{21}$<br>21: PAC^{N}-Int^{N}-ON$_{21}$<br>22: Int^{C}-PAC^{C}-ON$_{21}$<br>24: Int^{C}-PAC^{C}m-OFF$_{21}$	25<br>25<br>25<br>25<br>100	Puromycin: 2 μg/ml
**3d**	**split-HPH ON switch**	96 well plate	100	HeLa	10,000	miR-21-5p inhibitor<br>NC inhibitor	4 nM<br>4 nM	24: Int^{C}-PAC^{C}m-OFF$_{21}$<br>50: HPH<br>56: HPH-ON$_{21}$<br>57: HPH^{N}-Int^{N}-ON$_{21}$<br>58: Int^{C}-HPH^{C}-ON$_{21}$	100<br>25<br>25<br>25<br>25	Hygromycin B: 500 μg/ml
**3f**	**split-BSR ON switch**	96 well plate	100	HeLa	10,000	miR-21-5p inhibitor<br>NC inhibitor	4 nM<br>4 nM	24: Int^{C}-PAC^{C}m-OFF$_{21}$<br>53: BSR<br>59: BSR-ON$_{21}$<br>60: BSR^{N}-Int^{N}-ON$_{21}$<br>61: Int^{C}-BSR^{C}-ON$_{21}$	100<br>25<br>25<br>25<br>25	Blasticidin S: 100 μg/ml
**3h**	**split-TK ON switch**	96 wel plate	100	HeLa	10,000	miR-21-5p inhibitor<br>NC inhibitor	4 nM<br>4 nM	14: Int^{C}-AG^{C}m-OFF$_{21}$<br>44: TK$_{A266C}$<br>47: TK$_{A266C}$-ON$_{21}$<br>48: TK^{N}-Int^{N}-ON$_{21}$<br>49: Int^{C}-TK^{C}-ON$_{21}$	100<br>25<br>25<br>25<br>25	Ganciclovir: 10 μg/ml
**4c, d**	**Hela selection from the mixture with 293FT**	96 well plate	100	HeLa$_{hmAG1-M9}$<br>HEK293FT$_{iRFP670-M9}$	10,000<br>2,000			14: Int^{C}-AG^{C}m-OFF$_{21}$<br>53: BSR<br>59: BSR-ON$_{21}$<br>60: BSR^{N}-Int^{N}-ON$_{21}$<br>61: Int^{C}-BSR^{C}-ON$_{21}$	200<br>25<br>25<br>25<br>25	Blasticidin S: 100 μg/ml
**5b**	**miR-21-5p-responsive split-Cas9 ON switch in HeLaEGFP ± miR-21-5p**	24 well plate	500	HeLa$_{EGFP}$	20,000	miR-21-5p inhibitor<br>NC inhibitor	4 nM<br>4 nM	14: Int^{C}-AG^{C}m-OFF$_{21}$<br>63: spCas9$_{both}$<br>76: spCas9$_{both}$-ON$_{21}$<br>77: NLS-spCas9^{N}-Int^{N}-ON$_{21}$<br>78: Int^{C}-spCas9^{C}-NLS-ON$_{21}$	x3 = 300, x6 = 600<br>100<br>100<br>100<br>100	-
**5c**	**miR-302a-5p-responsive split-Cas9 ON switch in hiPSCEGFP ± miR-302a-5p**	24 well plate	500	hiPSC$_{EGFP}$	20,000	miR-302a-5p inhibitor<br>NC inhibitor	4 nM<br>4 nM	38m: Int^{C}-AG^{C}m-OFF$_{302a}$<br>63: spCas9$_{both}$<br>96: spCas9$_{both}$-ON$_{302a}$<br>97: NLS-spCas9^{N}-Int^{N}-ON$_{302a}$<br>98: Int^{C}-spCas9^{C}-NLS-ON$_{302a}$	x3 = 300, x6 = 600<br>100<br>100<br>100<br>100	-
**5e**	**DMDex45 editing by miR-21-5p-responsive split-Cas9 ON switch in HEK293TEGFP ± miR-21a-5p**	24 well plate	500	HEK293T$_{EGxxFP}$	40,000	miR-21-5p inhibitor<br>miR-21-5p mimic	4 nM<br>4 nM	14m: Int^{C}-AG^{C}m-OFF$_{21}$<br>63: spCas9$_{both}$<br>76: spCas9$_{both}$-ON$_{21}$<br>77: NLS-spCas9^{N}-Int^{N}-ON$_{21}$<br>78: Int^{C}-spCas9^{C}-NLS-ON$_{21}$	x3 = 300, x6 = 600<br>100<br>100<br>100<br>100	-
**6b, c, d**	**Toggle-like system**	24 well plate	500	293FT	75,000	miR-21-5p mimic	titrated as	11: AG$_{Q63C}$-ON$_{21}$<br>12: AG^{N}-Int^{N}-ON$_{21}$	100<br>100	-

							shown in figure	13: Int^{C}-AG^{C}-ON$_{21}$	100	
								29: RF$_{A118C}$-OFF$_{21}$	100	
								30: RF^{N}-Int^{N}-OFF$_{21}$	100	
								31: Int^{C}-RF^{C}-OFF$_{21}$	100	
7b	**2-input system [00]**	24 well plate	500	293FT	75,000	miR-21-5p mimic	4 nM	12: AG^{N}-Int^{N}-ON$_{21}$	100	-
						miR-302a-5p mimic	4 nM	14: Int^{C}-AG^{C}m-OFF$_{21}$	300	
								26: RF$_{A118C}$ (internal control)	100	
								37: Int^{C}-AG^{C}-ON$_{302a}$	200	
								39: RF^{N}-Int^{N}-OFF$_{302a}$	300	
	**2-input system [10]**	24 well plate	500	293FT	75,000	miR-21-5p mimic	4 nM	26: RF$_{A118C}$ (internal control)	100	-
						miR-302a-5p mimic	4 nM	40: AG^{N}-Int^{N}-OFF$_{21}$	100	
								42: Int^{C}-AG^{C}-OFF$_{302a}$	100	
	**2-input system [01]**	24 well plate	500	293FT	75,000	miR-21-5p mimic	4 nM	12: AG^{N}-Int^{N}-ON$_{21}$	100	-
						miR-302a-5p mimic	4 nM	14: Int^{C}-AG^{C}m-OFF$_{21}$	300	
								26: RF$_{A118C}$ (internal control)	100	
								42: Int^{C}-AG^{C}-OFF$_{302a}$	100	
	**2-input system [01]**	24 well plate	500	293FT	75,000	miR-21-5p mimic	4 nM	26: RF$_{A118C}$ (internal control)	100	-
						miR-302a-5p mimic	4 nM	38: Int^{C}-AG^{C}m-OFF$_{302a}$	300	
								41: Int^{C}-AG^{C}-OFF$_{21}$	100	
								43: AG^{N}-Int^{N}-ON$_{302a}$	200	
8b	**miR-21/L7Ae-NOR gate**	24 well plate	500	293FT	75,000	miR-21-5p mimic	4 nM	26: RF$_{A118C}$ (internal control)	100	-
								41m: Int^{C}-AG^{C}-OFF$_{21}$	100	
								68: AG^{N}-Int^{N}-OFF$_{L7Ae}$	100	
								69: L7Ae	20	
								73: AG$_{Q63C}$_v2	100	
	**miR-302a/LIN28A-NOR gate**	24 well plate	500	293FT	75,000	miR-302a-5p mimic	4 nM	26: RF$_{A118C}$ (internal control)	100	-
								42m: Int^{C}-AG^{C}-OFF$_{302a}$	100	
								71: AG^{N}-Int^{N}-OFF$_{LIN28A}$	200	
								72: LIN28A	600	
								73: AG$_{Q63C}$_v2	100	
8c	**miR-302a/LIN28A-NOR gate in iPSC**	24 well plate	500	293FT	75,000			42m: Int^{C}-AG^{C}-OFF$_{302a}$	100	-
				hiPSC	75,000			70: AG-OFF$_{LIN28A}$	200	
								71: AG^{N}-Int^{N}-OFF$_{LIN28A}$	200	
								74: RF$_{A118C}$_v2	100	
								75: AG$_{Q63C}$ -OFF$_{302a}$	100	
8e	**miR-21/302a/206 NOR gate**	24 well plate	500	293FT	75,000	miR-21-5p mimic	4 nM	26: RF$_{A118C}$ (internal control)	50	-
						miR-302a-5p mimic	4 nM	40m: AG^{N}-Int^{N}-OFF$_{21}$	50	
						miR-206 mimic	4 nM	100: Npu^{C}-AG^{mid}-Ssp^{N}-OFF$_{302a}$	50	
								101: AG$_{P131S}$-bC-OFF$_{206}$	50	
	**miR-21/302a/206 AND gate**	24 well plate	500	293FT	75,000	miR-21-5p mimic	4 nM	12m: AG^{N}-Int^{N}-ON$_{21}$	50	-
						miR-302a-5p mimic	4 nM	26: RF$_{A118C}$ (internal control)	50	
						miR-206 mimic	4 nM	103: Npu^{C}-AG^{mid}-Ssp^{N}-ON$_{302a}$	50	
								104: AG$_{P131S}$-bC-ON$_{206}$	50	
								105: AG^{mid}m-Npu^{N}-OFF$_{21}$	150	
								106: Npu^{C}-AG^{mid}m-Ssp^{N}-OFF$_{302a}$	150	
								107: Ssp^{C}-AG^{mid}m-OFF$_{206}$	600	
S2	**investigation of extra sequence**	24 well plate	500	293FT	100,000	miR-21-5p mimic	4 nM	S1: 21switch_ctrl	100	-
						NC mimic	4 nM	S2: 21switch_a	100	
								S3: 21switch_d	100	
								S4: 21switch_ad	100	
								S5: 21switch_da	100	
								R: iRFP	100	
**S3**			500	293FT	100,000			1: AG$_{Q63C}$	100	-

	**Evaluation of protein splicing**	24 well plate						4: AG^{N}-Int^{N}	100	
								5: Int^{C}-AG^{C}	100	
								26: RF_{A118C} (internal control)	100	
**S6**	**Split-PAC iPSC/Hela**	96 well plate	100	hiPSC	20,000			15: PAC	50	Puromycin: 2 µg/ml
				HeLa	10,000			32: PAC-ON_{302a}	50	
								33: PAC^{N}-Int^{N}-ON_{302a}	50	
								34: Int^{C}-PAC^{C}-ON_{302a}	50	
								35: Int^{C}-PAC^{C}m-OFF_{302a}	x2 = 100, x4 = 200	
**S11 b**	**Evaluation of split-sites of hmAG1 and SspDnaB intein for the construction of three-input logic circuits**	24 well plate	500	293FT	75,000			26: RF_{A118C} (internal control)	50	-
								73: AG_{Q63C}_v2	50	
								79: $AG_{Q63C,\ P131S}$	50	
								80: $AG_{Q63C,\ V153S}$	50	
								81: $AG_{Q63C,\ G167S}$	50	
								82: AG_{P131S}-aN	50	
								83: AG_{P131S}-aC	50	
								84: AG_{P131S}-bN	50	
								85: AG_{P131S}-bC	50	
								86: AG_{V153S}-aN	50	
								87: AG_{V153S}-aC	50	
								88: AG_{V153S}-bN	50	
								89: AG_{V153S}-bC	50	
								90: AG_{G167S}-aN	50	
								91: AG_{G167S}-aC	50	
								92: AG_{G167S}-bN	50	
								93: AG_{G167S}-bC	50	
**S12**	**Evaluation of cytotoxicity of split-intein and mutated protein fragments in HEK293FT cell**	98 well plate	100	HeLa	5,000			1: AG	25	-
								2: AG_{Q63C}	25	
								3: $AG_{Q63C,\ G65A}$	25	
								4: AG^{N}-Int^{N}	25	
								5: Int^{C}-AG^{C}	25	
								6: Int^{C}-AG^{C}m	25	

Supplementary Table 4

Statistics and *P*-values.

Figure	Name	Condition #1	Condition #2	Value	Statistical analysis	*P*-value
Fig.2b	-	AGQ63C-ON21	AGN-IntN-ON21<br>IntC-AGC-ON21	ON/OFF ratio of relative hmAG1 intensity [a.u.]	Two-sided Welch's t-test	0.00999
	-	AGN-IntN-ON21<br>IntC-AGC-ON21	AGN-IntN-ON21<br>IntC-AGC-ON21<br>IntC-AGCm-OFF21 x1	ON/OFF ratio of relative hmAG2 intensity [a.u.]	Two-sided Welch's t-test	0.00270
	-	AGN-IntN-ON21<br>IntC-AGC-ON21<br>IntC-AGCm-OFF21 x1	AGN-IntN-ON21<br>IntC-AGC-ON21<br>IntC-AGCm-OFF21 x4	ON/OFF ratio of relative hmAG3 intensity [a.u.]	Two-sided Welch's t-test	0.00179
	-	AGN-IntN-ON21<br>IntC-AGC-ON21	AGN-IntN-ON21<br>IntC-AGC-ON21<br>IntC-AGCm-OFF21 x4	ON/OFF ratio of relative hmAG4 intensity [a.u.]	Two-sided Welch's t-test	0.00122
Fig.3b	Mock	NC inhibitor	miR-21-5p inhibitor	Survival rate (Puro+/Puro-) [%]	Two-sided Welch's t-test	0.39970
	PAC mRNA	NC inhibitor	miR-21-5p inhibitor	Survival rate (Puro+/Puro-) [%]	Two-sided Welch's t-test	0.38825
	PAC-ON21	NC inhibitor	miR-21-5p inhibitor	Survival rate (Puro+/Puro-) [%]	Two-sided Welch's t-test	0.02450
	PACN-IntN-ON21<br>IntC-PACC-ON21<br>IntC-PACCm-OFF21	NC inhibitor	miR-21-5p inhibitor	Survival rate (Puro+/Puro-) [%]	Two-sided Welch's t-test	4.38E-05
Fig.3d	Mock	NC inhibitor	miR-21-5p inhibitor	Survival rate (Hygro+/Hygro-) [%]	Two-sided Welch's t-test	0.71901
	HPH mRNA	NC inhibitor	miR-21-5p inhibitor	Survival rate (Hygro+/Hygro-) [%]	Two-sided Welch's t-test	0.61318
	HPH-ON21	NC inhibitor	miR-21-5p inhibitor	Survival rate (Hygro+/Hygro-) [%]	Two-sided Welch's t-test	0.63277
	HPHN-IntN-ON21<br>IntC-HPHC-ON21<br>IntC-PACCm-OFF21	NC inhibitor	miR-21-5p inhibitor	Survival rate (Hygro+/Hygro-) [%]	Two-sided Welch's t-test	0.00040
Fig.3f	Mock	NC inhibitor	miR-21-5p inhibitor	Survival rate (Blast+/Blast-) [%]	Two-sided Welch's t-test	0.80731
	BSR mRNA	NC inhibitor	miR-21-5p inhibitor	Survival rate (Blast+/Blast-) [%]	Two-sided Welch's t-test	0.87472
	BSR-ON21	NC inhibitor	miR-21-5p inhibitor	Survival rate (Blast+/Blast-) [%]	Two-sided Welch's t-test	0.42634
	BSRN-IntN-ON21<br>IntC-BSRC-ON21<br>IntC-PACCm-OFF21	NC inhibitor	miR-21-5p inhibitor	Survival rate (Blast+/Blast-) [%]	Two-sided Welch's t-test	0.00081
Fig.3h	Mock	NC inhibitor	miR-21-5p inhibitor	Survival rate (GCV+/GCV-) [%]	Two-sided Welch's t-test	0.73695
	TKA266C mRNA	NC inhibitor	miR-21-5p inhibitor	Survival rate (GCV+/GCV-) [%]	Two-sided Welch's t-test	0.97956
	TKA266C-ON21	NC inhibitor	miR-21-5p inhibitor	Survival rate (GCV+/GCV-) [%]	Two-sided Welch's t-test	0.02819
	TKN-IntN-ON21<br>IntC-TKC-ON21<br>IntC-AGCm-OFF21	NC inhibitor	miR-21-5p inhibitor	Survival rate (GCV+/GCV-) [%]	Two-sided Welch's t-test	0.00494
Fig. 5b	Cas9 mRNA	sgRNA+, NC inhibitor	sgRNA+, miR-21-5p inhibitor	EGFP-positive population [%]	Two-sided Welch's t-test	0.95966
	Cas9-ON21	sgRNA+, NC inhibitor	sgRNA+, miR-21-5p inhibitor	EGFP-positive population [%]	Two-sided Welch's t-test	0.03185
	Cas9N-IntN-ON21<br>IntC-Cas9C-ON21	sgRNA+, NC inhibitor	sgRNA+, miR-21-5p inhibitor	EGFP-positive population [%]	Two-sided Welch's t-test	0.00052
	Cas9N-IntN-ON21<br>IntC-Cas9C-ON21<br>IntC-AGCm-OFF21 x3	sgRNA+, NC inhibitor	sgRNA+, miR-21-5p inhibitor	EGFP-positive population [%]	Two-sided Welch's t-test	0.00089
	Cas9N-IntN-ON21<br>IntC-Cas9C-ON21<br>IntC-AGCm-OFF21 x6	sgRNA+, NC inhibitor	sgRNA+, miR-21-5p inhibitor	EGFP-positive population [%]	Two-sided Welch's t-test	0.00029
Fig. 5c	Cas9 mRNA	sgRNA+, NC inhibitor	sgRNA+, miR-302a-5p inhibitor	EGFP-positive population [%]	Two-sided Welch's t-test	0.54650
	Cas9-ON302a	sgRNA+, NC inhibitor	sgRNA+, miR-302a-5p inhibitor	EGFP-positive population [%]	Two-sided Welch's t-test	0.73453
	Cas9N-IntN-ON302a<br>IntC-Cas9C-ON302a	sgRNA+, NC inhibitor	sgRNA+, miR-302a-5p inhibitor	EGFP-positive population [%]	Two-sided Welch's t-test	0.59490

	Cas9N-IntN-ON302a IntC-Cas9C-ON302a IntC-AGCm-OFF302a x3	sgRNA+, NC inhibitor	sgRNA+, miR-302a-5p inhibitor	EGFP-positive population [%]	Two-sided Welch's t-test	1.71E-05
	Cas9N-IntN-ON302a IntC-Cas9C-ON302a IntC-AGCm-OFF302a x6	sgRNA+, NC inhibitor	sgRNA+, miR-302a-5p inhibitor	EGFP-positive population [%]	Two-sided Welch's t-test	0.00108
Fig. 5e	Cas9 mRNA	sgRNA+, miR-21-5p mimic	sgRNA+, miR-21-5p inhibitor	EGFP-positive population [%]	Two-sided Welch's t-test	0.95492
	Cas9-ON21	sgRNA+, miR-21-5p mimic	sgRNA+, miR-21-5p inhibitor	EGFP-positive population [%]	Two-sided Welch's t-test	0.01016
	Cas9N-IntN-ON21 IntC-Cas9C-ON21	sgRNA+, miR-21-5p mimic	sgRNA+, miR-21-5p inhibitor	EGFP-positive population [%]	Two-sided Welch's t-test	0.00426
	Cas9N-IntN-ON21 IntC-Cas9C-ON21 IntC-AGCm-OFF21 x3	sgRNA+, miR-21-5p mimic	sgRNA+, miR-21-5p inhibitor	EGFP-positive population [%]	Two-sided Welch's t-test	0.00729
	Cas9N-IntN-ON21 IntC-Cas9C-ON21 IntC-AGCm-OFF21 x6	sgRNA+, miR-21-5p mimic	sgRNA+, miR-21-5p inhibitor	EGFP-positive population [%]	Two-sided Welch's t-test	0.01022
Fig.6b	miR-21-5p mimic 0 nM	Normal toggle-like system	Split toggle-like system	Normalized fluorescence intensity (hmAG1)	Two-sided Welch's t-test	0.00202
	miR-21-5p mimic 2 nM	Normal toggle-like system	Split toggle-like system	Normalized fluorescence intensity (iRFP670)	Two-sided Welch's t-test	0.02491
Fig. 7b	NOR	[00]	[10]	Normalized hmAG1 intensity	Two-sided Dunnett's test	3.72E-11
		[00]	[01]			4.10E-11
		[00]	[11]			2.10E-13
	A AND NOT B	[10]	[00]	Normalized hmAG1 intensity	Two-sided Dunnett's test	6.31E-11
		[10]	[01]			1.19E-13
		[10]	[11]			3.83E-10
	NOT A AND B	[01]	[00]	Normalized hmAG1 intensity	Two-sided Dunnett's test	2.99E-10
		[01]	[10]			5.41E-12
		[01]	[11]			7.02E-12
	AND	[11]	[00]	Normalized hmAG1 intensity	Two-sided Dunnett's test	2.91E-09
		[11]	[10]			2.26E-10
		[11]	[01]			1.08E-08
Fig.8b	L7Ae/miR-21-NOR	[-, -]	[miR-21-5p, -]	Normalized hmAG1 intensity	Two-sided Dunnett's test	4.98E-12
		[-, -]	[-, L7Ae]			2.47E-10
		[-, -]	[miR-21-5p, L7Ae]			2.82E-15
	LIN28A/miR-302a-NOR	[-, -]	[miR-302a-5p, -]	Normalized hmAG1 intensity	Two-sided Dunnett's test	1.53E-13
		[-, -]	[-, LIN28A]			9.74E-09
		[-, -]	[miR-302a-5p, LIN28A]			7.33E-13
Fig.8c	-	AGQ63C-OFF302a	AGQ63C-NOR302a/LIN28A	Fold change in relative hmAG intensity (293FT/hiPSC)	Two-sided Welch's t-test	0.01018
	-	AGQ63C-OFFLIN28A	AGQ63C-NOR302a/LIN28A	Fold change in relative hmAG intensity (293FT/hiPSC)	Two-sided Welch's t-test	0.00842
Fig.8e	miR-21/302a/206 NOR gate	[000]	[100]	Normalized hmAG1 intensity	Two-sided Dunnett's test	1.88E-12
		[000]	[010]			3.04E-13
		[000]	[001]			6.44E-10
		[000]	[110]			9.21E-10
		[000]	[011]			3.13E-09
		[000]	[101]			3.31E-10
		[000]	[111]			6.96E-12
	miR-21/302a/206 AND gate	[111]	[000]	Normalized hmAG1 intensity	Two-sided Dunnett's test	1.19E-07
		[111]	[100]			3.72E-07
		[111]	[010]			8.54E-08
		[111]	[001]			1.79E-07
		[111]	[110]			3.17E-07
		[111]	[011]			1.63E-07
		[111]	[101]			6.83E-07
Fig.S2	-A100	miR-21-5p mimic	NC mimic	Relative hmAG1 intensity [a.u.]	Two-sided Welch's t-test	0.89212
	-A100-t21-aK-u5g	miR-21-5p mimic	NC mimic	Relative hmAG1 intensity [a.u.]	Two-sided Welch's t-test	0.00536

	-A100-t21-DE-u5g	miR-21-5p mimic	NC mimic	Relative hmAG1 intensity [a.u.]	Two-sided Welch's t-test	2.68E-05
	-A100-t21-aK-DE-u5g	miR-21-5p mimic	NC mimic	Relative hmAG1 intensity [a.u.]	Two-sided Welch's t-test	0.00699
	-A100-t21-DE-aK-u5g	miR-21-5p mimic	NC mimic	Relative hmAG1 intensity [a.u.]	Two-sided Welch's t-test	0.00121
	-	-A100-t21-DE-u5g	-A100-t21-DE-aK-u5g	ON/OFF ratio of relative hmAG1 intensity [a.u.]	Two-sided Welch's t-test	0.63925
**Fig.S6c (HeLa)**	Puromycin (+)	PAC mRNA	PAC-ON302a	Viability [%]	Two-sided Welch's t-test	0.46504
	Puromycin (+)	PAC mRNA	PACN-IntN-ON302a<br>IntC-PACC-ON302a<br>IntC-PACCm-OFF302a<br>x4	Viability [%]	Two-sided Welch's t-test	0.00108
**Fig.S6c (hiPSC)**	Puromycin (+)	PAC mRNA	PAC-ON302a	Viability [%]	Two-sided Welch's t-test	0.47986
	Puromycin (+)	PAC mRNA	PACN-IntN-ON302a<br>IntC-PACC-ON302a<br>IntC-PACCm-OFF302a<br>x4	Viability [%]	Two-sided Welch's t-test	0.67027
**Fig.S11b**	-	AGP131S-bN + bC	AGV153S-bN + bC	Normalized hmAG1 intensity	Two-sided Welch's t-test	0.00043
	-	AGV153S-bN + bC	AGG167S-bN + bC	Normalized hmAG1 intensity	Two-sided Welch's t-test	0.10330
	-	AGP131S-bN + bC	AGG167S-bN + bC	Normalized hmAG1 intensity	Two-sided Welch's t-test	0.00209
**Fig.S12**	-	AGQ63C	AGQ63C, G65A	Viability [%]	Two-sided Welch's t-test	0.97599
	-	AGQ63C	AGN-IntN<br>IntC-AGC	Viability [%]	Two-sided Welch's t-test	0.68266
	-	AGN-IntN<br>IntC-AGC	AGN-IntN<br>IntC-AGCm	Viability [%]	Two-sided Welch's t-test	0.51423

Supplementary References

1. Li, Y., Song, M.-G. & Kiledjian, M. Transcript-specific decapping and regulated stability by the human Dcp2 decapping protein. *Mol. Cell. Biol.* **28**, 939–948 (2008).
2. Song, M.-G. & Kiledjian, M. 3’ Terminal oligo U-tract-mediated stimulation of decapping. *RNA* **13**, 2356–2365 (2007).
3. Jillette, N., Du, M., Zhu, J. J., Cardoz, P. & Cheng, A. W. Split selectable markers. *Nat. Commun.* **10**, 1–8 (2019).
4. Massoud, T. F., Paulmurugan, R. & Gambhir, S. S. A molecularly engineered split reporter for imaging protein-protein interactions with positron emission tomography. *Nat. Med.* **16**, 921–926 (2010).
5. Aranko, A. S., Oeemig, J. S., Kajander, T. & Iwaï, H. Intermolecular domain swapping induces intein-mediated protein alternative splicing. *Nat. Chem. Biol.* **9**, 616–622 (2013).
6. Kutrowska, B. W., Narczyk, M., Buszko, A., Bzowska, A. & Clark, P. L. Folding and unfolding of a non-fluorescent mutant of green fluorescent protein. *J. Phys. Condens. Matter* 285223 (2007).
7. Filonov, G. S. & Verkhusha, V. V. A near-infrared BiFC reporter for in vivo imaging of protein-protein interactions. *Chem. Biol.* **20**, 1078–1086 (2013).
8. Caputo, A. T. *et al.* Structure-guided selection of puromycin N-acetyltransferase mutants with enhanced selection stringency for deriving mammalian cell lines expressing recombinant proteins. *Sci. Rep.* **11**, 5247 (2021)
9. Truong, D.-J. J. *et al.* Development of an intein-mediated split-Cas9 system for gene therapy. *Nucleic Acids Res.* **43**, 6450–6458 (2015).
