# Supplementary material for: Split RNA switch orchestrates pre- and post-translational control to enable cell type-specific gene expression
Source: Nat Commun. 2025 Jul 1;16:5362. doi: 10.1038/s41467-025-60392-2 (PMC12219281; doi:10.1038/s41467-025-60392-2)
Supplement: Supplementary file 2 — Reporting Summary [file 41467_2025_60392_MOESM2_ESM.pdf]

nature portfolio

Corresponding author(s): Hirohide Saito, HIrohisa Ohno

Last updated by author(s): May 15, 2025

Reporting Summary

Nature Portfolio wishes to improve the reproducibility of the work that we publish. This form provides structure for consistency and transparency in reporting. For further information on Nature Portfolio policies, see our Editorial Policies and the Editorial Policy Checklist.

Statistics

For all statistical analyses, confirm that the following items are present in the figure legend, table legend, main text, or Methods section.

n/a	Confirmed	
☐	☒	The exact sample size (*n*) for each experimental group/condition, given as a discrete number and unit of measurement
☐	☒	A statement on whether measurements were taken from distinct samples or whether the same sample was measured repeatedly
☐	☒	The statistical test(s) used AND whether they are one- or two-sided<br>*Only common tests should be described solely by name; describe more complex techniques in the Methods section.*
☒	☐	A description of all covariates tested
☐	☒	A description of any assumptions or corrections, such as tests of normality and adjustment for multiple comparisons
☐	☒	A full description of the statistical parameters including central tendency (e.g. means) or other basic estimates (e.g. regression coefficient) AND variation (e.g. standard deviation) or associated estimates of uncertainty (e.g. confidence intervals)
☐	☒	For null hypothesis testing, the test statistic (e.g. *F*, *t*, *r*) with confidence intervals, effect sizes, degrees of freedom and *P* value noted<br>*Give P values as exact values whenever suitable.*
☒	☐	For Bayesian analysis, information on the choice of priors and Markov chain Monte Carlo settings
☒	☐	For hierarchical and complex designs, identification of the appropriate level for tests and full reporting of outcomes
☒	☐	Estimates of effect sizes (e.g. Cohen's *d*, Pearson's *r*), indicating how they were calculated

Our web collection on statistics for biologists contains articles on many of the points above.

Software and code

Policy information about availability of computer code

Data collection	Cell images were obtained by CellVoyager CQl/CQl Software Version 1.07.01.01 (Yokogawa Electric Corporation). Flow cytometry data were obtained by CytoFLEX Flow Cytometer S/CytExpert 2.4 (Beckman Coulter) or BD Accuri C6/Cflow Plus Software Version 1.0.227.4 (BD Biosciences).
Data analysis	Fluorescent images were analyzed using ImageJ (version l.53t). Flowjo (version 10.8.2) was used to visualize flow cytometry data.

For manuscripts utilizing custom algorithms or software that are central to the research but not yet described in published literature, software must be made available to editors and reviewers. We strongly encourage code deposition in a community repository (e.g. GitHub). See the Nature Portfolio guidelines for submitting code & software for further information.

Data

Policy information about availability of data

All manuscripts must include a data availability statement. This statement should provide the following information, where applicable:

- Accession codes, unique identifiers, or web links for publicly available datasets
- A description of any restrictions on data availability
- For clinical datasets or third party data, please ensure that the statement adheres to our policy

All primary data can be accessed in the Source Data file. All other data including protein sequences and statics are available within the article and Supplementary information. Materials such as plasmids are available from the corresponding author upon reasonable request. No large datasets were generated, and no code was utilized in this manuscript.

Research involving human participants, their data, or biological material

Policy information about studies with human participants or human data. See also policy information about sex, gender (identity/presentation), and sexual orientation and race, ethnicity and racism.

Reporting on sex and gender	n/a
Reporting on race, ethnicity, or other socially relevant groupings	n/a
Population characteristics	n/a
Recruitment	n/a
Ethics oversight	n/a

Note that full information on the approval of the study protocol must also be provided in the manuscript.

Field-specific reporting

Please select the one below that is the best fit for your research. If you are not sure, read the appropriate sections before making your selection.

☒ Life sciences ☐ Behavioural & social sciences ☐ Ecological, evolutionary & environmental sciences

For a reference copy of the document with all sections, see nature.com/documents/nr-reporting-summary-flat.pdf

Life sciences study design

All studies must disclose on these points even when the disclosure is negative.

Sample size	All data had at least 3 biological replicates (n>=3) in order to reduce the chances that results are due to variability or external factors.
Data exclusions	No data was excluded from the analysis.
Replication	All experiments were reliably reproduced in independent settings for at least three times.
Randomization	Randomization was not performed. Treatment conditions (e.g., miRNA mimic/inhibitor and RNA switch transfection) were manually assigned to specific wells, and the well identities were known during the experiment. To minimize potential bias, all samples were processed in parallel, and transfections were performed at the same time under identical conditions.
Blinding	Blinding was not performed because all experimental conditions were applied to genetically identical cells and data were acquired using automated instruments (flow cytometer, microplate reader, and cell imager). Quantitative analyses were conducted using standardized, automated procedures (FlowJo, Excel, and ImageJ), minimizing the potential for operator bias.

Reporting for specific materials, systems and methods

We require information from authors about some types of materials, experimental systems and methods used in many studies. Here, indicate whether each material, system or method listed is relevant to your study. If you are not sure if a list item applies to your research, read the appropriate section before selecting a response.

Materials & experimental systems

n/a	Involved in the study
[x]	[ ] Antibodies
[ ]	[x] Eukaryotic cell lines
[x]	[ ] Palaeontology and archaeology
[x]	[ ] Animals and other organisms
[x]	[ ] Clinical data
[x]	[ ] Dual use research of concern
[x]	[ ] Plants

Methods

n/a	Involved in the study
[x]	[ ] ChIP-seq
[ ]	[x] Flow cytometry
[x]	[ ] MRI-based neuroimaging

Eukaryotic cell lines

Policy information about cell lines and Sex and Gender in Research

Cell line source(s)	HEK293FT (invitrogen, R70007)<br>Hela CCL2 (ATCC)<br>201B7 (CiRA)<br>201B7 (RIKEN)<br>201B7 (from Dr. Knut Woltjen (Kyoto university))<br>HEK293T (from Dr. Hotta Akitsu (Kyoto university))
Authentication	Cells were not authenticated.
Mycoplasma contamination	Mycoplasma is regularly tested and the cell lines used in this study were negative for mycoplasma contamination.
Commonly misidentified lines (See ICLAC register)	No commonly misidentified cell lines were used.

Plants

Seed stocks	n/a
Novel plant genotypes	n/a
Authentication	n/a

Flow Cytometry

Plots

Confirm that:

- [x] The axis labels state the marker and fluorochrome used (e.g. CD4-FITC).
- [x] The axis scales are clearly visible. Include numbers along axes only for bottom left plot of group (a 'group' is an analysis of identical markers).
- [x] All plots are contour plots with outliers or pseudocolor plots.
- [x] A numerical value for number of cells or percentage (with statistics) is provided.

Methodology

Sample preparation	For flow cytometry analysis, cultured and transfected cells were washed once with PBS. Then, the cells were trypsinized and resuspended in culture medium. The resuspended cells were filtered through a mesh filter before flow cytometry analysis.
Instrument	CytoFLEX (Beckman Coulter) and BD Accuri C6 (BD Bioscience)
Software	Flow cytometry data were obtained by CytExpert 2.4 (Beckman Coulter) or Cflow Plus Software Version 1.0.227.4 (BD Biosciences). The flow cytometry data were analyzed using FlowJo software (version 10.8.2).
Cell population abundance	At least 10,000 living cells were analyzed.

Gating strategy	The cells were gated based on FSC-H/SSC-H to exclude the cell debris. Next, the cells were gated based on FSC-A/FSC-H to exclude the doublets. The remaining PI-positive events were used for subsequent analysis.

☒ Tick this box to confirm that a figure exemplifying the gating strategy is provided in the Supplementary Information.
